# Diagnostic Failure in Invasive Fungal Infections: Causes, Clinical Consequences, and Mitigation Strategies

**DOI:** 10.3390/jof12070498

**Published:** 2026-07-08

**Authors:** Pilar Rivas-Pinedo, José Millán Oñate Gutiérrez

**Affiliations:** 1 Medical and Diagnostic Mycology Group, Department of Microbiology, Faculty of Medicine, Universidad Nacional de Colombia, Bogotá 111321, Colombia; 2 One Health Research Group for Infectious Diseases, Keralty, Department of Internal Medicine, Clínica Colsanitas S.A., Clínica Sebastián de Belalcázar, Cali 760045, Colombia; 3 Infectious Diseases Service, Clínica Imbanaco, Cali 760042, Colombia; 4 Clinical Research and Education Group (GIEDCO), Clínica de Occidente, Cali 760035, Colombia

**Keywords:** invasive fungal infections, diagnostic failure, delayed diagnosis, antifungal stewardship, diagnostic stewardship, invasive candidiasis, invasive aspergillosis, mucormycosis, fungal biomarkers, high-risk populations

## Abstract

Diagnostic failure in invasive fungal infections (IFIs) remains a relevant and underrecognized cause of mortality, morbidity, delayed therapy, unnecessary antifungal exposure, and pharmacological selective pressure. Although major advances have been achieved in biomarkers, rapid diagnostic tests, molecular methods, imaging studies, and microbiological identification, timely diagnosis continues to be influenced by the interaction among host factors, pathogen-related factors, diagnostic tools, and healthcare system–related factors. This narrative review analyzes diagnostic failure in IFIs as a dynamic process that includes delayed, incorrect, and incomplete diagnosis. It examines its determinants and consequences in high-risk populations—critically ill patients, patients with hematologic diseases or hematopoietic stem cell transplant recipients, and neonates—as well as in invasive candidiasis, aspergillosis, mucormycosis, cryptococcosis, endemic mycoses, and infections caused by rare or emerging fungi. It also reviews how delayed sampling, decontextualized interpretation of biomarkers, incomplete microbiological identification, absence of antifungal susceptibility testing when clinically relevant, and fragmentation between clinical and laboratory teams contribute to adverse outcomes. Finally, it proposes a diagnostic-centered antifungal stewardship framework (AFSP-Dx) based on syndromic bundles, population-specific diagnostic algorithms, 48–72 h reassessment, and auditable indicators intended to support earlier recognition, more precise therapeutic decisions, and rational antifungal use.

## 1. Introduction

Invasive fungal infections (IFI) continue to represent an important and frequently underrecognized cause of morbidity and mortality in critically ill, immunocompromised, and high-risk neonatal patients. Despite advances in antifungal therapy, biomarkers, imaging, rapid diagnostic tests, and molecular methods, timely recognition of these infections remains challenging. Within this framework, diagnostic failure—understood as delayed, incorrect, or incomplete diagnosis—constitutes a major determinant of adverse clinical outcomes, with direct impact on mortality, length of hospitalization, unnecessary antifungal exposure, and selection of antifungal resistance [1,2,3,4,5,6,7,8].

Unlike many bacterial infections, IFIs often present with nonspecific clinical manifestations and frequently overlap with noninfectious inflammatory processes or with concomitant bacterial and viral infections. In addition, several conventional diagnostic tools have limited sensitivity, prolonged turnaround times, or variable performance depending on the type of host, the anatomical site of infection, and prior antifungal exposure. These limitations contribute to clinically significant diagnostic delays even in tertiary-care centers [4,5,9,10,11,12].

Although diagnostic failure in invasive fungal infections may occur across a broader range of at-risk populations, including solid organ transplant recipients and patients with advanced HIV infection, chronic lung disease, liver disease, or other immunocompromising conditions, this review focuses on three high-risk clinical settings: patients in the intensive care unit (ICU), patients with hematologic malignancies or hematopoietic stem cell transplant (HSCT) recipients, and critically ill neonates. These settings were selected because they present distinct diagnostic challenges and differ in pretest probability, disease progression, diagnostic test performance, and the clinical consequences of delayed diagnosis. Applying the same diagnostic approach across these populations may therefore result in both underdiagnosis and unnecessary antifungal treatment [1,2,5,13,14,15,16,17].

In the ICU, invasive candidiasis (IC) and ICU-associated aspergillosis commonly develop in the context of sepsis, multiorgan failure, mechanical ventilation (MV), the presence of invasive devices, and prior exposure to broad-spectrum antibiotics. In this population, clinical nonspecificity, the low sensitivity of blood cultures in certain settings, and the complex interpretation of fungal biomarkers may delay initiation of appropriate antifungal therapy. In turn, diagnostic uncertainty favors prolonged empiric antifungal use in patients without ultimately documented IFI [4,18,19,20,21].

In hematologic patients and HSCT recipients, diagnostic failure acquires an additional dimension. Prolonged neutropenia, cellular immunosuppression, antifungal prophylaxis, and atypical clinical presentations modify both clinical expression and the performance of cultures and biomarkers. In addition, these patients are at increased risk of developing breakthrough fungal infections, infections caused by Mucorales, rare molds, emerging yeasts, and pathogens with clinically relevant antifungal resistance, in which identification at the complex or species level and antifungal susceptibility profiles may directly influence therapeutic management [19,22,23,24,25].

The neonatal population, particularly preterm and very low birth weight (VLBW) neonates, represents another critical scenario. IC may present as late-onset neonatal sepsis (LOS), indistinguishable from bacterial etiologies, whereas the small blood volumes available reduce blood culture yield. The limited validation of biomarkers and molecular tests in this population, together with the risk of central nervous system (CNS) involvement and other deep-seated foci, contributes to diagnostic delays with consequences for mortality and neurodevelopment [14,26,27,28,29,30].

From the perspective of public health and antifungal stewardship programs (AFSP), diagnostic failure has implications that extend beyond delayed therapy. Diagnostic uncertainty may perpetuate prolonged empiric treatment, increase drug toxicity, raise costs, reduce the yield of subsequent diagnostic testing, and promote selective pressure on the hospital fungal ecology. Consequently, modern AFSPs should not focus solely on controlling antifungal consumption, but also on improving the quality of the diagnostic process through clinical pathways, clinical–laboratory integration, structured reassessment, and auditable indicators [17,23,31,32].

Despite its relevance, diagnostic failure in IFI has been addressed in a fragmented manner, frequently from the perspective of specific pathogens, particular populations, or isolated diagnostic tests. Therefore, there is a need for integrative reviews that comprehensively analyze its causes, clinical consequences, and mitigation strategies, incorporating both the microbiological and organizational dimensions of diagnosis [4,5,31,32].

Accordingly, this narrative review critically examines diagnostic failure in IFIs within these three high-risk clinical settings. It addresses host-, pathogen-, and healthcare system-related determinants; the clinical consequences of diagnostic failure; and the limitations of current diagnostic tools. On the basis of this synthesis, the authors propose a diagnostic-centered antifungal stewardship framework (AFSP-Dx) incorporating diagnostic bundles, population-specific pathways, early reassessment, and quality indicators as potential implementation tools.

### Search Strategy and Selection Criteria

This manuscript was developed as a narrative review. A targeted literature search was conducted in PubMed/MEDLINE, Scopus, and Web of Science, complemented by manual review of the references cited in relevant articles. Publications from 2005 through March 2026 were included, as well as earlier seminal studies considered essential for contextualizing the evolution of the field.

The search strategy combined controlled vocabulary and free-text terms related to invasive fungal infections, diagnostic failure, delayed diagnosis, candidemia, aspergillosis, mucormycosis, cryptococcosis, endemic mycoses, fungal biomarkers, molecular diagnostics, rapid diagnostic tests, antifungal stewardship, intensive care units, hematology, hematopoietic stem cell transplantation, and neonatology.

Priority was given to international guidelines, consensus documents, systematic reviews, meta-analyses, multicenter studies, and clinically applicable cohort studies. Sources were selected according to thematic relevance, methodological rigor, and currency. Primary studies were used to support specific diagnostic and clinical observations, whereas systematic reviews, guidelines, and consensus documents were used to synthesize the broader evidence and contextualize current diagnostic and stewardship recommendations. The findings were integrated through a qualitative narrative synthesis aimed at identifying the causes, clinical consequences, and potential mitigation strategies associated with diagnostic failure in IFIs. The narrative synthesis distinguishes findings drawn from the published literature from the conceptual and operational proposals developed by the authors. The AFSP-Dx framework, proposed bundles, and selected quality indicators are presented as implementation-oriented tools for local adaptation and future evaluation, rather than as formally validated standards.

## 2. Conceptual Framework of Diagnostic Failure in IFI

IFI continues to be associated with a high burden of morbidity and mortality, particularly when clinical recognition and initiation of appropriate antifungal therapy are delayed. Unlike other infections, their diagnosis often requires sequential integration of host risk factors, nonspecific clinical manifestations, radiologic findings, conventional microbiology, biomarkers, and, in certain cases, invasive procedures. In IFI, diagnostic failures rarely result from a single cause and frequently reflect cumulative weaknesses throughout the continuum of care [1,4,5,7,8,22].

Traditionally, discussion has focused on the analytical performance of individual tests. However, in clinical practice, the timeliness of clinical suspicion, appropriate selection of patients who should undergo diagnostic evaluation, sample quality, and institutional capacity to translate results into therapeutic decisions may be just as critical as the test itself. Therefore, diagnostic failure should be understood as a systemic and dynamic problem, particularly relevant to contemporary antifungal stewardship (AFS) and continuous quality improvement programs [17,31,32,33,34,35].

From this perspective, the authors propose an operational framework for understanding how diagnostic failure occurs, what its principal components are, and how it may be measured. This framework is intended as a conceptual and implementation-oriented model and does not replace established clinical definitions or validated diagnostic criteria.

### 2.1. Operational Definition of Diagnostic Failure

In the context of IFI, diagnostic failure may be defined as the inability to recognize a clinically relevant fungal infection in a timely, accurate, and sufficiently comprehensive manner, such that this failure leads to suboptimal therapeutic decisions or adverse clinical outcomes [1,2,3,7,8].

From both clinical and AFSP perspectives, this concept should not be limited to the absence of microbiological confirmation. In practice, it represents a dynamic continuum that includes delayed clinical suspicion, late or inappropriate ordering of diagnostic tests, misinterpretation of results, and omission of relevant diagnostic information, even when diagnostic tools are available [4,5,17,31,32].

Therefore, the problem does not always lie in the lack of diagnostic technology, but frequently in the discordance among clinical risk, timing of sampling, diagnostic test performance, and integration of findings into clinical decision-making. This perspective is particularly important in IFI, where clinical presentation is often nonspecific and no single diagnostic test has sufficient performance to exclude or confirm all clinical scenarios [4,5,22,36,37,38].

Recognizing diagnostic failure as a multifactorial phenomenon shifts the focus from the individual test to the diagnostic process as a whole. This is particularly relevant for AFSPs and for strategies aimed at reducing mortality, preventable drug toxicity, and unnecessary empiric therapy [17,31,32].

This approach is also consistent with contemporary diagnostic safety frameworks, which understand diagnostic error as a multifactorial process influenced by cognitive, organizational, technological, and communication-related factors, rather than as an isolated failure of a specific diagnostic test [34,35]. In addition, this perspective facilitates evaluation of diagnostic performance through process indicators—such as time to suspicion, time to sample collection, or time to appropriate therapy—rather than solely by the availability of a specific diagnostic test, which is particularly useful in settings with heterogeneous resources [9,24,33,35,39,40].

### 2.2. Components of Diagnostic Failure

Diagnostic failure in IFI does not constitute a single entity, but rather a set of clinical patterns in which the diagnostic process loses sufficient timeliness, accuracy, or depth to guide appropriate clinical decisions. In clinical practice, these failures rarely occur in isolation; they frequently overlap and evolve in cascade, beginning with delayed suspicion, followed by incorrect interpretations or incomplete microbiological characterization. Analyzing their components separately helps conceptually organize the problem and facilitates the design of institutional continuous improvement indicators [31,32,34,35].

From an operational perspective, three domains account for most clinically relevant events: delayed diagnosis, when IFI is recognized after an optimal therapeutic window; incorrect diagnosis, when infection is misinterpreted or a nonexistent disease is overestimated; and incomplete diagnosis, when partial recognition exists but remains insufficient to guide definitive management. Although conceptually distinguishable, these components share structural determinants, including low clinical suspicion, diagnostic limitations, fragmentation between laboratory and treating teams, and absence of AFSP-Dx pathways [4,5,22,31,32].

An IFI may also remain unrecognized when the diagnostic evaluation yields false-negative results because of a low fungal burden, tissue-localized infection, an inadequate specimen type or volume, prior antifungal exposure, or limited test sensitivity in the host population and clinical setting under evaluation. Negative cultures, biomarkers, imaging findings, or molecular tests should therefore not be interpreted in isolation when the pretest probability remains high [4,5,22,31,32].

Finally, this classification is not intended to replace existing microbiological or clinical definitions of IFI, but rather to complement their utility from a healthcare delivery perspective. Whereas standardized criteria allow classification of diagnostic probability for research or clinical practice, the operational approach to diagnostic failure helps identify at which point in the process the opportunity is lost: before infection is suspected, during interpretation of findings, or while attempting to define etiology, extent of disease, and antifungal susceptibility [22,31,41].

#### 2.2.1. Delayed Diagnosis

Delayed diagnosis corresponds to the identification of an IFI at an advanced stage of the infectious process, when systemic complications, anatomic progression of disease, or potentially irreversible tissue damage have already developed. It probably constitutes the most frequent form of diagnostic failure and the one most consistently associated with increased mortality and worse clinical outcomes [7,24,39].

In practice, the mechanisms leading to delay vary according to the population being treated. In the ICU, the predominant factors include nonspecific clinical manifestations, coexistence with bacterial sepsis, initially negative blood cultures, and the complex interpretation of biomarkers. In hematology and HSCT, prior antifungal prophylaxis may alter the clinical presentation and reduce the performance of certain diagnostic tests. In neonates, low initial clinical suspicion, overlap with late-onset bacterial sepsis, and limitations inherent to microbiological sampling contribute substantially to diagnostic delay [11,12,22,26,27,28,29,42,43,44].

In candidemia, delayed initiation of appropriate antifungal therapy has consistently been associated with increased mortality. In IA, delayed recognition and treatment may permit further disease progression and are likewise associated with poorer outcomes. Time to clinical suspicion and time to appropriate antifungal therapy should therefore be considered key quality-of-care indicators in patients with IFIs [24,31,32,39].

#### 2.2.2. Incorrect Diagnosis

Incorrect diagnosis occurs when an IFI is misinterpreted as another clinical process, or when noninvasive microbiological findings are assumed to represent true infection. Both situations have clinical relevance [11,13,31,33,45].

Among the most frequent errors are classification of an IFI as colonization, contamination, an alternative bacterial or viral infection, or a noninfectious inflammatory process. Conversely, overdiagnosis may also occur when colonization or isolated laboratory findings are incorrectly interpreted as established invasive disease [13,31,33,46].

This component of diagnostic failure is particularly important in the ICU, where isolation of *Candida* spp. from respiratory samples may be underestimated or, conversely, may trigger excessive clinical concern. In hematology/HSCT, radiologic overlap between IA, viral pneumonias, alveolar hemorrhage, or pulmonary toxicity may delay appropriate clinical decision-making. In neonates, candidemia may initially be confused with persistent bacterial sepsis [19,20,26,27,28,29,46].

The consequences include both undertreatment, when the IFI is not recognized, and overtreatment, when antifungal agents are administered in the absence of true invasive disease. Both extremes are associated with clinical harm, avoidable drug toxicity, and inefficient use of resources [18,31,32].

#### 2.2.3. Incomplete Diagnosis

Incomplete diagnosis occurs when the presence of an IFI is recognized, but the information obtained is insufficient to optimally guide clinical management. This may include failure to precisely identify the etiologic agent, lack of definition of the anatomical extent of infection, failure to detect concomitant coinfections, or omission of emerging pathogens with relevant therapeutic implications [9,10,19,20,23,24,43,47,48].

This scenario is common in patients previously exposed to antifungal agents, in infections caused by non-*Candida* yeasts or less common molds, and in episodes of breakthrough infection or fungal coinfection [10,19,23,24,25,47,48,49,50,51,52,53,54].

From a clinical perspective, incomplete diagnosis often results in suboptimal treatment, delays in switching antifungal classes, the need for prolonged empiric therapy, and greater selective pressure on the hospital fungal ecology. Consequently, recognizing the presence of an IFI alone is insufficient; it is also necessary to define with adequate precision which pathogen is involved, the anatomical extent of the infection, and the factors that may influence the therapeutic response [14,23,31,32,42,43].

Together, these three components illustrate that diagnostic failure in IFIs rarely depends on a single isolated error, but rather on a sequence of suboptimal decisions throughout the continuum of care. The operational categories of diagnostic failure and their main practical implications are summarized in Table 1.

### 2.3. Differentiation Between Diagnostic Failure and Diagnostic Limitation

It is essential to distinguish between diagnostic failure and diagnostic limitation, as both concepts represent different problems and require different interventions. This distinction prevents attributing all errors to the lack of diagnostic technology and allows recognition of when the problem lies in resource availability and when it is related to how those resources are integrated into the clinical process.

Diagnostic limitation refers to the objective absence of the tools, infrastructure, or resources required to establish a timely and accurate diagnosis. Common examples include lack of access to fungal PCR, serum biomarkers, advanced imaging, specialized histopathology, or high-resolution mycological identification [6,11,13,17,31,46].

In contrast, diagnostic failure occurs when such tools are available but are not used appropriately, are ordered too late, are applied to patients with low pretest probability, or are interpreted incorrectly within the specific clinical context [12,31,32,45]. In other words, diagnostic limitation reflects an availability gap, whereas diagnostic failure reflects a gap in clinical and organizational execution.

This distinction is particularly relevant for AFSPs, in which the objective should not focus solely on incorporating new technologies, but also on improving integration among clinical suspicion, test selection, interpretation of results, and therapeutic decision-making [17,31,32].

Operationally, addressing a diagnostic limitation usually requires investment, access to existing technology, and strengthening of laboratory capacity or referral networks. In contrast, addressing diagnostic failure requires process redesign: defining clinical triggers, standardizing sampling pathways, improving clinical-microbiology communication, and establishing mandatory reassessment checkpoints. Both dimensions may coexist, but distinguishing between them allows prioritization of more realistic and measurable interventions.

### 2.4. Diagnostic Failure as a Cross-Cutting Phenomenon

Diagnostic failure in IFI is not exclusive to a particular pathogen or specific population, but rather a cross-cutting phenomenon that adopts different manifestations depending on the biology of the microorganism, the host profile, and the healthcare setting.

In IC, it commonly manifests as excessive reliance on blood cultures, suboptimal sensitivity during early stages, and delayed therapy. In IA, the predominant issues include low sensitivity of certain tests in non-classical settings, difficulty obtaining deep samples, and initial radiologic nonspecificity. In mucormycosis, the problem is related to the absence of useful serum biomarkers, the need for tissue confirmation, and the frequency of delayed or even postmortem diagnoses. In rare and emerging fungi, the principal challenge is usually delayed, incomplete, or incorrect identification, with direct impact on therapeutic selection [11,18,19,20,43,44,59,60].

This cross-cutting nature reinforces the need for a common conceptual framework that allows analysis of the diagnostic process beyond a single disease and facilitates strategies applicable across different clinical scenarios [17,31,32,61].

This common framework, however, does not imply using a single diagnostic algorithm for all patients. On the contrary, it allows recognition of shared patterns—delay, error, and incomplete diagnostic evaluation—and adaptation of these patterns to local epidemiology, baseline host risk, and the expected performance of each diagnostic tool.

### 2.5. Relevance of the Conceptual Framework for ICU, Hematology/HSCT, and Neonatology

The usefulness of this conceptual framework becomes particularly evident in high-risk populations, where diagnostic delay often has greater clinical impact [1,2,4].

In the ICU, it helps identify when sepsis without an evident source, unexplained respiratory deterioration, or persistent hemodynamic instability justifies intensifying the search for IFI rather than limiting evaluation to bacterial etiologies. In hematology and HSCT, it facilitates recognition of the limitations of traditional diagnostic algorithms in patients receiving antifungal prophylaxis, with atypical presentations or risk of breakthrough infections caused by molds not covered by prophylaxis. In neonates, it provides a useful framework for decision-making in persistent sepsis with negative cultures, particularly in preterm neonates with central venous catheters (CVCs), prolonged antibiotic exposure, or VLBW neonates [11,14,26,27,28,29,30,42,43,44].

Adopting clear operational definitions helps reduce interservice clinical variability, improve diagnostic timelines, optimize antifungal use, and prioritize measurable institutional interventions. In addition, these categories allow differentiation among failures of timeliness, interpretation, diagnostic depth, technological availability, and healthcare process execution, and are consistent with the need to adapt diagnostic criteria and AFS strategies to specific populations and clinical settings [21,31,37,41].

## 3. Determinants of Diagnostic Failure in IFIs

Diagnostic failure in IFI is a multifactorial phenomenon that emerges from the interaction among host-related factors, pathogen-related factors, available diagnostic tools, and healthcare system-related factors. It rarely results from a single isolated cause; more commonly, it represents the cumulative effect of successive barriers that delay clinical suspicion, limit acquisition of appropriate samples, reduce diagnostic test performance, or hinder integrated interpretation of results. The operational categories summarized in Table 1 provide a useful framework for interpreting these determinants [1,2,3,4,5,7,8,11].

These barriers acquire particular relevance in IFI because clinical presentation is often nonspecific, fungal burden may be low or localized, and many diagnostic tests show variable performance depending on the population evaluated, the anatomic site of infection, and prior antifungal exposure. Added to this are organizational factors such as unequal access to diagnostic technologies, prolonged processing times, fragmentation between clinical and laboratory teams, and the absence of standardized institutional diagnostic algorithms [4,5,6,11,31,32].

Understanding these determinants is essential not only to explain why diagnostic errors occur in high-risk patients, but also to design realistic mitigation strategies tailored to each clinical setting. From this perspective, diagnostic failure can be analyzed within three principal domains: host-related factors, pathogen-related factors, and healthcare system-related factors [8,17,31,32,34,35,61]. This approach allows transition from a model focused exclusively on the diagnostic test toward a broader model of healthcare performance, in which clinical suspicion, timing of sampling, institutional logistics, multidisciplinary interpretation, and therapeutic response capacity are inseparable components of the diagnostic process [31,32,61].

### 3.1. Host-Related Factors

Host characteristics constitute one of the most important determinants of diagnostic failure in IFI. They not only influence the risk of acquiring infection, but also the way infection manifests clinically, the utility of available diagnostic tools, and the speed at which organ damage progresses. Thus, the same pathogen may present very differently depending on the patient’s immune status, age, comorbidities, and therapeutic exposures [1,2,3,22].

Many diagnostic delays occur because classic models of infection—fever, localizing inflammation, and early positive cultures—are not reproduced in critically ill, immunosuppressed, or preterm neonatal patients. In these groups, IFI often manifests as nonspecific deterioration, progressive organ failure, or attenuated inflammation, which decreases initial suspicion and favors alternative attributions. Recognizing these host-related modifiers is essential for appropriately interpreting negative tests, ambiguous biomarkers, and atypical clinical presentations [4,5,31,62].

For practical purposes, host-related factors may be grouped into three particularly relevant domains: alterations in immune response, nonspecific or atypical clinical presentations, and prior antifungal exposure. These components interact with one another and frequently explain why IFI is diagnosed late or incompletely even in hospitals with access to advanced diagnostic tools.

#### 3.1.1. Alterations in Immune Response

IFIs occur predominantly in patients with significant alterations in innate immunity, adaptive immunity, or both, which modifies the usual clinical expression of fungal infection and complicates early recognition [1,2]. In many cases, the inflammatory response is insufficient, attenuated, or clinically atypical, such that classic signs of infection may be absent even in advanced invasive disease.

In the ICU, this phenomenon is commonly associated with sepsis-induced immune dysfunction, corticosteroid exposure, use of immunomodulatory agents, and immune paralysis associated with critical illness. In hematology and HSCT, prolonged neutropenia, quantitative or qualitative neutrophil dysfunction, profound lymphopenia, and the effects of targeted biologic therapies predominate. In neonates, particularly extremely low birth weight (ELBW) preterm infants, immunologic immaturity, functional complement deficiencies, and limited phagocytic capacity increase susceptibility to IFI and hinder the development of an evident clinical response [1,2,3,22,28,30,63].

These alterations help explain why some patients with disseminated IFI may present with minimal fever, poorly expressive inflammatory biomarkers, or nonspecific deterioration without clear localizing findings [3,8,11,14,22,43,45].

#### 3.1.2. Nonspecific or Atypical Clinical Presentations

The clinical manifestations of IFI frequently overlap with bacterial sepsis, systemic inflammatory response syndrome, or organ failure of other etiologies, thereby favoring diagnostic delays and errors in clinical attribution [4,5,9,14,43].

Diagnostic failure may begin before characteristic manifestations emerge. Early in the course of an IFI, symptoms and signs may be absent or nonspecific, and imaging findings may be normal, subtle, or insufficiently distinctive. Diagnostic suspicion should therefore not depend exclusively on overt clinical or radiologic abnormalities. In high-risk patients, host-related risk factors, relevant exposures, and an otherwise unexplained clinical course may justify further diagnostic evaluation before definitive clinical, radiologic, or microbiological evidence becomes available [4,5,22,31,32,42,43].

In the ICU, persistent or recurrent fever without an evident source is often initially attributed to multidrug-resistant bacteria, noninfectious complications, or intravascular devices. In hematologic patients, new pulmonary infiltrates may be interpreted as drug toxicity, alveolar hemorrhage, pulmonary edema, or viral infection before IFI is considered. In neonates, candidemia commonly presents as LOS that is clinically indistinguishable from bacterial causes [4,5,9,26,27,28,29,30,44,64].

Clinical nonspecificity requires maintaining a contextualized and dynamic diagnostic suspicion, particularly when clinical evolution is not consistent with the antibacterial therapy initiated [4,5,31].

#### 3.1.3. Prior Antifungal Exposure

Prior antifungal exposure, whether administered as prophylaxis or empiric therapy, may alter the clinical and microbiological context in which an IFI is investigated. Its effect varies according to the pathogen, host population, antifungal agent, duration of exposure, timing of sampling, and diagnostic method. Cultures and biomarkers obtained during or after antifungal therapy should therefore be interpreted in relation to these factors and the remaining clinical evidence, particularly when the pretest probability of IFI remains high [11,12,18,22,23,39,42,43,45,47,53,65].

This selective pressure favors infections caused by less susceptible species, non-*albicans* yeasts, emerging molds, or breakthrough infections, scenarios in which standard diagnostic algorithms may be insufficient [10,18,23,24,25,47,48,52,53,54,66,67].

This phenomenon is particularly relevant in hematologic patients receiving azole prophylaxis and in neonates hospitalized in neonatal ICUs with prior fluconazole exposure. In both groups, a negative microbiological result should be interpreted cautiously, especially when high clinical suspicion persists [22,26,27,28,30,68].

### 3.2. Pathogen-Related Factors

In addition to host conditions, multiple intrinsic characteristics of the microorganism contribute to diagnostic failure in IFI. Unlike many bacterial pathogens, fungi often exhibit biological patterns that hinder microbiological recovery, reduce the sensitivity of direct tests, and favor incomplete interpretations when the diagnostic approach is not adapted to the clinical context. Among these properties are low circulating burden, slow growth, increasing taxonomic diversity, intrinsic or acquired antifungal resistance, and the possibility of simultaneous coinfections [4,10,22,23,62,65,69,70].

In practice, these factors explain why a conventional diagnostic strategy may be sufficient in some clinical scenarios and clearly insufficient in others. They also justify the use of multimodal approaches integrating conventional microbiology, biomarkers, rapid diagnostic tests, imaging, histopathology, molecular methods when available, and serial clinical reassessment, particularly in high-risk patients or those with unexpected clinical evolution [6,13,19,20,22,46,55,59,69,71,72,73].

#### 3.2.1. Low Fungal Burden and Slow Growth

Unlike many bacteria, several fungi causing IFI may produce invasive disease with low circulating burden or predominantly tissue-based distribution, thereby limiting the performance of direct diagnostic methods applied to peripheral blood. This phenomenon is particularly evident in IA, where fungemia is uncommon, and in deep forms of candidiasis without documented candidemia [4,5,8,9,10,14,42,43,45].

In candidemia, intermittent release of the microorganism into the bloodstream and the influence of sample volume may additionally reduce blood culture sensitivity. In neonates, where obtained blood volumes are usually smaller, this limitation becomes even more relevant [9,26,27,29,43].

The relatively slow growth of some fungi also prolongs the time to definitive identification and availability of antifungal susceptibility profiles, which may delay targeted therapeutic adjustments. In practical terms, an early negative test does not exclude IFI when significant clinical probability persists [4,7,11,74].

#### 3.2.2. Emergence of Non-Classical Fungi, Rare Pathogens, and Species with Clinically Relevant Antifungal Resistance

The contemporary epidemiology of IFI has evolved beyond *Candida albicans* and *Aspergillus fumigatus*. Widespread use of antifungal prophylaxis, improved survival among patients with profound immunosuppression, and hospital selective pressure have favored the relative increase in non-*albicans* yeasts, less common molds, emerging pathogens, and species with reduced or unpredictable antifungal susceptibility [23,25,47,48,60,75].

Among the agents of greatest clinical relevance are *Trichosporon* spp., *Saprochaete* spp., *Fusarium* spp., *Scedosporium* spp., *Lomentospora prolificans*, and other emerging opportunistic fungi. These microorganisms may not be detected by conventional biomarkers, may exhibit nonspecific morphology, or may be identified late through standard phenotypic methods [23,48,49,50,69].

This problem also includes yeasts with specific therapeutic and epidemiologic implications, such as *Candidozyma auris*, traditionally known as *Candida auris*; *Nakaseomyces glabratus*, traditionally known as *Candida glabrata*; and *Pichia kudriavzevii*, traditionally known as *Candida krusei*, in which species-level identification and antifungal susceptibility may directly modify standard clinical management [9,23,24,43,51,76].

The usual clinical consequence is incomplete or incorrect diagnosis, followed by initially inactive or insufficient treatment. Therefore, the capacity for species-level or species complex-level identification acquires increasing relevance in healthcare centers managing high-risk patients, especially when breakthrough infection, therapeutic failure, prior antifungal exposure, or suspected antifungal resistance is present [23,24,25,69].

#### 3.2.3. Fungal Coinfections

The simultaneous presence of more than one fungal pathogen, or a concomitant fungal infection with bacteria or viruses, adds substantial diagnostic complexity. This scenario is observed more frequently in critically ill patients, HSCT recipients, and patients with profound immunosuppression [9,22,42,46,64].

Clinically relevant examples include coinfection with *Aspergillus* and Mucorales, IC concomitant with persistent bacteremia, mixed pulmonary infections in the ICU, and coinfections associated with severe viral lung injury. In these cases, identification of one microorganism does not necessarily exclude the simultaneous presence of another infectious process [19,20,46].

The principal risk consists of prematurely closing the diagnostic process after an initial positive finding. From a clinical perspective, microbiological anchoring bias should be avoided when patient evolution is not concordant with the expected response to the initiated treatment. Lack of response, radiologic progression, or persistent sepsis should trigger diagnostic reassessment considering coinfection, an uncovered pathogen, antifungal resistance, or an alternative diagnosis [7,8,11,52,62,65,77].

### 3.3. Healthcare System-Related Factors

A substantial proportion of diagnostic failure in IFI depends not exclusively on the host or the pathogen, but on how the healthcare system attempting to recognize the infection is organized. Actual availability of diagnostic tests, turnaround times, interdisciplinary coordination, the existence of standardized diagnostic pathways, and institutional culture regarding antifungal use directly influence the timeliness and quality of diagnosis [7,8,11,31,32,37,69].

Even in hospitals with advanced resources, operational failures may neutralize the benefit of high-performance technologies. Conversely, centers with structural limitations may improve outcomes through efficient workflows, pragmatic algorithms, and close collaboration between the laboratory and clinical teams. Therefore, healthcare system determinants represent particularly relevant targets for continuous improvement interventions and AFSP-Dx [17,31,32,37].

In practice, the healthcare system may amplify or reduce diagnostic failure depending on how it organizes three critical processes: timely access to diagnostic testing, clinical–laboratory integration, and structured reassessment of empiric treatment. When these processes are not clearly defined, clinical suspicion may fail to translate into useful samples, results may be interpreted out of context, and antifungal therapy may be continued based on inertia rather than accumulated evidence.

#### 3.3.1. Limitations in Access to Diagnostic Testing

Unequal availability of biomarkers, rapid diagnostic tests, PCR, and other molecular diagnostic tools remains a key determinant of diagnostic failure, particularly in regions with limited resources or heterogeneous microbiological infrastructure [31,32,66,69].

When diagnostic testing is constrained by out-of-pocket costs, limited reimbursement, or local availability, tests should be selected according to their expected diagnostic and therapeutic yield. Prioritization should consider the host population, clinical syndrome, suspected pathogen, pretest probability, available specimens, local epidemiology, and the likelihood that the result will alter immediate management [22,31,32].

Depending on the clinical scenario, high-yield approaches may include adequately collected blood cultures for suspected candidemia; CrAg testing in patients at risk for cryptococcosis; targeted antigen testing for compatible endemic mycoses; and respiratory sampling combined with fungal biomarkers or molecular methods in selected patients with suspected invasive pulmonary mold disease. Imaging and tissue sampling should be prioritized when they may identify a focal process, provide etiologic confirmation, or guide source control [6,22,45,69].

Even in highly technological centers, the problem persists when tests are ordered too late, used outside the appropriate clinical context, or applied without clear institutional diagnostic algorithms. In these scenarios, the principal barrier is not the absence of technology, but rather its suboptimal implementation [6,7,31,32].

This limitation becomes particularly important in pathogens requiring specific testing or advanced identification, such as *C. auris*, emerging molds, Mucorales, cryptococcosis, or endemic mycoses. In these cases, lack of timely access to reliable identification, specific antigens, molecular testing, histopathology, or antifungal susceptibility testing may transform an otherwise correct clinical suspicion into an incomplete or delayed diagnosis [6,12,19,23,25,69,70,76,78,79,80,81,82].

#### 3.3.2. Clinical-Laboratory Fragmentation

Lack of structured communication among clinicians, microbiologists, mycologists, infectious diseases specialists, radiologists, and pathologists contributes substantially to interpretation errors and diagnostic delays [4,17,31,61].

Common examples include microbiological results reported without adequate clinical contextualization, histopathologic findings not integrated into the clinical scenario, or absence of bidirectional feedback when discordance exists between clinical evolution and laboratory findings. In IFI, where a single test rarely confirms or excludes the diagnosis definitively, this fragmentation may translate into incorrect or delayed therapeutic decisions [22,31,61].

Clinical-laboratory integration is particularly critical when findings may represent colonization, contamination, invasive infection, or an uncovered pathogen. A respiratory *Aspergillus* isolate in the ICU, a blood culture with unidentified yeasts, a biopsy showing hyphae suggestive of Mucorales, or an isolated positive biomarker all require integrated interpretation. Without this contextual reading, the risk of overdiagnosis, underdiagnosis, or treatment directed at the wrong pathogen increases.

#### 3.3.3. Excessive Reliance on Empiric Antifungal Therapy

Diagnostic uncertainty frequently favors prolonged empiric antifungal therapy, particularly in critically ill or severely immunosuppressed patients. Although this strategy may be appropriate in selected scenarios, its indiscriminate use has important consequences [18,32,42,43].

These include reduced subsequent diagnostic performance due to prior antifungal exposure, the risk of breakthrough infections caused by uncovered pathogens, and increased selective pressure associated with the emergence of antifungal resistance [23,24,52,53,54,67].

The consequences of diagnostic failure are not limited to inappropriate antifungal exposure. When an IFI is not promptly confirmed or reasonably excluded, broad-spectrum antibacterial therapy may also be continued unnecessarily, increasing drug-related toxicity, healthcare costs, and selective pressure contributing to antimicrobial resistance. Diagnostic stewardship for IFIs should therefore be integrated into the broader antimicrobial stewardship framework to optimize both antifungal and antibacterial use [31,32,37].

From a healthcare quality perspective, the goal should not be to replace diagnosis with empiric therapy, but rather to shorten diagnostic uncertainty through rapid diagnostic strategies, early reassessment, and formal AFSPs [31,32,37].

Reliance on empiric therapy becomes particularly problematic when no formal reassessment point exists at 48–72 h. In the absence of structured reevaluation, antifungal therapy may be continued because of the initial severity of illness, fear of discontinuation, or lack of diagnostic synthesis, even when the cumulative probability of an IFI decreases. Therefore, the system should require documented decisions regarding continuation, adjustment, de-escalation, or discontinuation of antifungal therapy, based on clinical evolution, sample quality, available results, and updated pretest probability.

### 3.4. Determinants of Diagnostic Failure According to Population

Although the general principles of diagnostic failure in IFIs are shared, their clinical expression varies substantially across different populations. The same diagnostic delay may arise from different causes in an ICU, a hematology unit, or a neonatology unit, including syndromic nonspecificity, profound immunosuppression, technical sampling limitations, or prior antifungal exposure, among others. Therefore, interpreting diagnostic failure without considering the population context may lead to incomplete or ineffective corrective strategies [22,31,32].

From a practical perspective, population-based analysis allows adaptation of diagnostic algorithms, definition of more realistic quality indicators, and prioritization of tests according to their expected performance. It also facilitates understanding why a diagnostic tool useful in immunocompetent adults may have very different value in critically ill patients, HSCT recipients, or premature neonates [9,12,13,14,22,26,27,28,29,45,46].

#### 3.4.1. Diagnostic Determinants in the Intensive Care Unit

In the ICU, diagnostic failure usually arises from the combination of a nonspecific clinical presentation, coexistence of multiple potential causes of sepsis, and the limited sensitivity of several conventional microbiological tools. Persistent fever, hemodynamic deterioration, or progressive respiratory failure are often initially attributed to bacterial, inflammatory, or invasive device-related etiologies [4,9,11,12,18,43,45].

This is compounded by the low sensitivity of blood cultures for certain forms of IC and the difficulty of interpreting biomarkers in critically ill patients with complex clinical conditions. Prior exposure to broad-spectrum antibiotics and, in some cases, empiric antifungal therapy may also alter diagnostic performance and delay etiologic confirmation [4,11,18,45,74].

In this setting, the problem is not only delayed recognition of an IFI, but also differentiation between colonization and invasive infection, particularly in the presence of respiratory isolation of *Aspergillus* or *Candida* colonization. Therefore, ICU diagnostic algorithms should incorporate dynamic pretest probability, criteria adapted to the critically ill patient, contextual interpretation of biomarkers, and early reassessment in order to avoid both underdiagnosis and overtreatment [19,20,21,22,33,46].

#### 3.4.2. Diagnostic Determinants in Hematology and HSCT

In patients with hematologic malignancies and HSCT, diagnostic failure is associated with profound immunosuppression, prior antifungal prophylaxis, and atypical clinical presentations. Azole exposure may reduce the sensitivity of cultures and biomarkers, while also favoring breakthrough infections caused by uncovered molds or emerging pathogens [5,19,23,52,53,67].

In this context, the need for preemptive diagnostic strategies becomes particularly relevant. Serial integration of imaging, biomarkers, and molecular testing allows timely initiation of treatment without necessarily waiting for definitive microbiological confirmation, although it requires a high degree of clinical coordination and interpretive expertise [13,22,55,68,72].

Moreover, in hematology/HSCT, diagnostic failure often takes the form of incomplete diagnosis: recognition that a probable IFI exists, but without timely identification of whether the causative agent is *Aspergillus*, Mucorales, *Fusarium*, other rare molds, emerging yeasts, or pathogens with clinically relevant antifungal resistance. This distinction has direct therapeutic implications, particularly in cases of azole nonresponse, breakthrough infection, or suspected antifungal resistance [19,23,24,25,53,54,60,75,77].

#### 3.4.3. Diagnostic Determinants in Neonatology

In neonatology, particularly in premature neonates and VLBW infants, diagnostic failure is favored by low initial clinical suspicion and by the similarity between IC and late-onset bacterial sepsis. Clinical signs are often subtle and nonspecific, making early suspicion difficult [26,27,28,29,30,44,64].

Furthermore, several diagnostic tools used in adults have limited validation in this population, while the small blood volumes available reduce blood culture yield. Blood culture volume should therefore be optimized according to patient weight and institutional laboratory protocols, because insufficient volume may further decrease diagnostic sensitivity. As a result, decision-making frequently depends on indirect clinical criteria, accumulated risk factors, and the clinical course during empiric therapy [26,27,28,64].

In neonates, diagnostic evaluation should early consider the role of the CVC, the possibility of urinary tract or CNS involvement, and the need to search for deep-seated foci even when blood cultures are negative or have low diagnostic yield. This approach is particularly relevant in VLBW and ELBW neonates with persistent sepsis without a clear bacterial explanation [26,27,28,29,44,64].

Population-specific diagnostic approaches are summarized in Table 2.

## 4. High-Risk Clinical Scenarios and Patterns of Diagnostic Failure in IFIs

Diagnostic failure in IFIs does not occur uniformly, but rather adopts specific patterns according to the clinical scenario, conditioned by the host profile, local epidemiology, the intensity of immunosuppression, and the available diagnostic strategies [4,7,8,22,31].

In practice, the determinants of diagnostic delay or error differ substantially among critically ill patients, HSCT recipients, individuals with hematologic malignancies, neonates, and other vulnerable populations. Whereas low initial clinical suspicion predominates in some settings, in others the central problem is prior antifungal exposure, difficulty obtaining deep samples, interpretation of colonization versus infection, or the emergence of less common fungal pathogens [5,18,23,28].

Identifying these patterns is essential for implementing targeted interventions, selecting appropriate diagnostic algorithms, and reducing the clinical impact of delayed or incorrect diagnosis. For this reason, analysis by clinical scenario provides a more useful framework than a uniform approach applied indiscriminately to all at-risk populations [17,31,32,37].

This approach also allows connection of the patient’s baseline risk with specific diagnostic decisions: when to intensify microbiological investigation, when to use advanced imaging or biomarkers, when to prioritize invasive sampling, and when to initiate or reevaluate antifungal therapy. In this way, scenario-based analysis not only describes where diagnostic failure occurs, but also guides more precise clinical and AFS interventions.

### 4.1. Intensive Care Unit

ICUs represent one of the most complex scenarios for the diagnosis of an IFI. In these patients, critical illness, multiple invasive devices, prolonged antibiotic exposure, acquired immune dysfunction, and a high frequency of nonspecific inflammatory syndromes converge. In this setting, IFIs are often confused with persistent bacterial sepsis, progressive organ failure, or complications inherent to critical hospitalization, favoring diagnostic delays and prolonged empiric therapy [4,11,18,22,26,43,83,90,91].

Furthermore, several traditional diagnostic tools exhibit particular limitations in the ICU: blood cultures with suboptimal sensitivity, biomarkers affected by multiple clinical variables, and radiologic findings that are less specific than in other populations. Therefore, diagnostic failure in this setting reflects both the biology of the infection and the complexity of the critically ill host [4,5,11,21,46,63].

### 4.1.1. Predominant IFIs in the ICU

In the ICU, the most relevant IFIs include candidemia and other forms of IC, ICU-associated pulmonary aspergillosis, and various emerging IFIs described in the context of severe COVID-19 or severe influenza [4,9,18,22,43].

These infections commonly coexist with bacterial sepsis, multiorgan failure, prolonged mechanical ventilation, and intensive use of broad-spectrum antibiotics, making early recognition difficult and reducing the specificity of the usual clinical signs [4,18,22].

It should also be considered that some IFIs in critically ill patients do not initially manifest as microbiologically evident syndromes. IC may present with initially negative blood cultures, whereas ICU-associated aspergillosis may present with poorly sensitive serum biomarkers and respiratory findings that are difficult to distinguish from colonization, viral lung injury, or concomitant bacterial pneumonia [4,13,19,20,21,46].

### 4.1.2. Patterns of Diagnostic Failure in the ICU

In clinical practice, diagnostic failure in the ICU often follows repetitive patterns that allow identification of opportunities for institutional improvement. Recognizing these trajectories is essential for designing syndrome-based algorithms, defining suspicion triggers, and establishing formal early reassessment points, particularly when the clinical course cannot be explained by bacterial etiologies or noninfectious causes [18,31,32].

In this setting, guidelines and reviews on mycologic diagnosis in pulmonary and critical care scenarios reinforce the need to select and interpret cultures, biomarkers, respiratory samples, imaging, and molecular methods according to the clinical syndrome, pretest probability, and risk of colonization [6,40,92].

1. Delayed diagnosis due to nonspecific clinical manifestations*:* Persistent fever, progressive respiratory deterioration, or hemodynamic instability are often initially attributed to multidrug-resistant bacteria, uncontrolled infectious foci, or noninfectious causes, delaying suspicion of an IFI [4,8,9,11,18].
2. Excessive reliance on blood cultures*:* The limited performance of blood cultures for detecting candidemia in early stages, as well as their limited value for invasive molds such as *Aspergillus*, favors initial false-negative results and delays in the initiation of targeted therapy [4,5,12,74].
3. Underdiagnosis of ICU-associated aspergillosis: Before the adoption of clinical definitions specific for critically ill non-neutropenic patients, many cases of pulmonary aspergillosis in the ICU were not recognized as true IFIs or were classified as simple respiratory colonization [13,14,20,21,41,42,46].
4. Empiric overtreatment due to diagnostic uncertainty: The opposite extreme is also frequent. In the presence of persistent sepsis, *Candida* colonization, or unexplained clinical deterioration, empiric antifungal therapy may be initiated and subsequently prolonged without documentation of an IFI. This pattern reflects persistent diagnostic uncertainty and absence of formal reassessment points, with risk of drug toxicity, unnecessary costs, and pharmacological selective pressure [18,24,31,32].

### 4.1.3. Clinical Consequences in the ICU

In the ICU, diagnostic failure is associated with delayed initiation of appropriate antifungal therapy, increased attributable mortality, prolonged hospital stay, and sustained empiric use of broad-spectrum antifungal agents [11,18,43,45,74,83].

This clinical pattern reinforces the need for early diagnostic strategies, contextualized interpretation of biomarkers, and serial microbiological reassessment in critically ill patients whose clinical course is not explained by bacterial etiologies. From an AFSP-Dx perspective, the goal is not to empirically treat all patients with critical deterioration, but rather to rapidly identify who requires immediate antifungal coverage, who needs diagnostic intensification, and in whom it is safe to de-escalate or discontinue antifungal therapy after structured reassessment.

### 4.2. Hematologic Patients and HSCT Recipients

Patients with hematologic malignancies and HSCT recipients constitute one of the populations at highest risk for IFIs and, at the same time, one of the most diagnostically challenging scenarios. Prolonged neutropenia, mucosal damage, profound cellular immunosuppression, and frequent exposure to antifungal prophylaxis modify both the epidemiology and the clinical presentation of these infections. As a result, diagnostic failure often manifests not as complete absence of suspicion, but rather as incomplete diagnoses, delayed confirmation, or insufficient interpretation of complex findings [5,11,19,23,45,72].

In this population, even brief diagnostic delays may have disproportionate consequences, given that tissue progression may be rapid and immune recovery uncertain. Therefore, diagnostic strategies must be dynamic, multimodal, and closely linked to clinical evolution, particularly in the presence of active antifungal prophylaxis, progressive radiologic findings, or lack of response to the initial antifungal agent [13,19,22,25,55,68,72].

In this population, guidelines and reviews specific to hematologic patients and HSCT recipients emphasize the need to consider active antifungal prophylaxis, breakthrough infection, the risk of infection caused by molds not covered by prophylaxis, antifungal resistance, and the feasibility of invasive sampling within individualized diagnostic strategies [68,93,94].

#### 4.2.1. Predominant IFIs in Hematologic Patients and HSCT Recipients

In patients with hematologic malignancies and HSCT recipients, predominant IFIs include IA, mucormycosis, and IFIs caused by less common fungi such as *Fusarium* spp., *Saprochaete* spp., *Trichosporon* spp., *Scedosporium* spp., *L. prolificans*, and other emerging opportunistic molds [5,19,23,25,48,49,50,60,75].

Despite advances in antifungal prophylaxis, diagnosis, and treatment, mortality remains high, particularly in disseminated infections, refractory disease, breakthrough infection, mucormycosis, or disease caused by pathogens with reduced or unpredictable antifungal susceptibility, especially when profound immunosuppression persists [19,20,23,25,72,78].

#### 4.2.2. Patterns of Diagnostic Failure in Hematology/HSCT

In clinical practice, diagnostic failure in hematology/HSCT tends to concentrate around four recurrent patterns: modification of test performance due to prior antifungal prophylaxis, insufficient detection of non-classical or emerging fungal pathogens, difficulty obtaining tissue confirmation, and absence of diagnostic reassessment in the setting of therapeutic nonresponse. These patterns are particularly relevant because they may lead to treatments that are initially active against the wrong pathogen or to prolongation of presumptive diagnoses without sufficient etiologic confirmation [7,13,23,72,84].

In this population, specific guidelines and reviews reinforce the need to interpret biomarkers, imaging, respiratory or tissue samples, etiologic identification, and antifungal susceptibility within the context of active antifungal prophylaxis, profound immunosuppression, breakthrough infection, and feasibility of invasive procedures [68,84,93,94,95].

1. Masking by antifungal prophylaxis: Azole prophylaxis modifies the clinical and microbiological presentation of IFIs, may reduce the sensitivity of cultures and certain biomarkers, and thereby delay diagnostic confirmation or generate falsely reassuring results [13,55,72].
2. Incomplete diagnosis of emerging or uncovered pathogens: Diagnostic algorithms focused primarily on *Aspergillus* may overlook mucormycosis or other fungi not susceptible to azoles, particularly when tissue samples, advanced mycological identification, or antifungal susceptibility testing are unavailable. This problem also includes rare molds, emerging yeasts, breakthrough infection, and pathogens with clinically relevant antifungal resistance, such as azole-resistant *A. fumigatus* or *C. auris* when the clinical syndrome corresponds to candidemia or invasive yeast infection [10,11,19,23,24,25,59,60,65,70,75,96,97].
3. Delay in histopathologic confirmation: Difficulty performing invasive procedures in thrombocytopenic patients or in those with respiratory compromise or clinical instability contributes to prolonged presumptive diagnoses and therapeutic decisions based on indirect evidence [5,6,11,72].
4. Absence of diagnostic reassessment in the setting of therapeutic nonresponse*:* Clinical or radiologic progression despite apparently appropriate antifungal therapy should be interpreted as a diagnostic warning signal. In this context, lack of response may reflect unrecognized mucormycosis, azole resistance, breakthrough infection, fungal coinfection, or a pathogen not covered by the initial regimen. Early reassessment prevents prolongation of inactive therapies and allows prioritization of tissue sampling, species- or species complex-level identification, antifungal susceptibility testing, and timely antifungal class switching [10,13,19,24,55,70,72,84,97].

#### 4.2.3. Clinical Consequences in Hematologic Patients and HSCT Recipients

In hematologic patients, diagnostic failure is associated with high mortality—frequently exceeding 50% in patients with invasive mucormycosis—prolonged use of initially ineffective antifungal agents, and delays in critical measures such as source-control surgery or timely switching of antifungal class [9,19,20,23,25].

These observations underscore the need for multimodal diagnostic strategies, early reassessment in the setting of initial nonresponse, timely access to histopathology, species- or species complex-level identification, and antifungal susceptibility testing when clinically indicated.

### 4.3. Neonatal Population

The neonatal population represents a unique scenario within diagnostic failure in IFIs, as it combines high biological vulnerability with specific diagnostic limitations. VLBW and ELBW neonates exhibit immunologic immaturity, fragile cutaneous-mucosal barriers, intensive exposure to invasive devices, and frequent courses of prolonged antibiotic therapy. In these patients, IC may progress rapidly while maintaining a subtle clinical presentation that is indistinguishable from other common neonatal conditions [26,27,28,30,44,64,85,98].

Unlike other populations, in neonatology diagnostic failure usually depends not only on the absence of sophisticated technologies, but also on difficulty in early suspicion of the infectious process, obtaining adequate samples, and interpreting findings within a highly dynamic physiology. Therefore, diagnostic delays may have disproportionate consequences for survival and neurodevelopment, particularly when initiation of appropriate antifungal therapy, evaluation of deep-seated foci, or intervention on the CVC is delayed [14,26,27,28,85,98].

#### 4.3.1. Predominant IFIs in Neonates

In the neonatal population, candidemia and other forms of IC continue to be the most frequent IFIs, particularly in VLBW and ELBW neonates, who concentrate the greatest burden of predisposing factors, such as the presence of a CVC, parenteral nutrition (PN), prolonged antibiotic therapy, and prolonged hospitalization [26,27,28,30,44,64,85].

Although less frequent, other IFIs may occur in critically ill neonates or those with complex hospital exposures. However, from the perspective of diagnostic failure, IC remains the priority model because it combines high clinical burden, nonspecific presentation, relatively low blood culture yield, and the need for early decisions regarding treatment, catheter management, and investigation of dissemination [26,27,28,98].

#### 4.3.2. Patterns of Diagnostic Failure in Neonates

In neonatal clinical practice, diagnostic failure often follows repetitive patterns related to low initial suspicion, technical limitations in sampling, and limited validation of complementary diagnostic tests. These patterns are relevant because a negative blood culture or a nonspecific clinical presentation does not exclude invasive disease in a high-risk neonate with persistent deterioration [27,29,64,98].

In this population, recent reviews emphasize that diagnostic interpretation should consider low clinical specificity, limited blood culture performance due to small sample volumes, incomplete validation of biomarkers or molecular methods, and the need for systematic CVC evaluation and targeted investigation of deep or uncontrolled foci, including the CNS, according to the clinical context [30,44,64,85,98].

1. Clinical overlap with late-onset bacterial sepsis: The clinical presentation is often indistinguishable from late-onset bacterial sepsis, with apnea, feeding intolerance, thermal instability, lethargy, or nonspecific hemodynamic deterioration. This clinical overlap frequently delays suspicion of candidemia and initiation of appropriate antifungal therapy [26,27,29,64].
2. Low sensitivity of blood cultures: The small blood volumes available for sample collection substantially limit blood culture yield, increasing the risk of falsely negative results or delayed microbiological diagnosis [6,27,98].
3. Lack of biomarker validation: 1,3-β-D-glucan (BDG), PCR, and other diagnostic methods are not fully validated for routine use in neonates because of variable cutoff values, limited standardization, and lower operational availability, restricting their widespread clinical implementation [27,45,98].
4. Incomplete evaluation of deep-seated foci and CNS involvement: An additional pattern of diagnostic failure occurs when neonatal candidemia is approached only as a bloodstream infection (BSI), without systematic investigation of deep-seated foci. In high-risk neonates, evaluation for urinary, ocular, cardiac, or CNS involvement should be considered according to the clinical presentation and institutional protocols, given that disseminated disease may persist even when blood cultures are negative or become negative during targeted therapy [27,59,74,85].

#### 4.3.3. Clinical Consequences in Neonates

In neonates, diagnostic failure is associated with higher mortality, increased risk of neurologic and neurodevelopmental complications, prolonged hospital stays, and greater utilization of critical care resources [26,27,64,99].

These observations support the need to maintain a low threshold for diagnostic suspicion and adopt early strategies in high-risk neonates with persistent sepsis without a clear bacterial explanation. In practice, this implies optimizing blood culture volume whenever possible, timely intervention on the CVC, evaluation of deep-seated foci, and documentation of criteria for continuation or discontinuation of empiric antifungal therapy.

### 4.4. Cross-Population Comparison of Patterns of Diagnostic Failure

Comparative analysis among the ICU, hematology/HSCT, and neonatal populations demonstrates that diagnostic failure adopts different expressions according to the host, local epidemiology, intensity of immunosuppression, and available diagnostic tools [14,22,26,31].

In the ICU, the predominant issues are clinical nonspecificity, coexistence with bacterial sepsis, complex interpretation of indirect tests, and the risk of empiric overtreatment due to diagnostic uncertainty. In hematology and HSCT, the main challenges include prior antifungal prophylaxis, the emergence of breakthrough infections, incomplete diagnosis of uncovered or emerging pathogens, and the need for preemptive strategies. In neonatology, prominent issues include low initial clinical suspicion, limitations of microbiological sampling, limited validation of several complementary diagnostic tests, and the need to evaluate deep-seated foci, including the CNS [18,27,41,55,85].

These differences reinforce that there is no single diagnostic algorithm universally applicable to all at-risk populations. On the contrary, diagnostic models must be adapted to the clinical context, the patient’s baseline risk, the expected rate of progression of the infectious process, the performance of available diagnostic tests, and institutional epidemiology [6,31].

Rather than comparing populations to establish a hierarchy of risk, this approach allows identification of the predominant type of failure in each setting: diagnostic delay and empiric antifungal overtreatment in the ICU, incomplete diagnosis in hematology/HSCT, and underdiagnosis due to sampling limitations in neonatology. This cross-population perspective facilitates selection of more precise interventions and avoids applying uniform solutions to distinct clinical problems [31,32].

### 4.5. Implications for Clinical Practice and the AFSP

Recognizing the specific patterns of diagnostic failure in each setting allows adjustment of diagnostic algorithms, prioritization of tests according to pretest probability, optimization of sampling timing, and reduction in unnecessary empiric antifungal use [17,31,32].

It also facilitates earlier therapeutic reassessment, improves the timeliness of targeted treatment, and supports antifungal de-escalation decisions when the probability of an IFI decreases during clinical evolution [18,31,32].

This approach is consistent with the objectives of AFSPs and with international recommendations aimed at improving clinical outcomes while limiting toxicity, associated costs, and selective pressure [13,31,32,37].

Operationally, an AFSP-Dx should translate these differences into population-specific pathways, with auditable indicators and formal reassessment points. In this way, the AFSP ceases to be merely a strategy for controlling antifungal consumption and becomes an institutional mechanism for reducing diagnostic delays, improving therapeutic precision, and balancing both the risk of undertreatment and the risk of unnecessary antifungal exposure.

The corresponding operational indicators are presented in Section 7 and Table 3.

## 5. Main Forms of Diagnostic Failure According to Type of IFI

Diagnostic failure in IFIs also adopts particular manifestations according to the pathogen involved, determined by differences in fungal burden, tissue tropism, rate of progression, host immune response, and the performance of available diagnostic tools [4,5,22,31].

In practical terms, some microorganisms are associated primarily with delayed clinical suspicion, whereas others pose greater difficulties in microbiological confirmation, species identification, or biomarker interpretation. These variations explain why strategies useful for a specific IFI may be insufficient, or even inappropriate, in other fungal entities [5,31,32].

Analyzing diagnostic failure according to the type of infection allows better understanding of the real limitations of each clinical algorithm and supports more precise decision-making in highly complex scenarios. The most relevant patterns for IFIs with the greatest clinical impact are summarized below, with emphasis on IC, IA, mucormycosis, and emerging pathogens [19,20,23,25,59,60,75,78].

From an AFS perspective, this etiologic approach is particularly useful because it avoids applying the same diagnostic logic to infections with very different biology. For example, a negative blood culture has different implications in IC, aspergillosis, or mucormycosis; similarly, biomarkers useful for septate molds may be poorly informative for Mucorales or certain rare or emerging fungi. Therefore, diagnostic interpretation should begin with the clinical syndrome, but be adjusted early according to the probable pathogen, the host, and the response to initial antifungal therapy [13,19,20,31,32,59,60,75,84].

### 5.1. Candidemia and Invasive Candidiasis

Candidemia and other forms of IC continue to be the most frequent IFIs in numerous hospitals and represent a classic model of diagnostic failure. Although consolidated global guidelines and greater availability of complementary diagnostic tools exist, a substantial proportion of cases continue to be recognized late or after clinical deterioration has already been established. This is due, in part, to the fact that the disease often presents with signs indistinguishable from bacterial sepsis and that conventional microbiological tests continue to have important limitations [4,16,18,43,59,74,91].

From an operational perspective, IC illustrates how the combination of low clinical suspicion, limited blood culture performance, prior antimicrobial exposure, and incomplete species-level identification may translate into therapeutic delays with direct impact on survival. This problem becomes particularly relevant in relation to non-*albicans* species, recently reclassified species, and emerging pathogens such as *C. auris*, in which incorrect or delayed identification may postpone therapeutic, epidemiologic, and transmission-control measures. Therefore, IC remains one of the principal targets of early AFSP-Dx, together with source control, reliable identification, antifungal susceptibility testing, and structured therapeutic reassessment [9,18,24,31,32,43,44,59,74,91,103].

Recent global candidiasis guidelines reinforce this approach by underscoring the importance of timely diagnosis, source control, correct etiologic identification, and therapeutic optimization according to the species and antifungal susceptibility profile when clinically relevant [59,103].

#### 5.1.1. Predominant Diagnostic Failure Pattern in Candidemia and Invasive Candidiasis

In candidemia and IC, the predominant pattern of diagnostic failure corresponds to delayed diagnosis and initial underdiagnosis, particularly in patients with persistent sepsis without a clear source or with poorly specific clinical manifestations [4,18,22,43,74,91].

#### 5.1.2. Main Mechanisms of Diagnostic Failure in Candidemia and Invasive Candidiasis

Among the most frequent mechanisms are the limited performance of blood cultures, particularly in early stages or in deep candidiasis without documented fungemia; intermittent release of the microorganism into the bloodstream; insufficient sample volumes; and prior antifungal exposure before culture collection [4,5,74,91].

Added to this is the difficulty in differentiating colonization from true infection in certain clinical settings, as well as the tendency to initially attribute the clinical syndrome to more prevalent bacterial etiologies [4,18,33,90].

An additional mechanism of growing relevance is incomplete or delayed identification of species with specific therapeutic and epidemiologic implications. This includes non-*albicans* yeasts, species with reduced antifungal susceptibility or intrinsic antifungal resistance, and emerging pathogens such as *C. auris*, whose incorrect identification may delay both appropriate antifungal selection and measures for prevention and control of nosocomial outbreaks [23,24,51,70,79,80,104].

#### 5.1.3. Population-Specific Impact of Candidemia and Invasive Candidiasis

In the ICU, delays of ≥24–48 h in the initiation of appropriate antifungal therapy have been associated with increased attributable mortality, prolonged hospital stay, and worse clinical outcomes [18,21,24,41,74,90].

In hematology and HSCT, episodes of breakthrough candidemia caused by less susceptible or resistant species, such as *N. glabratus*, *P. kudriavzevii*, and *C. auris*, may occur, particularly during or after azole prophylaxis or due to prior echinocandin exposure. In these scenarios, accurate species- or complex-level identification and antifungal susceptibility testing acquire direct clinical importance. This point is especially relevant for non-*albicans* yeasts, emerging species, or cryptic species within clinically relevant complexes, in which incomplete phenotypic identification may obscure susceptibility differences or delay recognition of antifungal resistance. As a consequence, the initially selected antifungal therapy may prove suboptimal or inadequate [23,24,70,75,105].

In neonates, the presentation is often indistinguishable from late-onset bacterial sepsis, and diagnostic sensitivity is further reduced by the small blood volumes obtained for blood cultures [26,27,28,30,64,85].

#### 5.1.4. Practical Diagnostic Message for Candidemia and Invasive Candidiasis

A negative blood culture does not exclude candidemia/IC in high-risk patients. When significant clinical suspicion persists, complementary diagnostic strategies, serial reassessment, and timely initiation of antifungal therapy according to the clinical context should be considered [4,32,43,59,74].

Furthermore, in the setting of candidemia caused by non-*albicans* species, suspected *C. auris*, breakthrough infection, or isolates belonging to species complexes with variable susceptibility, reliable species- or complex-level identification and antifungal susceptibility testing should be considered essential components of diagnosis, not ancillary steps. In these cases, diagnostic failure may consist not only of delayed recognition of candidemia, but also delayed identification of the correct pathogen or underestimation of a clinically relevant antifungal susceptibility profile [23,24,70,75,105].

### 5.2. Invasive Aspergillosis

IA represents one of the greatest diagnostic challenges among IFIs because it frequently occurs without detectable fungemia, depends on indirect evidence, and shares clinical manifestations with multiple nonfungal pulmonary diseases. In contrast to candidemia, where bloodstream isolation may guide diagnosis, confirmation of aspergillosis usually requires integration of imaging, biomarkers, respiratory microbiology, and clinical context. This explains why diagnostic failure often adopts erroneous or incomplete forms, rather than simple absence of clinical suspicion [5,13,19,46,51,53,54,70,75,84,105,106].

Moreover, contemporary epidemiology has expanded the spectrum of affected patients to include critically ill individuals without classic neutropenia, requiring reconsideration of previous diagnostic paradigms. In this setting, early clinical suspicion remains as important as technological availability, especially when serum biomarkers are negative or when pulmonary disease is initially interpreted as viral, bacterial, or inflammatory injury [13,18,21,46,56,84].

Recent guidelines and reviews on aspergillosis reinforce that diagnosis should rely on integration of imaging, biomarkers, respiratory microbiology, molecular methods, and clinical context, with particular caution in critically ill non-neutropenic patients and in scenarios of prior azole exposure or suspected antifungal resistance [19,63,84,96,107,108].

#### 5.2.1. Predominant Diagnostic Failure Pattern in Invasive Aspergillosis

In IA, the predominant pattern of diagnostic failure corresponds to erroneous or incomplete diagnosis, derived from the difficulty of confirming infection through conventional methods and the frequent clinical overlap with other pulmonary pathologies [5,10,19,46].

#### 5.2.2. Main Mechanisms of Diagnostic Failure in Invasive Aspergillosis

Among the most relevant mechanisms are the usual absence of detectable fungemia, the variable sensitivity of galactomannan (GM) according to host type and prior antifungal exposure, nonspecific radiologic findings in early stages, and the difficulty of obtaining deep respiratory samples or pulmonary tissue in clinically unstable patients [5,11,13,109,110].

In non-neutropenic patients, especially critically ill patients, the diagnostic utility of certain serum biomarkers may be lower than in classical hematologic populations, increasing the risk of false-negative results and diagnostic delay [13,46,63,106,107].

An additional mechanism of incomplete diagnosis occurs when *Aspergillus* spp. is identified without completing species- or species complex-level identification, or without evaluating antifungal susceptibility in clinically relevant scenarios. This aspect acquires particular importance in the setting of therapeutic failure, prior azole exposure, radiologic progression, or epidemiologic contexts suggestive of azole-resistant *A. fumigatus*, in which assuming a susceptibility profile may delay appropriate therapeutic adjustment and prolong ineffective treatment [10,19,84,96,97].

Furthermore, the existence of cryptic species within *Aspergillus* complexes may contribute to incomplete diagnoses when isolates are reported only at the genus or complex level. In these scenarios, particularly in the setting of therapeutic failure, prior azole exposure, radiologic progression, or epidemiologic suspicion of antifungal resistance, this limitation reinforces the need for more precise identification and antifungal susceptibility testing whenever feasible [108,111].

#### 5.2.3. Population-Specific Impact of Invasive Aspergillosis

In critically ill patients, ICU-associated aspergillosis has historically been underdiagnosed, particularly in patients with severe influenza or severe COVID-19, in whom the respiratory syndrome is often initially attributed to viral infection and underlying pulmonary injury [19,21,46,84,106].

In hematology and HSCT, azole prophylaxis may delay diagnostic confirmation and contribute to clinical and radiologic overlap with bacterial or viral pneumonias, treatment-associated pulmonary toxicity, or other noninfectious causes. Furthermore, in patients with prior azole exposure or progressive disease despite treatment, the possibility of azole resistance should be incorporated into diagnostic-therapeutic reasoning, since the problem is not always simply recognizing aspergillosis, but determining whether the initial antifungal agent is truly active [46,55,63,68,84,96,106].

In neonates, IA is uncommon, but when it occurs, it is usually associated with ELBW neonates, immunocompromised status, or invasive procedures, and is associated with high mortality [14,28,64].

#### 5.2.4. Practical Diagnostic Message for Invasive Aspergillosis

In critically ill patients, aspergillosis should be considered even in the absence of neutropenia or with negative serum GM. Clinical suspicion, imaging, and integrated respiratory microbiology remain essential to avoid diagnostic delays [13,21,41,46].

When *Aspergillus* spp. is documented in a patient with probable invasive disease, clinical failure, prior azole exposure, or suggestive local epidemiology, species- or species complex-level identification and antifungal susceptibility assessment should be considered part of the operational diagnosis. In these scenarios, diagnostic failure consists not only of delayed recognition of aspergillosis, but also of failure to timely detect antifungal resistance or an etiology with direct therapeutic implications.

### 5.3. Mucormycosis

Mucormycosis represents one of the most aggressive forms of IFI and probably the clearest example of how diagnostic failure may translate into rapid loss of therapeutic opportunity. Unlike other IFIs, in which greater support from biomarkers or indirect microbiologic evidence exists, diagnosis in mucormycosis frequently depends on early clinical suspicion, procurement of viable tissue, and urgent histopathological and microbiological integration. When these steps are delayed, angioinvasive and necrotic progression may become fulminant [20,112,113].

Therefore, in this entity the problem is usually not only identifying the pathogen, but rapidly recognizing a compatible clinical syndrome before tissue damage becomes irreversible. This characteristic makes mucormycosis a particularly time-sensitive infection from a diagnostic perspective, in which any delay in obtaining tissue for diagnosis, initiating surgical management, or switching to an antifungal agent active against Mucorales may have a decisive impact on prognosis [20,114,115].

Specific guidelines and reviews on mucormycosis agree that diagnosis should prioritize early clinical suspicion, urgent tissue procurement, histopathological and microbiological integration, evaluation of anatomic extent, and complementary use of PCR when available, without interpreting negative GM or BDG results as evidence of exclusion [20,112,114,116].

#### 5.3.1. Predominant Diagnostic Failure Pattern in Mucormycosis

In mucormycosis, the predominant pattern of diagnostic failure is delayed diagnosis, with cases diagnosed in advanced stages and even *postmortem* in historical series [20,114,115,116].

#### 5.3.2. Main Mechanisms of Diagnostic Failure in Mucormycosis

Several factors contribute to this diagnostic delay. Although Mucorales may grow rapidly, their recovery in culture is inconsistent and depends on sample quality, microbiologic processing, and tissue viability. In addition, conventional serum biomarkers, such as GM and BDG, are usually negative, limiting indirect diagnostic support [20,112,115,117].

Added to this is the clinical and radiologic similarity to IA or other necrotizing infections, particularly in early pulmonary or rhinosinusal disease, which may lead to initially inappropriate treatment [20,115,116].

A relevant component of diagnostic failure occurs when suspicion of mucormycosis is delayed because of initial anchoring on IA, especially if the patient is receiving treatment with voriconazole or another azole without adequate activity against Mucorales. In these scenarios, the absence of biomarker positivity should not be interpreted as reassuring evidence, and clinical or radiologic progression should trigger an urgent diagnostic pathway that includes tissue procurement, imaging studies to define the extent of disease, and early surgical evaluation [20,25,114,116].

#### 5.3.3. Population-Specific Impact of Mucormycosis

In hematology and HSCT, mucormycosis continues to carry high mortality—frequently exceeding 50%—particularly when persistent neutropenia is present or when surgical debridement and initiation of appropriate antifungal therapy are delayed [20,93,113].

In the ICU, an increase in cases associated with uncontrolled diabetes mellitus, corticosteroid exposure, MV, and severe COVID-19 has been observed in certain epidemiologic contexts [20,93].

In neonates, the entity is extremely uncommon, but when it occurs, it is usually diagnosed late and is associated with significant adverse clinical outcomes [26,64].

#### 5.3.4. Practical Diagnostic Message for Mucormycosis

Presumed aspergillosis that fails to respond clinically to voriconazole, especially in the presence of compatible risk factors or radiologic progression, should raise suspicion for mucormycosis until proven otherwise. In these cases, urgent tissue procurement and therapeutic reassessment are priorities, since delays in diagnostic procedures in mucormycosis have been associated with increased mortality [20,93,114].

In mucormycosis, a negative GM or BDG result does not exclude invasive disease. Diagnostic decision-making should rely on clinical probability, radiologic pattern, the presence of necrosis or angioinvasion, tissue sampling, and the early need for surgical source control when indicated.

### 5.4. Rare, Emerging, Endemic Fungi and Other Pathogens with Frequently Overlooked Diagnosis

IFIs caused by rare, emerging, endemic fungi or other opportunistic pathogens with frequently overlooked diagnosis constitute a growing challenge for modern healthcare systems. Although they are less frequent than IC, aspergillosis, or mucormycosis in many hospital settings, their clinical relevance has increased because of improved survival of severely immunosuppressed patients, widespread use of antifungal prophylaxis, hospital selective pressure, global patient mobility, and persistence of diagnostic gaps in endemic regions. In this group, diagnostic failure is commonly expressed as low initial suspicion, delayed identification, incorrect microorganism classification, or clinical underestimation of an isolate considered unusual [25,53,57,60,69,70,75,96].

The need to improve identification, surveillance, and prioritization of these pathogens is also consistent with the WHO fungal priority pathogens list, which highlights the clinical and public health impact of resistant, emerging, or insufficiently diagnosed fungal species [47,48].

Unlike other IFIs with more consolidated diagnostic algorithms, these infections require high clinical suspicion, epidemiologic correlation, and access to advanced identification methods. In addition, several species exhibit intrinsic antifungal resistance, unpredictable susceptibility profiles, or specific diagnostic requirements, so diagnostic delay may rapidly translate into exposure to an inactive antifungal agent, suboptimal treatment, or treatment directed against the wrong pathogen [7,8,11,60,69,75].

Recent guidelines on rare fungi, emerging yeasts, cryptococcosis, and endemic mycoses emphasize that diagnosis should combine clinical suspicion, epidemiologic exposure, reliable species- or species complex-level identification, specific testing—such as cryptococcal antigen (CrAg) or antigens/serologies for endemic mycoses—and antifungal susceptibility testing when clinically relevant, especially in the setting of pathogens with intrinsic antifungal resistance or unpredictable susceptibility [45,47,57,58,60,75,118,119,120].

#### 5.4.1. Predominant Diagnostic Failure Pattern in Rare, Emerging, and Endemic Fungal Infections

In IFIs caused by rare, emerging, endemic, or opportunistic fungi not always considered in the initial diagnostic approach, the predominant pattern of diagnostic failure corresponds to incomplete or incorrect diagnosis. This is usually related to low clinical suspicion, difficulties in microbiologic identification, absence of specific testing in many laboratories, and lack of integration among epidemiology, geographic exposure, immune status, and clinical findings [23,45,57,65,69,108].

In this setting, the problem is not only “failing to detect a fungus,” but also failing to recognize in a timely manner that the probable agent may require a different test, microbiologic interpretation, or therapeutic strategy from those used for *Candida*, *Aspergillus*, or Mucorales.

#### 5.4.2. Relevant Agents

Among the opportunistic agents of greatest clinical relevance are *Trichosporon* spp., *Saprochaete* spp., *Fusarium* spp., *Scedosporium* spp., *L. prolificans*, *Rhodotorula* spp., *Magnusiomyces* spp., and other less frequent fungi, whose importance has increased in severely immunosuppressed patients, HSCT recipients, patients with prolonged neutropenia, and individuals receiving care in tertiary-care centers [48,49,50,60,75].

In addition to these rare molds and emerging yeasts, *Cryptococcus* spp. should be considered among the opportunistic pathogens whose diagnosis may be overlooked when the clinical syndrome is not integrated with the host immune status. Although it is not strictly an emerging fungus, cryptococcosis may present as subacute meningitis, pneumonia, fungemia, or disseminated disease, especially in patients with cellular immunosuppression, transplantation, corticosteroid or biologic use, or unrecognized HIV infection. In these scenarios, failure to request early CrAg testing in serum or cerebrospinal fluid (CSF) may significantly delay diagnosis [12,45,58,118,121].

In endemic regions or in patients with compatible epidemiologic histories, systemic mycoses such as histoplasmosis, coccidioidomycosis, blastomycosis, paracoccidioidomycosis, and talaromycosis should also be considered. These infections are not necessarily rare within their areas of circulation, but they may behave as overlooked diagnoses when geographic exposure is not recognized, when they are mistaken for tuberculosis, malignancy, bacterial pneumonia, or other inflammatory diseases, or when specific mycological testing is unavailable [57,119,120,122].

#### 5.4.3. Main Mechanisms of Diagnostic Failure in Rare, Emerging, and Endemic Fungal Infections

The most common mechanisms include delayed or incorrect identification using conventional phenotypic methods, limited availability of advanced techniques such as MALDI-TOF or fungal DNA sequencing, and delay in the clinical interpretation of unusual fungal isolates [7,11,69,73,123].

A related problem is the presence of cryptic species within clinically relevant yeast and mold complexes. These isolates may be indistinguishable using conventional phenotypic methods, exhibit different antifungal susceptibility profiles, and remain underestimated when MALDI-TOF MS with updated databases, sequencing, or validated molecular methods are unavailable [60,75,105,108,121,124,125].

Added to these factors are intrinsic antifungal resistance or multidrug resistance to commonly used antifungal agents in several of these species, as well as episodes of breakthrough fungemia associated with prior exposure to echinocandins or azoles [23,52,53,65].

In cryptococcosis, diagnostic failure is commonly related to low clinical suspicion, failure to request early CrAg testing in serum or CSF, initial interpretation as bacterial meningitis, tuberculosis, malignancy, or inflammatory disease, and lack of systematic evaluation for disseminated disease, especially outside the classical context of advanced HIV infection [12,58,118,121].

In the case of endemic fungi, the main mechanism is usually not antifungal resistance, but rather low epidemiologic suspicion, clinical overlap with tuberculosis, bacterial disease, malignancy, or inflammatory disease, and the absence or limited local availability of rapid testing. Therefore, a history of residence, travel, occupational exposure, environmental contact, or origin from endemic areas should be considered part of the diagnostic process, especially in immunosuppressed patients [45,57,122].

#### 5.4.4. Population-Specific Impact of Rare, Emerging, and Endemic Fungal Infections

In hematology, these infections are associated with high mortality, rapid progression, and frequent therapeutic failure, especially when profound neutropenia persists or when the initial treatment does not cover the actual fungal pathogen [25,60,126].

In the ICU, persistent fungemias associated with intravascular devices, prolonged antibiotic exposure, or prior antifungal exposure may occur, further complicating microbiologic control [18,75,83].

In neonates, cases are sporadic, but lethality may be high when recognition and treatment are delayed [64].

In patients with cellular immunosuppression, transplantation, or undiagnosed HIV infection, cryptococcosis may present with nonspecific neurologic or pulmonary symptoms, and delay in requesting CrAg testing or evaluating the CSF may be associated with delayed diagnosis of meningitis or disseminated disease [58,118].

In endemic regions, or in patients originating from them, systemic mycoses should be incorporated early into the differential diagnosis in the setting of prolonged fever, pulmonary involvement, disseminated disease, cutaneous lesions, hepatosplenomegaly, lymphadenopathy, or unexplained deterioration, particularly in immunocompromised patients [57].

#### 5.4.5. Practical Diagnostic Message for Rare, Emerging, and Endemic Fungal Infections

Species-level identification—and ideally antifungal susceptibility testing when available—is critical in the management of these infections. Diagnostic delay favors initially ineffective therapies and substantially worsens prognosis [7,11,45,69].

In patients with compatible epidemiologic factors, the differential diagnosis should be expanded beyond *Candida*, *Aspergillus*, and Mucorales. In these scenarios, failure to recognize a rare, emerging, endemic, or opportunistic fungus may represent a form of incomplete diagnosis, even when it has been correctly recognized that the patient has an IFI [7,82].

### 5.5. Comparative Synthesis of Patterns of Diagnostic Failure

The comparative analysis according to IFI type demonstrates that patterns of diagnostic failure are not uniform and depend, to a large extent, on pathogen biology, tissue tropism, speed of clinical progression, and the performance of available diagnostic tools [5,8,38,83,127,128].

In candidemia and IC, delayed diagnosis predominates, driven by the limited sensitivity of blood cultures, initial clinical nonspecificity, and, in non-*albicans* species or emerging yeasts such as *C. auris*, by the need for reliable identification and availability of antifungal susceptibility testing. In IA, erroneous or incomplete diagnoses are more frequent, deriving from variable biomarkers, absence of detectable fungemia, nonspecific radiologic findings, and possible azole resistance in *A. fumigatus* in selected scenarios. In mucormycosis, severe diagnostic delay predominates, favored by usually negative serum biomarkers, the frequent need for tissue confirmation, and the urgency of surgical source control. In rare, emerging, endemic, or opportunistic fungi with frequently overlooked diagnosis, the main problem is usually low clinical suspicion, incorrect or delayed identification, absence of specific diagnostic tests, antifungal resistance, or lack of epidemiological integration, as occurs with endemic mycoses or cryptococcosis outside classical clinical settings [10,12,23,51,52,57,58,70,97].

These findings confirm that diagnostic failure depends not only on technological availability, but also on the adequacy of the diagnostic algorithm used for each pathogen and clinical scenario [18,32,41].

### 5.6. Cross-Cutting Clinical Implications

Although patterns of diagnostic failure differ according to the microorganism involved, their clinical consequences are consistently adverse: increased mortality, therapeutic delay, prolonged hospital stays, potentially avoidable drug toxicity, and inefficient use of resources [4,17,24,31,59,78].

Exclusive reliance on conventional diagnostic methods, particularly when they are used in isolation or late, contributes to perpetuating diagnostic delay. This is especially evident when definitive microbiologic confirmation is awaited in scenarios where culture performance is limited, biomarkers may be negative or poorly informative, clinical progression is rapid, or the pathogen requires specific etiologic identification methods [4,7,38,73,128,129].

Therefore, clinical–laboratory integration, syndromic assessment, epidemiologic context, and the rational combination of conventional and nonconventional methods are essential to reduce diagnostic errors. In high-risk patients, decision-making should rely on dynamic pretest probability and serial reassessment rather than on a single negative diagnostic test [17,31,32].

## 6. Limitations of Current Diagnostic Tools and How They Contribute to Diagnostic Failure in IFI

The performance of diagnostic methods in IFIs is conditioned by multiple biologic and clinical variables, including low fungal burden, heterogeneous tissue distribution, prior antifungal exposure, and pretest probability determined by the population being evaluated and the healthcare setting [4,5,8,17,33].

Consequently, diagnostic failure rarely depends on a single deficient test. More often, it reflects the interaction between intrinsic limitations of available methods—imperfect sensitivity, variable specificity, prolonged turnaround times, or incomplete validation—and errors in clinical indication, inappropriate sample selection, suboptimal timing of sample collection, and decontextualized interpretation of results [4,7,32,37,82,128,130,131].

These limitations have been recognized in recent guidelines and reviews on mycologic diagnosis, which emphasize the need to interpret cultures, biomarkers, molecular methods, and imaging according to the clinical syndrome, the population being evaluated, and the healthcare setting, especially in pulmonary and critical care scenarios [6,38,40,128].

This approach is particularly relevant in IFIs, where a negative test does not always exclude invasive disease and a positive result may require careful clinical correlation to differentiate colonization, contamination, or true invasive infection [4,5,7]. Furthermore, a negative blood culture, a nonreactive biomarker, or nonspecific imaging findings may have very different implications in IC, aspergillosis, mucormycosis, cryptococcosis, endemic mycoses, or rare/emerging fungi. This variability reinforces the need to integrate each result with pretest probability, local epidemiology, prior antifungal exposure, and serial clinical evolution [11,12,31,32,38,45,58,61,84,128].

Therefore, understanding the real limitations of each diagnostic tool is essential to reduce delays, avoid overtreatment, and design more effective algorithms according to patient type, clinical syndrome, and the suspected fungal pathogen.

### 6.1. Blood Culture and Conventional Culture

Blood culture and conventional culture remain historical pillars of mycologic diagnosis because they allow direct microbiologic confirmation, identification of the etiologic agent, and, in many cases, performance of antifungal susceptibility testing. However, their actual clinical utility is conditioned by limitations in sensitivity, turnaround time, and variable performance according to the infectious syndrome. Consequently, exclusive reliance on these tools may favor diagnostic delays, generate a false sense of reassurance in the setting of negative results, and perpetuate prolonged empiric therapeutic decisions [4,8,31,38,61,129].

From a contemporary perspective, culture retains essential value, but it should be integrated into multimodal diagnostic strategies rather than assumed to be the sole operational standard. This consideration is particularly relevant in deep IFIs, localized infections without fungemia, mold disease, endemic mycoses, cryptococcosis with atypical presentations, and patients previously exposed to antifungal agents. In these scenarios, performance is conditioned not only by the microorganism involved, but also by timing of collection, sample type and volume, microbiologic processing, and clinical pretest probability [4,5,6,43,57,58,59,78,84].

*Strengths*. Blood culture and conventional culture provide direct microbiologic evidence. When positive, they allow confirmation of the presence of a viable microorganism, guide etiologic identification, and, in many cases, enable antifungal susceptibility testing. This information is particularly important in candidemia, infections caused by non-*albicans* species, emerging yeasts, rare molds, and episodes of breakthrough infection, where identification of the etiologic agent may directly modify therapeutic selection [16,23,24,43,51,52,91,131].

Furthermore, culture remains indispensable for documenting species with therapeutic or epidemiologic implications, such as *C. auris*, *N. glabratus*, *P. kudriavzevii*, *Fusarium* spp., *Scedosporium* spp., *L. prolificans*, and other emerging opportunistic pathogens [48,51,60,70,75,132].

*Main limitations*. Despite their value, these tools present relevant limitations that contribute to diagnostic failure in IFIs. In candidemia, their diagnostic performance is imperfect, and in deep IC it is even more variable; therefore, a negative result does not exclude infection, especially when there has been prior antifungal exposure, intermittent fungemia, or delayed sample collection [11,12,59,131].

In IA, the performance of blood culture is virtually nil, given that fungemia is uncommon and infection usually remains localized in pulmonary tissue or other organs [19,78,84].

In addition, microbiologic growth may be slow and conventional phenotypic identification may be incomplete, especially for rare, emerging, reclassified, or difficult-to-identify species. This may delay initiation of targeted therapy, detection of antifungal resistance, and optimization of the therapeutic regimen [25,60,75].

*Impact according to population*. In the ICU, candidemia with initially negative blood cultures is not uncommon in patients with concomitant bacterial sepsis, favoring therapeutic delay and persistence of diagnostic uncertainty [9,18,36,43]. In addition, interpretation of respiratory cultures must be cautious: *Candida* spp. isolates usually represent colonization, whereas *Aspergillus* spp. in the airway requires structured evaluation to differentiate colonization from invasive disease in critically ill patients [33,46,56,63,81,83,133,134].

In hematology and HSCT, prior antifungal prophylaxis may further reduce culture performance and favor infections caused by non-classical or emerging pathogens that are less easily recognized using conventional diagnostic algorithms [13,52,54,55,67,72,84]. In this population, culture is also essential for detecting breakthrough infections, rare molds, and pathogens with reduced or unpredictable antifungal susceptibility [6,52,67].

In neonates, the low blood volumes obtained for blood culture significantly reduce diagnostic sensitivity, favoring underdiagnosis and delay in initiation of appropriate antifungal therapy [26,27,30,64,85]. Therefore, a negative blood culture should not be considered sufficient to exclude IC in a high-risk neonate with persistent sepsis.

In patients with suspected cryptococcosis or endemic mycoses, culture may confirm the diagnosis, but its turnaround time and the need for appropriate samples may limit its usefulness for early decision-making. Therefore, when the clinical and epidemiologic context suggests these infections, culture should be complemented with specific tests such as CrAg, fungal antigens, histopathology, or molecular methods according to local availability [12,38,57,58].

*Practical mitigation—AFSP-oriented approach.* Early collection of blood cultures before initiation of antifungal therapy should be prioritized, using adequate volumes according to age, weight, and clinical conditions whenever feasible.

In addition to selecting the appropriate test, diagnostic performance depends on preanalytical factors that are frequently underestimated: sample volume, collection site, timing relative to initiation of antifungal therapy, transport, timely processing, and prior communication with microbiology or pathology when special cultures, histopathology, or molecular methods are required. Optimizing this workflow may reduce losses in diagnostic performance before the sample reaches the laboratory [6,38,40,130].

When clinical suspicion persists despite negative cultures, the diagnostic strategy should be expanded through biomarkers, targeted imaging, and procurement of samples from the suspected focus, avoiding exclusive reliance on culture [31,32,37].

From an AFSP-Dx perspective, culture should be optimized, not abandoned. This implies improving the timeliness and quality of sampling, ensuring rapid communication of critical results, promoting species- or species complex-level identification when therapeutically relevant, and linking microbiologic results with explicit decisions regarding continuation, adjustment, de-escalation, or discontinuation of antifungal therapy.

### 6.2. Histopathology and Biopsy

Histopathology and biopsy occupy a central role in the diagnosis of IFIs, particularly when indirect methods are ambiguous or when rapid confirmation of tissue invasion is required. Unlike biomarkers or cultures, which may reflect colonization, contamination, or limited sensitivity, tissue allows documentation of true infectious disease in its anatomic context. This capability is decisive in entities such as mucormycosis, localized aspergillosis, endemic mycoses with tissue involvement, and focal lesions of uncertain etiology [4,5,12,20,43,57,59,78].

However, their potential value does not always translate into timely use. The need for invasive procedures, logistical barriers, and delays in decision-making often turn biopsy into a late diagnostic test, requested when disease has already progressed. From the perspective of diagnostic failure, the problem is not only method availability, but also when, in whom, and through which processing workflow it is used [31,78,84].

*Strengths*. Histopathology and biopsy continue to be high-value diagnostic tools in IFIs because they allow demonstration of direct tissue invasion and, in many cases, differentiation of superficial colonization from true invasive infection. This capability is especially relevant when cultures are negative or biomarkers are inconclusive [6,12].

In addition, tissue evaluation may provide information regarding angioinvasion, necrosis, local extension, and inflammatory response, elements with relevant diagnostic and therapeutic implications. In mucormycosis, for example, demonstration of tissue invasion and necrosis may accelerate the indication for surgery and transition toward therapy active against Mucorales; in endemic mycoses, histopathology may guide diagnosis when rapid tests are unavailable or when presentation mimics tuberculosis, malignancy, or inflammatory disease [20,57,84,115].

Biopsy may also allow parallel processing for culture and molecular testing, increasing the probability of etiologic identification, especially in molds, Mucorales, rare or emerging fungi, endemic mycoses, or focal lesions with a broad differential diagnosis [19,25,60,69,75,115].

*Main limitations.* A major limitation is the need for invasive procedures. In hematologic patients with thrombocytopenia, unstable critically ill patients, and neonates, tissue procurement may be impracticable or involve significant risks [11,64,72,112,116].

Fungal morphology is also not always fully discriminatory. Distinguishing between septate and pauciseptate hyphae may be difficult in suboptimal samples, necrotic tissue, or in the presence of artifacts, favoring incomplete diagnoses or erroneous interpretations [19,78].

Furthermore, performance closely depends on sample quality and depth, histologic processing, the special stains employed, and the experience of the pathologist and microbiology team [135,136]. Therefore, a suggestive histopathologic report should always be integrated with culture, mycologic identification, molecular testing when available, and the clinical-epidemiologic context, especially in the setting of emerging fungi, rare molds, Mucorales, tissue cryptococcosis, or endemic mycoses [48,49,57,58,69,123].

*Impact according to population.* In critically ill patients, tissue procurement may be limited by hemodynamic instability, MV, coagulopathy, or high procedural risk. In this context, many decisions must rely on indirect evidence, which may prolong presumptive diagnoses, favor empiric overtreatment, or delay confirmation of invasive disease [130].

In hematology and HSCT, histopathology is particularly important for differentiating aspergillosis, mucormycosis, other opportunistic molds, neoplastic disease, pulmonary toxicity, or viral infection. However, thrombocytopenia, profound neutropenia, and respiratory compromise may delay or prevent invasive procedures, favoring incomplete diagnoses and initially inappropriate antifungal therapy [5,6,19,72].

In neonates, biopsy is rarely an initial diagnostic tool because of its invasiveness and limitations related to body size, clinical stability, and procedural safety. In this population, evaluation of deep-seated foci more commonly relies on blood cultures, targeted cultures, imaging, CNS evaluation, and clinical-microbiological follow-up [14,64,98].

In endemic mycoses or cryptococcosis with tissue involvement, histopathology may be decisive when the presentation mimics tuberculosis, malignancy, or inflammatory disease. However, if the sample is not also sent for culture or complementary testing, the diagnosis may remain incomplete and fail to allow definitive identification of the etiologic agent [6,57,58,118,119].

*Practical mitigation—AFSP-oriented approach.* It is recommended to establish institutional “dual-sample” pathways, sending tissue simultaneously for histopathology and, whenever possible, an additional sterile portion for culture and/or molecular testing.

In the setting of suspected mucormycosis, negative serum biomarkers should not be relied upon. Priority should be given to early activation of the tissue-based diagnostic pathway, evaluation of anatomic extent, and coordination of surgical source control when indicated [20,114,115]. From an AFSP-Dx perspective, this requires predefined clinical criteria and prior coordination among the treating team, radiology/interventional services, surgery, pathology, and microbiology to avoid sample loss, inadequate processing, or reports not integrated into therapeutic management.

### 6.3. Identification of the Etiologic Agent

Identification of the etiologic agent constitutes a central component of modern mycologic diagnosis and should not be considered merely a taxonomic step. In IFIs, recognizing that a fungal infection exists may be insufficient if the implicated genus, species, or complex is not precisely defined, especially when the result modifies antifungal selection, epidemiologic control measures, or the need to broaden the diagnostic workup. This point is particularly relevant in candidemia caused by non-*albicans* species, suspected *C. auris*, breakthrough infections, emerging molds, mucormycosis, aspergillosis with therapeutic failure, and rare pathogens with unpredictable antifungal susceptibility [12,23,24,60,75,79,84,137].

From the perspective of diagnostic failure, incomplete characterization of the agent may perpetuate initially inactive treatments, delay switching antifungal class, omit transmission control measures, or maintain an erroneous interpretation of the clinical syndrome [124,125,131].

*Strengths.* Species- or species complex-level identification allows differentiation of pathogens with divergent therapeutic implications. In candidemia, for example, distinguishing among *C. albicans*, *N. glabratus*, *P. kudriavzevii*, or *C. auris* may modify antifungal selection, the need for susceptibility testing, and prevention and control measures. In mold infections, differentiating *Aspergillus*, Mucorales, *Fusarium*, *Scedosporium*, *L. prolificans*, or other emerging fungi may prevent inappropriate treatment and guide the need for tissue procurement, surgery, or switching antifungal class [20,48,49,51,70,78,115].

Modern platforms—including MALDI-TOF MS with updated databases, ITS/D1-D2 region sequencing, targeted PCR, and sequencing of tissue samples—may improve diagnostic accuracy when conventional phenotypic methods are insufficient. This point is particularly relevant in *C. auris*, whose phenotypic identification may be inaccurate and requires MALDI-TOF MS with updated databases, sequencing, or validated molecular methods [51,79,82,124,125,131].

*Main limitations.* Etiologic identification may be delayed because of slow growth, absence of a viable isolate, inadequate samples, negative cultures due to prior antifungal exposure, or limitations of the database used. In addition, not all platforms identify rare species, recently reclassified fungi, or emerging pathogens with equal accuracy. In some cases, the result may remain limited to genus or complex level, reducing its therapeutic utility [6].

This limitation is especially relevant in the setting of cryptic species within *Candida*, *Aspergillus*, *Fusarium*, *Scedosporium*, or other opportunistic fungal complexes. In these cases, an apparently sufficient identification may conceal clinically relevant differences in susceptibility, virulence, or therapeutic response, such that the diagnosis may remain incomplete even when the microorganism genus has been recognized [60,75,105,108,111].

Another relevant limitation is the clinical interpretation of isolates obtained from nonsterile sites. Identification of *Candida* spp. in respiratory samples, for example, usually represents colonization, whereas isolation of *Aspergillus* spp. from the airway of critically ill patients requires structured interpretation to differentiate colonization from invasive disease. Therefore, microorganism identification must always be integrated with sample type, the clinical syndrome, and pretest probability [33,41,43,46,133,138].

*Impact according to population*. In the ICU, etiologic identification may help differentiate colonization from invasive infection, recognize candidemia caused by species with lower antifungal susceptibility, and detect epidemiologically important pathogens such as *C. auris*. In this context, early communication among microbiology, infectious diseases specialists, and the treating team is essential to avoid both overtreatment and therapeutic delay [6,17,33].

In hematology and HSCT, species- or species complex-level identification acquires particular relevance in the setting of breakthrough infections, progressive disease during antifungal prophylaxis, suspected mucormycosis, rare molds, or azole resistance in *A. fumigatus*. In these patients, a diagnosis reporting only “mold” or “yeast” may be insufficient to guide appropriate active therapy [10,66,67,97,112].

In neonates, precise identification of yeasts in candidemia is important for adjusting treatment, evaluating antifungal susceptibility, and defining management decisions regarding the CVC and the search for deep-seated foci. However, the utility of advanced methods depends on local availability, sample volume, and the possibility of recovering a viable isolate [44,64].

*Practical mitigation—AFSP-oriented approach.* The AFSP-Dx should define which isolates require priority species- or species complex-level identification. This includes candidemia, non-*albicans* yeasts, suspected or confirmed *C. auris*, breakthrough infections, molds isolated from sterile sites or deep samples, refractory disease, prior antifungal exposure, and pathogens with epidemiologic implications [31,32,61,130].

In addition, a rapid communication pathway for “alert pathogens” should be established to ensure timely notification to the treating team, infectious diseases specialists, microbiology, and infection control, as appropriate. From an operational perspective, etiologic identification should translate into documented decisions: maintaining, adjusting, escalating, de-escalating, or discontinuing antifungal therapy; requesting antifungal susceptibility testing; broadening the extent-of-disease workup; or activating prevention and control measures.

### 6.4. Antifungal Susceptibility Testing

Antifungal susceptibility testing allows estimation of the in vitro activity of antifungal agents against the recovered fungal isolate and constitutes a key tool when the result may modify clinical management. In the context of IFIs, its utility is greatest in candidemia caused by non-*albicans* species, suspected *C. auris*, breakthrough infection, prior antifungal exposure, therapeutic failure, aspergillosis with possible azole resistance, meningeal or disseminated cryptococcosis, refractory mucormycosis, and rare or emerging fungi with unpredictable antifungal susceptibility [10,65,70,77,80,127,137].

Its value is not limited to confirming resistance. It also allows recognition of reduced susceptibility profiles, guidance of antifungal class changes, support for de-escalation when the isolate is susceptible, and development of institutional surveillance. From a diagnostic failure perspective, omitting antifungal susceptibility testing when it has clinical implications may convert an apparently correct microbiologic diagnosis into an incomplete diagnosis.

*Strengths.* Antifungal susceptibility testing helps select targeted therapy, detect acquired or intrinsic antifungal resistance, and anticipate the risk of therapeutic failure. In candidemia, it is particularly relevant for species with reduced or variable susceptibility, such as *N. glabratus*, *P. kudriavzevii*, or *C. auris*. In aspergillosis, it may be critical when there has been prior azole exposure, progression during antifungal therapy, or epidemiologic suspicion of azole-resistant *A. fumigatus*. In rare fungi, antifungal susceptibility testing may be one of the few available tools to guide treatment when clinical evidence is limited [23,70,97,125,127,132,137,139].

Reference methods, such as broth microdilution for yeasts and filamentous fungi, provide a standardized basis for interpreting minimum inhibitory concentrations and comparing results among laboratories. CLSI and EUCAST documents help harmonize methodology and interpretation of results, although for some emerging species, such as *C. auris*, tentative breakpoints or provisional criteria may be required according to public health recommendations [80,140,141,142,143,144].

*Main limitations.* Susceptibility testing is not always available in a timely manner and requires a viable isolate, sufficient growth, specialized infrastructure, and technical expertise. In addition, for several rare fungi, emerging molds, or species-antifungal combinations, clinical breakpoints may be nonexistent or limited, so interpretation depends on epidemiologic cutoff values, laboratory experience, clinical context, and therapeutic response [77,134,137,142,144].

Another important limitation is that in vitro susceptibility does not replace clinical evaluation. Treatment response also depends on the site of infection, antifungal penetration, fungal burden, source control, immunosuppression, surgery when indicated, and drug concentrations. Therefore, a “susceptible” result does not guarantee clinical success, and a result showing reduced susceptibility should be interpreted together with patient evolution [77,127,137].

When there are concerns regarding drug exposure, drug toxicity, drug interactions, or therapeutic failure, therapeutic drug monitoring (TDM) may complement interpretation of antifungal susceptibility and clinical response. However, its utility varies according to the antifungal agent and the strength of the available evidence, so it should be applied in a contextualized manner [100,145].

*Impact according to population.* In the ICU, antifungal susceptibility testing is especially useful in persistent candidemia, prior echinocandin exposure, suspected *C. auris*, or unfavorable evolution despite apparently adequate treatment. It may also support de-escalation decisions when the fungal isolate and clinical evolution allow it [43,125,137].

In hematology and HSCT, its impact is greater in breakthrough infections, emerging molds, *A. fumigatus* with possible azole resistance, non-*albicans* yeasts, and clinical failure during antifungal prophylaxis or prior antifungal therapy. In this population, antifungal susceptibility testing may prevent the prolongation of inactive regimens and justify early changes in antifungal class [65,111].

In neonates, antifungal susceptibility testing may be relevant in persistent candidemia, prior fluconazole exposure, infection caused by non-*albicans* species, or clinical failure. Nevertheless, its implementation depends on recovery of the fungal isolate, laboratory availability, and the need to interpret results within pediatric or neonatal protocols [77].

*Practical mitigation—AFSP-oriented approach.* AFSP-Dx should define institutional criteria for requesting antifungal susceptibility testing and avoid making it depend exclusively on individual decisions. At a minimum, it should be considered in candidemia, non-*albicans* species, suspected or confirmed *C. auris*, breakthrough infection, prior antifungal exposure, therapeutic failure, clinically relevant mold isolates, *A. fumigatus* with suspected azole resistance, and rare or emerging fungi [31,65,70,111].

This indication should also be extended to isolates belonging to species complexes or cryptic species with variable susceptibility profiles, especially when invasive disease, therapeutic failure, prior antifungal exposure, or breakthrough infection is present [53,67,111].

Results should be communicated in an actionable manner, ideally with joint clinical interpretation among microbiology, infectious diseases, and the treating team. In addition, AFSP-Dx should audit indicators such as the proportion of eligible episodes with antifungal susceptibility testing performed, time from isolation to result, time to therapeutic adjustment, and concordance among the susceptibility profile, antifungal received, and clinical outcome. Within this framework, antifungal susceptibility testing ceases to be an isolated laboratory datum and becomes a tool for therapeutic precision and institutional surveillance.

### 6.5. Biomarkers: GM, BDG, CrAg, and Antigen Testing for Endemic Mycoses

Fungal biomarkers and non-culture-based antigenic or serologic tests have substantially expanded diagnostic capacity in IFIs, especially in scenarios where culture is slow, negative, or difficult to obtain. Their principal value lies in anticipating suspicion, supporting preemptive strategies, and complementing conventional microbiologic evaluation. However, their utility closely depends on the population being evaluated, sample type, prior antifungal exposure, pretest probability, and the suspected pathogen [6,13,81,82,92,109,110,129,135,146].

From the perspective of diagnostic failure, the risk lies not only in lacking biomarkers, but also in using them as binary or universal tests. An isolated positive result may favor overtreatment if not clinically interpreted, whereas a negative result may generate false reassurance in infections where performance is limited, such as mucormycosis, aspergillosis in non-neutropenic patients, deep IC without documented fungemia, cryptococcosis, or endemic mycoses not considered within the differential diagnosis [4,15,38,45,71,128,129].

Moreover, not all biomarkers follow the same diagnostic logic. Whereas GM and BDG provide useful indirect information in certain aspergillosis or IC scenarios, other specific tests—such as CrAg or antigens for endemic mycoses—require interpretation according to the clinical syndrome, epidemiology, and host immune status. This distinction is relevant to avoid a strategy focused solely on GM/BDG from overlooking pathogens such as *Cryptococcus* spp., Mucorales, or endemic mycoses in patients with compatible risk factors [12,16,45,57,58,84].

#### 6.5.1. Galactomannan (GM)

GM is a biomarker directed primarily toward the diagnosis of IA. Its utility depends on the population being evaluated, the site of infection, the sample type, and prior exposure to antifungal agents active against molds. Therefore, its interpretation should not be uniform across hematology/HSCT, ICU, and neonatology, nor should it replace integration with imaging, respiratory microbiology, histopathology, or molecular testing when indicated [19,78,84].

*Strengths*. GM is one of the best-established biomarkers for IA and has demonstrated diagnostic utility in both serum and respiratory samples, although its performance varies according to the population being evaluated, prior antifungal exposure, and the type of sample analyzed [6,13,84,109,135].

Meta-analyses and systematic reviews support its diagnostic value and show that the cutoff values used substantially modify sensitivity and specificity. In particular, GM in bronchoalveolar lavage (BAL) has shown significant utility, although it requires bronchoscopy and interpretation integrated with clinical pretest probability [46,71,92,106,110].

*Main limitations.* In critically ill and non-neutropenic patients, the sensitivity of serum GM is usually lower than in classical hematologic populations, and difficulties persist in applying diagnostic criteria originally designed for hematology [21,41,46,56]. In addition, prior exposure to antifungal agents active against molds may reduce test performance and delay positivity [13,72,84].

GM also does not identify species, anatomic extent, or antifungal susceptibility. Therefore, a positive result does not replace the need for culture, mycologic identification, molecular testing, or evaluation of antifungal resistance when there is therapeutic failure, prior azole exposure, or suspicion of resistant *A. fumigatus* [78,84,96].

*Impact according to population.* In hematology and HSCT, GM retains particular utility within preemptive diagnostic strategies, especially when interpreted together with imaging, clinical evolution, and baseline risk. However, during anti-mold prophylaxis, a negative GM should not generate false reassurance if progressive radiologic lesions, persistent fever, or clinical deterioration are present [6,78,84,135].

In the ICU, serum GM may have low sensitivity, and GM in BAL usually provides greater value, provided it is interpreted within diagnostic algorithms adapted to critically ill patients, such as AspICU, BM-AspICU, or specific CAPA criteria when appropriate [21,46,56,83,130,147].

In neonates, the utility of GM is much less established and requires extreme caution, given the lack of robust validation, variability in cutoff values, and the need for strict clinical correlation [14,44,64].

*Practical mitigation—AFSP-oriented approach.* GM should be used as part of integrated diagnostic algorithms for aspergillosis, not as an isolated test. In hematologic patients, it should be interpreted serially and together with computed tomography (CT), baseline risk, and prior antifungal prophylaxis. In the ICU, priority should be given to integrating GM in BAL, respiratory microbiology, imaging, and clinical evolution to differentiate colonization from invasive disease [12,17,31,37].

From an AFSP-Dx perspective, a negative GM should not close the diagnostic evaluation when pretest probability is high, and a positive GM should not justify prolonged antifungal therapy without clinical correlation. Its result should trigger explicit decisions: intensify diagnostic evaluation, obtain BAL or tissue, initiate treatment, adjust coverage, investigate resistance, or de-escalate when the accumulated evidence does not support IFI [82,135,136].

#### 6.5.2. 1,3-β-D-Glucan

BDG is a broad-spectrum fungal biomarker that may support the diagnosis of some IFIs, especially IC and aspergillosis. However, its broad scope also constitutes a limitation: it does not allow pathogen identification, does not localize the infectious focus, and does not discriminate among colonization, active infection, environmental exposure, or clinical interferences. Therefore, its principal value is to modify diagnostic probability within an integrated algorithm, not to confirm or exclude an IFI in isolation [6,40,72,84,92,110].

*Strengths.* BDG may support the diagnosis of IC and aspergillosis when interpreted together with clinical criteria, imaging studies, and other microbiologic tests [4,7,15,38,43,81,146].

Meta-analyses in patients with hematologic malignancies support its possible role as a complementary test, although they emphasize that it should not be used in isolation or outside the appropriate clinical context [5,42,72,82,131,146]. Its main operational utility is to contribute to a diagnostic probability strategy, especially when cultures are negative or when the goal is to reduce uncertainty in high-risk patients.

*Main limitations.* BDG does not identify the implicated species, anatomic location, or antifungal susceptibility profile. In addition, false-positive or false-negative results may occur depending on concomitant exposures, blood products, certain therapies, heavy colonization, sample contamination, or prior antifungal therapy, which increases the risk of overtreatment or false diagnostic reassurance when it is used in isolation [4,9,11,82,110,130,146].

BDG has limited or no utility in several clinically relevant entities. In mucormycosis, conventional serum biomarkers are usually negative; in cryptococcosis, the diagnostic approach should rely on specific tests such as CrAg; and in endemic mycoses, epidemiologic suspicion and the clinical syndrome should guide the selection of specific antigens and serologic tests [12,20,45,58,84].

In neonates, evidence remains limited, with methodological variability and difficulties in establishing universally accepted cutoff values [14,44,98].

*Impact according to population.* In the ICU, BDG may help reduce diagnostic uncertainty in selected patients with suspected IC, but it may also favor overtreatment if interpreted as an isolated confirmatory test. Its value increases when combined with pretest probability, colonization status, clinical evolution, imaging studies, cultures, and reevaluation at 48–72 h [17,31,41,61].

In hematology and HSCT, it may complement diagnostic strategies, but it does not replace targeted mold evaluation, GM, CT, BAL, biopsy, or molecular testing when the main suspicion is aspergillosis, mucormycosis, or breakthrough infection [69,73,123,128,129].

In neonates, its use should be particularly cautious because of limited validation, variability in cutoff values, and neonatal physiology. In this population, BDG should not replace optimized blood cultures, catheter evaluation, the search for deep-seated foci, or serial clinical assessment [30,98].

*Practical mitigation—AFSP-oriented approach.* BDG should be incorporated as a complementary test within defined institutional algorithms, with clear criteria for indication, interpretation, and reevaluation. From an AFSP-Dx perspective, it should be interpreted as a probability modifier, not as a confirmatory test or as automatic justification for prolonging empiric antifungal therapy. Its utility lies in increasing or decreasing the cumulative probability of IC or aspergillosis, supporting a preemptive decision, justifying diagnostic intensification, or facilitating safe discontinuation of antifungals when the overall body of evidence does not support an IFI. In mucormycosis, cryptococcosis, and endemic mycoses, a negative or noncontributory BDG should not generate false reassurance [72,110,146].

#### 6.5.3. Cryptococcal Antigen and Specific Tests for Cryptococcosis

Cryptococcosis requires a diagnostic logic distinct from that of candidiasis or aspergillosis. In this entity, GM and BDG are not the central tests; early diagnosis depends on ordering CrAg in the appropriate clinical scenario. This point illustrates a frequent form of diagnostic failure: applying an algorithm centered on biomarkers for *Candida/Aspergillus* while omitting a specific test for a pathogen with subacute, neurologic, pulmonary, or disseminated presentation [12,58,82,118,121].

*Strengths.* In cryptococcosis, diagnosis should not rely on GM or BDG, but rather on specific CrAg testing in serum or CSF, such as lateral flow assays/devices (LFA/LFD), latex agglutination, or enzyme immunoassay. These tests facilitate early recognition of cryptococcal meningitis, pulmonary disease, or disseminated infection, particularly in patients with cellular immunosuppression, transplantation, corticosteroid exposure, or unrecognized HIV infection [12,45,121].

Its principal strength lies in substantially reducing diagnostic time when ordered in the appropriate clinical context. In patients with subacute meningitis, intracranial hypertension, nonspecific neurologic manifestations, nodular pneumonia, or disseminated disease, CrAg may guide early therapeutic decisions before culture confirms the diagnosis [45,121,129].

*Main limitations.* From the perspective of diagnostic failure, the most frequent problem is not the absolute absence of testing, but rather failure to order CrAg early when the clinical syndrome and host immune status justify it. Initial interpretation as bacterial meningitis, tuberculosis, malignancy, or inflammatory disease may delay diagnosis, particularly when cryptococcosis presents outside the classic context of advanced HIV infection [6,12,82].

CrAg also does not completely replace comprehensive clinical evaluation. In suspected cryptococcal meningitis, CSF evaluation, opening pressure measurement, culture, and assessment for disseminated disease remain necessary according to the clinical scenario [58].

*Impact according to population.* In patients with cellular immunosuppression, transplantation, corticosteroid exposure, biologic therapies, or unrecognized HIV infection, failure to request CrAg early may delay recognition of cryptococcosis, especially when it presents as subacute meningitis, pneumonia, or disseminated disease [58].

In hematology and HSCT, although cryptococcosis is not the predominant IFI, it should be considered in unexplained neurologic, pulmonary, or disseminated syndromes, especially when the clinical pattern does not fit aspergillosis, candidiasis, or mucormycosis. In the ICU, cryptococcosis may present as severe meningitis, respiratory failure, or sepsis of unclear origin. In these cases, failure to include CrAg within the differential diagnosis may perpetuate nondirected antibacterial or antifungal therapies. In neonates, cryptococcosis is exceptional and does not represent a routine diagnostic pathway. However, the general principle remains: biomarkers should be selected according to syndrome, epidemiology, and host, not as a universal panel [82,118,121].

*Practical mitigation—AFSP-oriented approach.* AFSP-Dx programs should incorporate clinical triggers for ordering CrAg in patients with subacute meningitis, compatible neurologic symptoms, unexplained pneumonia, disseminated disease, or relevant cellular immunosuppression. This pathway should be independent of GM/BDG, because a strategy focused only on biomarkers for *Candida/Aspergillus* may overlook cryptococcosis.

In patients with positive CrAg results, the program should facilitate clinical confirmation, CNS evaluation when appropriate, rapid communication of the result, and appropriate therapeutic selection according to current guidelines [12,58,121].

#### 6.5.4. Antigen Testing and Serology for Histoplasmosis and Other Endemic Mycoses

In endemic mycoses, diagnostic failure usually originates before testing: the diagnosis is not suspected. These infections may mimic tuberculosis, malignancy, bacterial pneumonia, sarcoidosis, or other inflammatory diseases, especially in endemic regions or in immunocompromised patients. Therefore, antigenic and serologic tests should be selected according to geographic exposure, the clinical syndrome, expected fungal burden, and host immune status [45,57,120,122].

*Strengths.* In histoplasmosis and other endemic mycoses, antigen detection in urine and serum may be especially useful in disseminated disease or in immunocompromised patients, whereas serology, culture, and histopathology provide value according to the clinical form, local availability, and the stage of disease evolution [69,119,120,122,129].

These tests allow expansion of the differential diagnosis beyond *Candida*, *Aspergillus*, and Mucorales, especially in patients from endemic areas, with compatible environmental exposure, or with pulmonary/disseminated disease of uncertain etiology [119,120].

*Main limitations.* In endemic mycoses, the principal limitation is usually not only analytic, but also clinical and epidemiologic: the diagnosis is not suspected. Overlap with tuberculosis, malignancy, bacterial pneumonia, sarcoidosis, or other inflammatory diseases may delay ordering of specific tests [57,120,122].

The availability of antigen testing, serology, specialized culture, histopathology, or molecular testing may vary substantially among regions and hospital institutions. In addition, the performance of each test depends on the clinical form, immune status, fungal burden, stage of disease evolution, and the species involved [119,120].

*Impact according to population.* In immunocompromised patients, especially those with cellular immunosuppression, transplantation, biologic therapies, or undiagnosed HIV, endemic mycoses may present as disseminated disease, prolonged fever, pulmonary involvement, hepatosplenomegaly, lymphadenopathy, cutaneous lesions, or systemic deterioration. Failure to consider them early may lead to delayed diagnosis or treatment directed toward tuberculosis, bacterial infections, or malignancies before considering a fungal etiology [119].

In hematology and HSCT, endemic mycoses may be confused with other IFIs or with progression of the underlying disease. In the ICU, they may present as respiratory failure, sepsis, or multiorgan failure. In neonates, they are much less frequent, but geographic exposure and the epidemiologic context remain determining factors if the clinical presentation suggests them [120].

*Practical mitigation—AFSP-oriented approach.* In patients from endemic areas or with compatible exposures, the diagnostic strategy should integrate epidemiology, the clinical syndrome, antigen testing, serology, histopathology, and culture, avoiding premature closure of the diagnosis as tuberculosis, malignancy, or bacterial infection [119,122].

AFSP-Dx should include clinical-epidemiologic triggers to activate specific testing for endemic mycoses—for example, Histoplasma antigen testing, serologies, or molecular tests according to availability—in patients with prolonged fever, unexplained pulmonary disease, disseminated involvement, or relevant immunosuppression [119].

### 6.6. Molecular Diagnostics and Rapid Identification/Resistance Testing

Molecular testing and rapid identification methods have acquired an increasing role in the diagnosis of IFIs, especially when conventional methods are slow, have low sensitivity, or do not allow timely identification of pathogens with relevant therapeutic implications. Their value is particularly evident in high-risk patients, breakthrough infections, prior antifungal exposure, suspected resistance, or scenarios in which pathogens requiring divergent therapeutic strategies, such as aspergillosis and mucormycosis, must be differentiated [6,38,69,73,86,97,117,123,129].

However, these tools do not eliminate the risk of diagnostic failure. Their implementation may be limited by lack of standardization, unequal availability, costs, insufficient local validation, and the risk of detecting DNA from nonviable or colonizing microorganisms. Therefore, they should be interpreted within integrated algorithms, together with clinical, histopathologic, microbiologic, and imaging data, and not as automatic substitutes for culture, histopathology, or clinical judgment [6,117,123,128,129].

Recent reviews on molecular diagnostics and nonconventional methods emphasize that these tools may reduce time to identification or support diagnosis in culture-negative cases, but they require local validation, contextual interpretation, and combination with conventional clinical, histopathologic, and microbiologic data [6,12,117,129].

#### 6.6.1. Species-Specific PCR

Species-specific or targeted PCR allows the detection of a specific pathogen when clinical suspicion is high and the result may rapidly modify therapeutic management. Relevant examples include PCR for *A. fumigatus*, PCR for Mucorales in tissue or selected respiratory samples, and PCR for *Pneumocystis jirovecii* in patients with compatible interstitial pneumonia and immunosuppression [38,86,123,129].

In mucormycosis, PCR may be especially useful as a complement to tissue diagnosis when culture is negative or microbiologic recovery is limited, although its interpretation should always be integrated with histopathology, imaging studies, clinical probability, and local availability [112,116,123].

*Strengths.* These tests may shorten diagnostic time, especially in fungal infections with low culture recovery or in patients previously exposed to antifungals. In aspergillosis, PCR may complement GM, culture, and imaging; in mucormycosis, it may support diagnosis when culture is negative; and in *P. jirovecii* pneumonia, it may contribute to confirming a diagnosis that is not always documented by conventional methods [6,69,78,82,84].

*Main limitations.* Its main limitation is that it detects only the pathogen for which it was designed. A negative result does not exclude another IFI nor replace syndromic evaluation. In addition, performance depends on sample type, fungal burden, the platform used, the positivity threshold, and local validation [7,11,129]. In the case of *P. jirovecii*, a positive result must also be interpreted together with the clinical syndrome, imaging findings, assay burden when available, and host immune status, in order to avoid confusing colonization or low burden with active disease [129,130].

*Impact according to population.* In hematology/HSCT, it may be useful in the presence of pulmonary lesions, prior antifungal prophylaxis, or suspected breakthrough infection. In the ICU, it may support the diagnosis of ICU-associated aspergillosis or *P. jirovecii* pneumonia when the clinical context justifies it. In neonates, its application requires greater caution, given that clinical validation is more limited and the available specific evidence remains scarce [7,94,107,130].

*Practical mitigation—AFSP-oriented approach.* AFSP-Dx should define for which syndromes, populations, and samples a targeted PCR is activated. Ordering it as a “universal panel” without a clear clinical question should be avoided. Its result should be linked to actionable decisions: initiate therapy, change drug class, obtain tissue, de-escalate, or investigate alternative diagnoses [7,31].

#### 6.6.2. Panfungal ITS/18S/28S PCR and Sanger/NGS Sequencing

Panfungal PCR amplifies conserved regions of fungal DNA—such as ITS, 18S, or 28S—and is combined with Sanger sequencing or NGS to identify fungi not recovered by culture. Its greatest utility is observed in tissue, biopsy material, or deep samples, especially when histopathology demonstrates fungal elements but culture is negative or was not properly submitted [101].

EORTC/MSGERC recommendations recognize the value of molecular identification in tissue containing fungal elements, although they caution that isolated amplification without histopathologic evidence is insufficient to prove invasive infection [22,101,123].

*Strengths.* It may identify unexpected, rare, or difficult-to-culture pathogens. It is particularly useful in molds, Mucorales, emerging fungi, and endemic mycoses when tissue is available. In addition, it may reduce incomplete diagnosis by providing genus- or species-level identification when histologic morphology is insufficient, and may help resolve cryptic species or species complexes when phenotypic identification does not distinguish taxa with therapeutic implications, especially in deep samples or tissue with demonstrated fungal elements [101,123].

*Main limitations.* It is vulnerable to contamination, inhibitors, DNA degradation, and interpretive difficulties. In formalin-fixed, paraffin-embedded tissue, performance depends on tissue quality, fixation time, fungal burden, and the presence of visible fungal elements. A negative result does not exclude IFI, and a positive result must be interpreted together with histopathology and the clinical context [6,101].

*Impact according to population.* In hematology/HSCT, it may be decisive when biopsy is obtained late or culture is negative because of prior antifungal prophylaxis. In the ICU, it may help in focal lesions or unexplained pulmonary disease if tissue is obtained. In neonates, its role is limited and depends on the feasibility of obtaining deep samples [23,69,73].

*Practical mitigation—AFSP-oriented approach.* It should preferably be activated in deep or tissue samples with strong suspicion of IFI, especially when culture is negative or unavailable. AFSP-Dx should ensure “dual-sample” pathways: tissue for histopathology and sterile tissue for culture/PCR whenever possible. It should also define how inconclusive results, potential contaminants, or discordant findings will be reported [18,94,130].

#### 6.6.3. Molecular Detection of Antifungal Resistance

Molecular detection of antifungal resistance seeks genetic markers associated with reduced antifungal susceptibility. A relevant example is the identification of cyp51A mutations in *A. fumigatus*, associated with azole resistance. Antifungal resistance markers in yeasts may also be considered, such as FKS mutations in species within the *N. glabratus* complex, when platforms and local validation allow it [10,96,97,139,148,149].

*Strengths.* It may accelerate suspicion of resistance and guide a therapeutic change before phenotypic susceptibility results are available, especially in patients with clinical failure, prior azole exposure, or breakthrough infection. In aspergillosis, detection of *cyp51A* mutations may be particularly relevant when culture does not grow or when the patient progresses despite azole therapy [10,23,69,70,97,128].

*Main limitations.* Not all antifungal resistance mechanisms are detected by available platforms. The absence of a marker does not exclude resistance, and its presence must be interpreted together with the species, antifungal exposure, drug concentration, infectious focus, and clinical evolution. In addition, genotype-phenotype correlation may be incomplete in some fungal pathogens [82,108,148].

*Impact according to population.* In hematology/HSCT, detection of antifungal resistance is especially relevant because of the frequency of prior prophylaxis and treatment with azoles or echinocandins. In the ICU, it may be important in persistent candidemia, prior echinocandin exposure, or suspected aspergillosis with therapeutic failure. In neonates, its use is less frequent, but it may be considered in persistent candidemia or species with reduced antifungal susceptibility [10,97].

*Practical mitigation—AFSP-oriented approach.* AFSP-Dx should define “alert pathogens” and activation scenarios: persistent candidemia, breakthrough infection, prior antifungal exposure, clinical failure, suspected *C. auris*, *N. glabratus*, *P. kudriavzevii*, azole-resistant *A. fumigatus*, or emerging molds. Molecular results should be integrated with phenotypic susceptibility profiles when available, since molecular detection of antifungal resistance complements, but does not replace, clinical interpretation or phenotypic testing [10,23,65,70,97,108].

#### 6.6.4. T2 Magnetic Resonance/T2Candida

T2 Magnetic Resonance (T2^MR^) integrates molecular amplification and magnetic resonance to detect pathogens directly in whole blood. T2*Candida* allows rapid detection of common *Candida* species without the need for prior culture isolation, which may be particularly useful in suspected candidemia, where blood cultures may take time to become positive and have limited sensitivity [150,151,152].

*Strengths.* It allows direct detection in whole blood, with less dependence on culture growth. It may shorten the time to identification of species included in the panel and support earlier decisions regarding antifungal initiation, adjustment, or de-escalation in selected scenarios [150,151].

*Main limitations.* Its application is limited by platform availability, associated costs, and restricted coverage to pathogens included in the panel. Therefore, a negative result does not exclude deep IC without fungemia, mold-related IFIs, cryptococcosis, or endemic mycoses. It also does not replace blood cultures, antifungal susceptibility testing, or targeted evaluation of deep or uncontrolled foci [150,151].

*Impact according to population.* In the ICU, it may have potential utility in patients with sepsis or shock without a clear focus and at high risk for candidemia. In hematology/HSCT, it may support the evaluation of breakthrough candidemia, although it does not replace studies for molds. In neonates and pediatrics, its appeal lies in the possibility of working with low-volume samples, but implementation should be adapted to local validation, availability, and institutional protocols [151,152].

*Practical mitigation—AFSP-oriented approach.* T2*Candida* should be integrated as a complementary tool within candidemia pathways, not as a substitute for blood cultures. AFSP-Dx should define activation criteria, interpretation of negative results, microbiologic confirmation, the need for antifungal susceptibility testing, and source-control decisions. Its greatest potential value lies in reducing diagnostic uncertainty time, especially when accompanied by structured clinical reassessment at 48–72 h [151,152].

#### 6.6.5. Rapid Immunochromatographic Tests—LFA/LFD

Lateral-flow immunochromatographic tests—LFA/LFD—allow detection of antigens or antibodies within minutes and may support early decisions when interpreted within a clinical algorithm. These include, among others, tests for CrAg and rapid tests related to aspergillosis, such as GM-LFA or *Aspergillus* LFD, depending on local availability and the platform used [6,121,136,153,154].

*Strengths.* Their main advantage is rapidity, operational simplicity, and potential near-point-of-care use. In cryptococcosis, CrAg LFA is particularly useful when requested in the appropriate clinical syndrome. In aspergillosis, rapid tests in serum or BAL may complement conventional GM, culture, PCR, and imaging, especially when turnaround time in the conventional laboratory is prolonged [121,130,136,154].

*Main limitations.* Not all LFA/LFD assays have the same performance or the same degree of clinical validation. Their performance depends on the sample type, population evaluated, cutoff value, immunosuppression, antifungal prophylaxis, and pretest probability. An isolated positive result may lead to overtreatment, whereas a negative result does not exclude IFI in high-risk patients [82,130,136].

*Impact according to population.* In the ICU, they may support rapid decision-making, but they require integration with criteria adapted to critically ill patients. In hematology/HSCT, they may complement preemptive strategies and accelerate decisions when combined with imaging studies and targeted sampling. In neonates, their role depends on specific validation, availability, and clinical context [136].

*Practical mitigation—AFSP-oriented approach.* LFA/LFD should be incorporated with clearly defined use criteria: population, sample, clinical question, and expected action. AFSP-Dx should prevent them from becoming decontextualized binary tests. Their greatest value lies in shortening decision times and activating structured diagnostic or therapeutic pathways [31,154].

#### 6.6.6. Rapid Molecular Panels from Positive Blood Cultures: BioFire BCID2 and ePlex BCID-FP

Rapid molecular panels applied to positive blood cultures, such as BioFire BCID2 and GenMark/cobas ePlex BCID-FP, allow faster identification of yeasts and other fungal pathogens recovered from blood. Their main utility is not to replace blood cultures, but to reduce the interval between culture positivity, agent identification, and initial therapeutic adjustment. This approach may be especially relevant in candidemia, suspected *C. auris* infection caused by non-*albicans* species, and episodes of fungemia in critically ill or immunosuppressed patients [87,88]. BioFire BCID2 is designed for positive blood cultures and reports multiple ITS-associated targets, including yeasts, whereas ePlex BCID-FP corresponds to a fungal-specific panel for positive blood cultures [87,155].

*Strengths.* BioFire BCID2 allows rapid identification of several ITS-associated pathogens, including common *Candida* species and *Cryptococcus neoformans/gattii*, as well as some bacterial resistance markers. In turn, ePlex BCID-FP offers a broader fungal panel for positive blood cultures, with detection of multiple *Candida*, *Cryptococcus*, *Fusarium*, and *Rhodotorula* species, among other targets included in the platform. In both cases, the clinical value lies in shortening the time to identification, activating control measures when *C. auris* is suspected, and facilitating early decisions regarding antifungal adjustment or de-escalation [87,88,155]. The ePlex BCID-FP panel reports *Fusarium* and *Rhodotorula* at the genus level, an important detail to avoid overinterpreting the result as complete species-level identification.

*Main limitations.* These systems detect only the organisms included in the panel and generally require a prior positive blood culture; therefore, they do not solve the problem of limited blood culture sensitivity, deep candidiasis without documented fungemia, or IFIs caused by non-circulating molds. In addition, they do not replace antifungal susceptibility testing, evaluation of deep-seated foci, or clinical interpretation of the episode. A negative panel result does not exclude an IFI caused by pathogens outside panel coverage, and a positive result must be integrated with the species identified, site of isolation, clinical context, prior antifungal exposure, and probability of contamination or true infection [87,155].

*Impact according to population.* In the ICU, these panels may accelerate identification in candidemia and support early decisions regarding treatment, antifungal de-escalation, or source control. In hematology/HSCT, they may provide value in breakthrough fungemia, prior antifungal exposure, or suspected emerging yeasts; however, they do not replace targeted evaluation for mold disease or the search for tissue involvement. In neonates, their potential utility depends on recovery of the microorganism in blood culture, and therefore they do not eliminate the limitations derived from low sample volumes and suboptimal sensitivity [155].

*Practical mitigation—AFSP-oriented approach.* AFSP-Dx should define how BCID2 or ePlex results will be used within candidemia and fungemia pathways: immediate communication of alert yeasts, species confirmation when necessary, requests for antifungal susceptibility testing, CVC evaluation, investigation of deep-seated foci, and early therapeutic reassessment. These panels should be understood as tools that accelerate identification from positive blood cultures, not as exclusion tests for IFIs [155,156].

### 6.7. An Additional Problem: Diagnostic Criteria Are Not Always “Transferable” Across Populations

Standardized IFI definitions, such as those proposed by EORTC/MSGERC, have been fundamental for clinical research, comparability among studies, and support for clinical practice [22]. However, they were developed primarily in populations with hematologic malignancies and HSCT, so their direct application to non-neutropenic critically ill patients, neonates, or other non-classical scenarios may be limited [21,41,72,83].

In the ICU, many patients present risk factors, radiologic findings, biomarker patterns, microbiologic performance, and clinical patterns that differ from those observed in classical hematology. As a consequence, the non-adapted use of these criteria may favor underdiagnosis, uncertain classification, or therapeutic delay in entities such as influenza-associated aspergillosis, severe COVID-19, or other forms of ICU-associated aspergillosis [9,46,84,106].

In non-neutropenic critically ill patients, interpretation of respiratory cultures, serum or BAL GM, radiologic findings, and clinical evolution requires context-adapted criteria, since the distinction between colonization and invasive infection differs from classical hematologic scenarios [7,63,82,107,129].

This limitation has driven the development of diagnostic algorithms and definitions specific to critically ill patients. The AspICU algorithm was designed to distinguish respiratory colonization by *Aspergillus* from putative pulmonary aspergillosis in the ICU, given that a substantial proportion of critically ill patients with positive respiratory cultures could not be classified using classical EORTC/MSG criteria. In its multicenter validation, the algorithm demonstrated utility in discriminating colonization from invasive disease in critically ill patients with respiratory isolation of *Aspergillus* spp. [21,83].

In addition, variants such as BM-AspICU incorporate biomarkers into diagnostic reasoning, especially GM in BAL or serum, in order to increase diagnostic sensitivity in critically ill patients. For specific viral scenarios, the ECMM/ISHAM criteria for CAPA were developed to classify COVID-19-associated pulmonary aspergillosis, whereas approaches for influenza-associated aspergillosis seek to address a similar problem: the limited transferability of classical criteria to critically ill patients with severe viral pulmonary injury [81,130,147].

More recently, initiatives such as FUNDICU have proposed standardized IFI definitions in non-neutropenic critically ill adults, recognizing that IC and IA in the ICU have risk factors, diagnostic tools, and clinical patterns that differ from those observed in hematologic populations. These frameworks do not replace EORTC/MSGERC definitions, but they help contextualize them and reduce diagnostic uncertainty in scenarios where their direct application may be insufficient [21,41,56,138].

This phenomenon explains part of the diagnostic failure in critically ill patients and supports the development of diagnostic algorithms specific to the clinical scenario, rather than automatically extrapolating definitions developed for other populations. The utility of these algorithms does not lie in replacing existing diagnostic criteria, but in translating them into clinical pathways that are more applicable, context-sensitive, and oriented toward timely decision-making [32,46,84,130].

**Figure 1:**
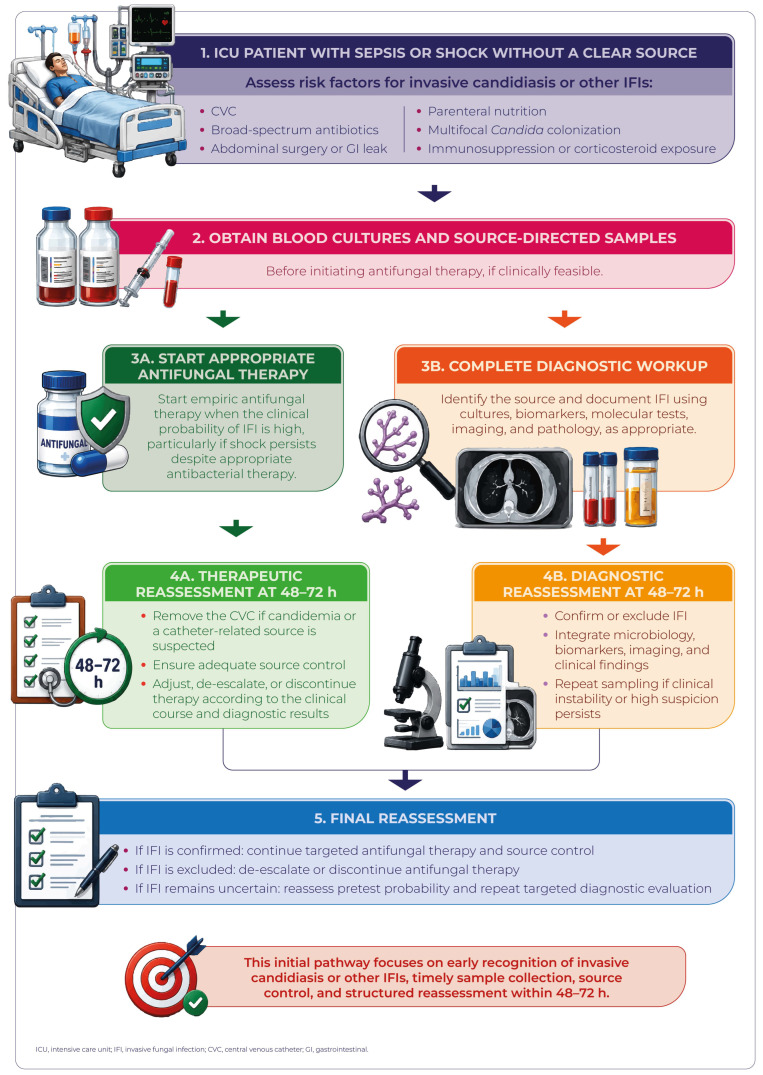
ICU algorithm for sepsis or shock without a clear source and suspected invasive fungal infection. The algorithm outlines an integrated diagnostic–therapeutic pathway for critically ill patients with persistent sepsis or shock and risk factors for invasive candidiasis or other invasive fungal infections. It emphasizes early risk recognition, blood cultures and source-directed sampling before antifungal therapy whenever clinically feasible, initiation of empiric antifungal therapy when the clinical probability of IFI is high, integrated diagnostic workup, source control, and structured reassessment at 48–72 h. Final decisions should integrate clinical course, microbiology, biomarkers, imaging, and local epidemiology to confirm IFI, de-escalate or discontinue antifungal therapy, or repeat targeted diagnostics when uncertainty persists.

In critically ill patients with sepsis or shock without a clear focus, an initial pathway aimed at early recognition of a possible IFI, prioritizing timely sampling, evaluating source control, initiating appropriate therapy when justified by clinical probability, and performing early reassessment is summarized in Figure 1.

**Figure 2:**
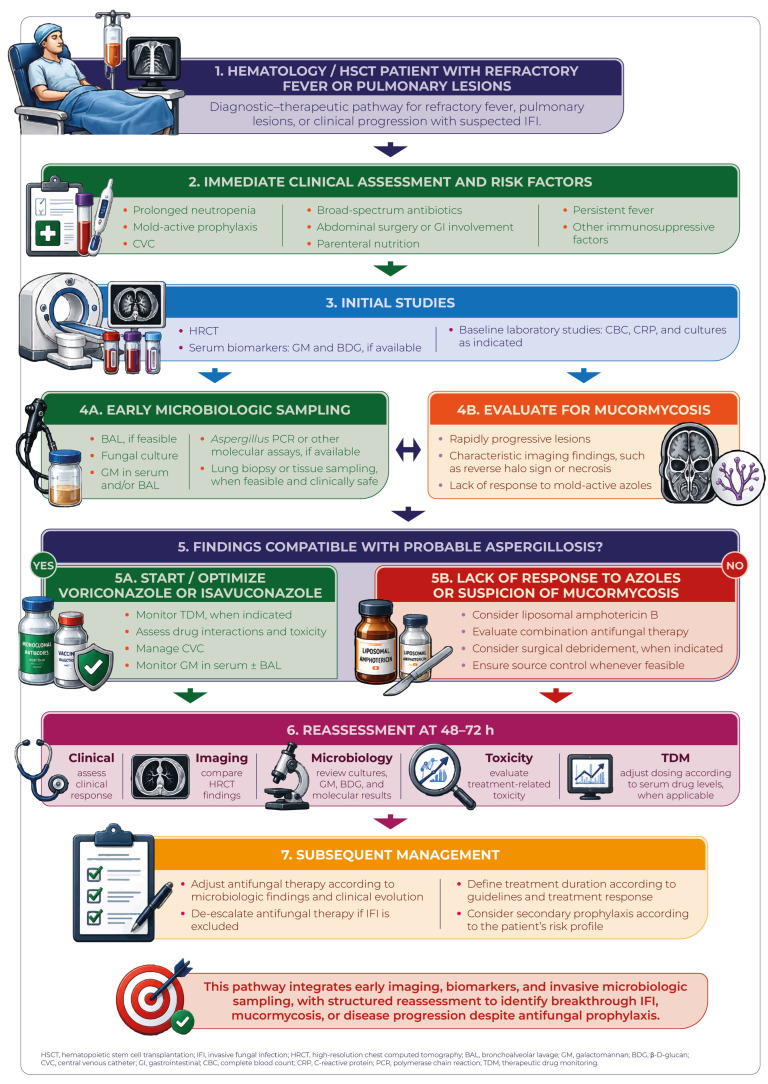
Diagnostic algorithm for hematology and hematopoietic stem cell transplant patients with suspected invasive fungal infection. This algorithm provides a practical diagnostic–therapeutic pathway for patients with hematological malignancies or HSCT who develop refractory fever, new pulmonary lesions, or clinical progression despite antibacterial therapy or mold-active prophylaxis. The pathway emphasizes early HRCT, contextual interpretation of fungal biomarkers, and prompt microbiological sampling, including BAL or tissue biopsy when feasible and safe. Because breakthrough infection may reflect pathogens not covered by prior prophylaxis, progressive lesions, necrosis, suggestive imaging, or poor response to mold-active azoles should prompt active evaluation for mucormycosis and other non-Aspergillus molds. Reassessment at 48–72 h integrates clinical response, radiologic evolution, microbiology, toxicity, and therapeutic drug monitoring to guide treatment optimization, escalation, de-escalation, or secondary prevention.

In hematologic patients or HSCT recipients, where prolonged neutropenia, antifungal prophylaxis, breakthrough infection, and emerging pathogens modify clinical presentation and diagnostic performance, a higher-intensity diagnostic approach is required. This approach, based on early imaging, biomarkers, invasive sampling when feasible, and rapid reassessment in the setting of progression or lack of response, is summarized in Figure 2.

**Figure 3:**
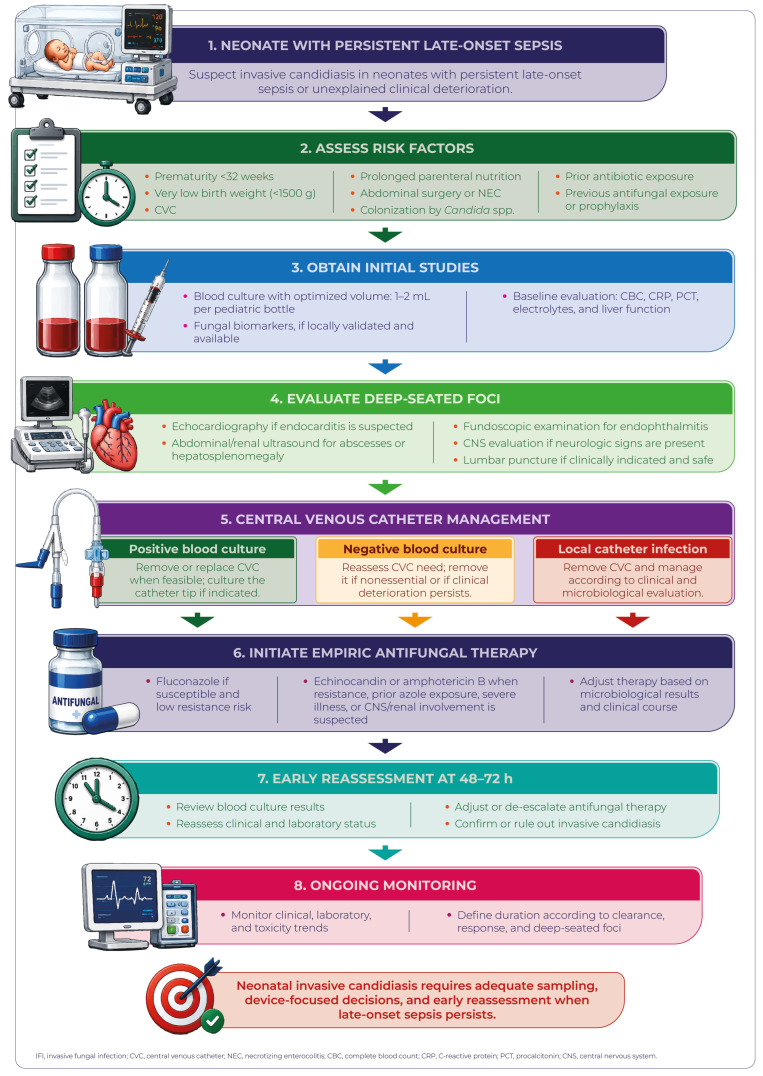
Neonatal algorithm for persistent late-onset sepsis and suspected invasive candidiasis. This algorithm outlines a practical pathway for preterm or very-low-birth-weight neonates with persistent late-onset sepsis or unexplained clinical deterioration in whom invasive candidiasis should remain in the differential diagnosis. The approach prioritizes adequate blood culture volume, cautious use of non-culture biomarkers, early assessment of catheter-related infection, and targeted evaluation of deep foci, including renal, ocular, cardiac, and central nervous system involvement when clinically indicated. Empiric antifungal therapy should be guided by clinical probability, previous azole exposure, local epidemiology, illness severity, and microbiological results. Reassessment at 48–72 h supports timely adjustment, de-escalation, or continuation of targeted therapy.

In premature neonates or VLBW infants with persistent LOS, where IC often mimics bacterial sepsis and blood cultures have relevant limitations because of low sample volume, the diagnostic and therapeutic pathway must be adapted to baseline risk, the role of the CVC, the search for deep-seated foci, and early reassessment. This approach is summarized in Figure 3.

### 6.8. Central Implication for Reducing Diagnostic Failure

Reducing diagnostic failure requires more than expanding access to diagnostic tests; these tools must be integrated into a syndromic and dynamic decision-making process. In IFIs, diagnostic accuracy depends on pretest probability, timely sampling, appropriate selection of complementary methods, and serial clinical reassessment [17,31,37,68,129,134].

This approach requires multidisciplinary interpretation of microbiological, biomarker, imaging, histopathological, molecular, and epidemiological findings, without relying on any single result in isolation. Coordination among clinicians, infectious disease specialists, microbiologists, mycologists, radiologists, pathologists, and clinical pharmacists can reduce attribution errors, shorten diagnostic timelines, and support more precise therapeutic decisions [6,7,65,81,82,125,129,130,131].

Imaging should be integrated with host characteristics, clinical evolution, the anatomical pattern of involvement, and pretest probability. In aspergillosis, mucormycosis, endemic mycoses, and focal lesions of uncertain etiology, it may guide sampling, biopsy or surgical assessment, evaluation of disease extent, and reassessment when response is inadequate. Population-specific pathways and formal reassessment at 48–72 h should then guide diagnostic intensification, antifungal escalation or de-escalation, and source-control decisions according to the clinical syndrome, host type, suspected pathogen, and local availability [6,17,20,31,37,45,57,58,59,78,84,86,87,89,128,157].

**Figure 4:**
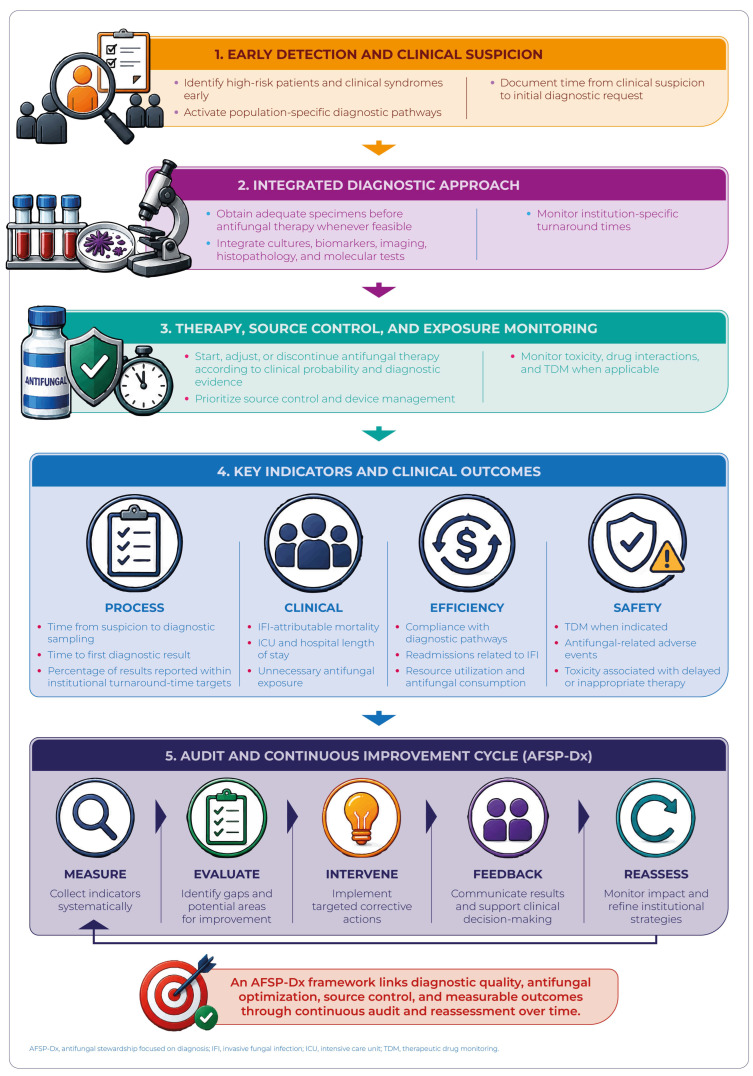
Operational framework to measure and reduce diagnostic failure in invasive fungal infections through integrated indicators and AFSP-Dx. This framework translates the AFSP-Dx concept into an institutional model for identifying, measuring, and reducing diagnostic failure in invasive fungal infections. It links early clinical suspicion, coordinated diagnostic workflows, antifungal decision-making, source control, exposure monitoring, and predefined indicators within a continuous audit cycle. Rather than functioning as a clinical algorithm for a single syndrome, the framework is intended to guide local implementation: institutions should define population-specific triggers, monitor diagnostic and therapeutic processes, identify bottlenecks, and use feedback loops to improve diagnostic quality, antifungal precision, patient safety, and measurable outcomes.

These practical approaches are integrated in Table 2, whereas Figure 4 proposes an operational framework to measure and reduce diagnostic failure in IFIs through integrated indicators, institutional auditing, and improvement cycles linked to AFSP-Dx.

## 7. Clinical Consequences of Diagnostic Failure in IFIs and Measurement Through Indicators in ICU, Hematology/HSCT, and Neonatology

Diagnostic failure—whether delayed, erroneous, or incomplete—in IFIs is consistently associated with worse clinical outcomes and greater use of healthcare resources. Its impact extends beyond diagnostic delay itself, since it modifies the timing of therapeutic initiation, leads to incorrect therapeutic decisions, and favors more complex care pathways [7,8,11,24,35,43,81,90,93].

Across clinical settings, four major consequences may be recognized: (i) increased attributable mortality, especially when active treatment is delayed; (ii) increased morbidity, anatomic progression of infection, and functional sequelae; (iii) drug toxicity or harm related to unnecessary antifungal overtreatment; and (iv) selective pressure with emergence of antifungal resistance, increased costs, and prolonged hospital stay [17,23,31,52,53,66,67,70,96,97,102,158,159,160,161].

The relative magnitude of each consequence varies according to the affected population. In the ICU, early mortality, delayed source control, and prolonged critical support predominate; in hematology/HSCT, rapid progression under immunosuppression, breakthrough infections, incomplete etiologic diagnosis, and therapeutic failure stand out; whereas in neonatology, mortality, the difficulty of documenting invasive disease with limited sample volumes, and long-term neurodevelopmental sequelae acquire particular relevance [28,53,54,90,114,126,158,162,163].

From a healthcare quality perspective, these consequences should be measured through clinical and process indicators, since only what is systematically monitored can be improved. Therefore, evaluation of diagnostic failure also constitutes a strategic tool for AFSPs, patient safety, and continuous improvement, especially when it allows linkage of diagnostic timelines, therapeutic adequacy, etiologic identification, antifungal susceptibility, timely use of rapid tests, source control, pharmacologic consumption, and clinical outcomes in high-risk populations [17,31,32,37,61,94,134].

### 7.1. Attributable Mortality and the Therapeutic “Critical Window”

The most direct consequence of diagnostic failure in IFIs is the loss of therapeutic opportunity. In this context, delay should not be understood merely as an administrative or microbiologic delay, but rather as a clinically active interval during which infection progresses, inflammatory or tissue burden increases, organ dysfunction accumulates, and the probability of response decreases. Therefore, the concept of a “critical window” is particularly useful: in certain IFIs, especially candidemia in critically ill patients, mucormycosis, and neonatal candidiasis, hours or a few days may substantially modify prognosis [24,28,39,90,114,158,162,163].

*Intensive care unit (ICU).* In critically ill patients with candidemia or *Candida*-associated septic shock, multiple cohorts have demonstrated that the combination of early source control and timely administration of an active antifungal agent is associated with lower in-hospital mortality compared with delayed or incomplete strategies [24,39,41,138].

Consistently, classic studies show that waiting for definitive microbiologic confirmation—for example, a positive blood culture—before initiating treatment in patients with high clinical suspicion is associated with worse survival and greater progression of organ dysfunction [4,24,43,131,162].

These findings support the concept of a “therapeutic critical window,” in which delays measured in hours may translate into relevant clinical consequences. Operationally, this window is not limited to timely antifungal initiation, but also includes early acquisition of useful samples, evaluation of the CVC as a possible source, drainage or source control when appropriate, and early reassessment of clinical response.

*Hematology/HSCT.* In hematologic patients and HSCT recipients, although the exact effect varies according to the pathogen involved—*Aspergillus*, Mucorales, *Fusarium*, or others—the common denominator is that diagnostic delay perpetuates inadequate initial therapy and postpones decisive interventions [20,49,84,113,114].

Classic examples include use of azoles without activity against unrecognized mucormycosis, delay in escalating treatment in the setting of breakthrough infection, and delay in activating surgery for source control when indicated. These clinical patterns are associated with persistently high mortality in contemporary series [10,20,66,67,93,96,116].

In this population, the critical window is not necessarily measured in a few hours from the onset of sepsis, but rather in the progressive loss of opportunities to establish the etiology, distinguish aspergillosis from mucormycosis, identify uncovered pathogens, and act before tissue progression or persistent immunosuppression reduce the probability of therapeutic response.

*Neonatology.* In ELBW neonates, IC is associated with a particularly adverse composite outcome that includes death or long-term neurodevelopmental impairment [30,85]. In addition, *Candida* meningitis may occur with negative blood cultures in a clinically relevant proportion of cases, which limits the reassuring value of a negative blood culture in high-risk neonates with persistent clinical suspicion [14,26,29,64].

In neonatology, the critical window is conditioned by low clinical specificity, the small blood volumes available for blood cultures, the need to evaluate deep-seated foci, and early decision-making regarding the CVC. Therefore, mortality and sequelae depend not only on initiation of an antifungal agent, but also on integrating treatment, source control, and evaluation for dissemination.

#### Practical Implication

Attributable mortality related to diagnostic failure depends not only on the microorganism, but also on the elapsed time until initiation of appropriate antifungal therapy and on addressing the modifiable determinants of the healthcare process. Therefore, measuring and reducing the interval between clinical suspicion, sample collection, appropriate treatment, and source control constitutes one of the central quality-of-care objectives in IFIs.

### 7.2. Morbidity, Complications, and Sequelae Beyond Mortality

Although mortality is the most visible outcome of diagnostic failure in IFIs, it does not fully capture its clinical impact. A delayed, erroneous, or incomplete diagnosis may also prolong organ dysfunction, increase the need for invasive support, favor anatomic progression of infection, and generate persistent functional sequelae. These consequences are especially relevant because they increase healthcare complexity even in patients who survive the acute episode [4,9,24,40,41,46,63,84,138,159].

*Intensive care unit (ICU).* In the ICU, delayed diagnosis of an IFI not only increases mortality, but also prolongs dependence on critical support. It frequently translates into longer duration of vasopressor use, prolonged mechanical ventilation, and progressive accumulation of organ dysfunction [4,18,83,131].

Likewise, delays in initiating targeted therapy or achieving source control promote persistent or recurrent infections, the need for additional procedures, and prolonged intensive care stays [59,74].

*Hematology/HSCT.* In hematologic patients and HSCT recipients, the typical consequence of incomplete or erroneous diagnosis is progression of angioinvasive disease, with greater tissue extension and irreversible damage [84,163].

This may translate into the need for more toxic salvage therapies, complex antifungal combinations, and late—or no longer feasible—invasive procedures when the patient presents clinical deterioration, severe thrombocytopenia, or multiorgan involvement [160,164].

In this group, morbidity also derives from prolonged maintenance of initially inactive or insufficient treatments, especially when mucormycosis, breakthrough infection, antifungal resistance, or pathogens not covered by the initial regimen are not recognized in a timely manner. This progression may reduce the possibility of local control, limit continuity of oncohematologic treatment, and increase exposure to cumulative drug toxicity [20,60,66,67,75,93].

*Neonatology.* In neonates, the burden of disease clearly extends beyond mortality. IC is associated with a relevant risk of neurodevelopmental sequelae, neurologic abnormalities, and greater subsequent healthcare complexity [30,85].

In addition, decisions dependent on timely diagnosis—such as early CVC removal, targeted evaluation of metastatic foci, or rapid adjustment of antifungal therapy—are associated with better clinical outcomes [28,44,64,99].

#### Practical Implication

Morbidity associated with diagnostic failure is usually less visible than mortality, but it represents a considerable clinical and healthcare burden. Therefore, quality indicators in IFIs should not be limited to survival, but should also include duration of critical care support, anatomic progression, need for salvage therapy, source control, exposure to drug toxicity, and functional sequelae.

### 7.3. Overtreatment, Drug Toxicity, and Collateral Harm: The “Clinical Cost” of Diagnostic Uncertainty

Diagnostic failure not only leads to undertreatment; it also favors the opposite phenomenon: broad, early, and prolonged empiric antifungal therapy in patients without confirmed IFI or with ultimately low clinical probability. This pattern is particularly frequent in the ICU, where patient severity and fear of delaying therapy drive early antifungal treatment decisions [17,31,61,94,134].

Multicenter European studies have shown that a relevant proportion of patients receiving systemic antifungal therapy in intensive care settings do not have documented IFIs, reflecting the burden of diagnostic uncertainty in real-world practice and the difficulty of discontinuing antifungal therapy once initiated [17,31,32,61].

This “clinical cost” of diagnostic uncertainty should not be interpreted solely as pharmacologic consumption. In practice, overtreatment may expose the patient to drug toxicity, pharmacologic interactions, selective pressure, additional monitoring, and delay in the search for the true etiology. In addition, for antifungal agents with high pharmacokinetic variability, especially triazoles, the absence of TDM when indicated may perpetuate suboptimal or toxic exposures, making it difficult to distinguish among pharmacokinetic failure, preventable drug toxicity, and true microbiologic failure [6,78,100,160,165]. Therefore, the harm associated with diagnostic failure includes both failure to treat a true IFI in a timely manner and maintenance of antifungal therapy when the overall probability of IFI no longer justifies it.

*Clinical consequences of overtreatment.* Antifungal overtreatment is associated with multiple clinically relevant adverse events. Among the most important are:

• Nephrotoxicity, especially with amphotericin B formulations;
• Hepatotoxicity and complex pharmacologic interactions with azoles;
• Electrolyte abnormalities, including hypokalemia and hypomagnesemia;
• Complications related to catheters, additional monitoring, or the need for TDM, especially with triazoles such as voriconazole, posaconazole, or itraconazole;
• Suboptimal or toxic antifungal exposure when TDM is not performed in scenarios where it is indicated, which may favor both therapeutic failure and preventable adverse events [78,100,165];
• Displacement of diagnostic decision-making when antifungal coverage is used without completing an adequate etiologic investigation [17,31,32,61,102].

#### Implications for AFSP

From the AFSP perspective, the objective is not to avoid empiric antifungal therapy when justified, but rather to limit its unnecessary duration through early reassessment, protocolized de-escalation, and improved diagnostic integration. Each additional day of unjustified treatment increases potential drug toxicity, costs, and pharmacological selective pressure [17,31,32,37,102].

Operationally, every empiric antifungal agent should have a documented indication, an explicit diagnostic objective, and a formal reassessment point at 48–72 h. This reassessment should integrate clinical evolution, microbiologic results, biomarkers, imaging studies, pretest probability, availability of alternative diagnoses, and, when appropriate, TDM to optimize exposure, minimize drug toxicity, and distinguish pharmacokinetic failure from microbiologic or diagnostic failure [78,100,165]. Only in this way does empiric treatment cease to be an indefinite response to uncertainty and become a temporary, evaluable, and safe intervention.

### 7.4. Antifungal Resistance and Breakthrough Fungemias: When Diagnostic Failure Selects the Next Problem

Prolonged empiric use and repeated exposure to antifungal agents constitute one of the most relevant consequences of diagnostic failure, since they increase the probability of selecting less susceptible species and favor the emergence of acquired antifungal resistance [10,43,65,70,96,108,111,166].

This phenomenon is especially visible when diagnostic uncertainty leads to prolonged treatment without microbiologic confirmation, sequential changes in antifungal class, or prolonged antifungal prophylaxis in high-risk patients. In these scenarios, pharmacological selective pressure modifies the institutional fungal ecology and may favor subsequent episodes that are more difficult to diagnose and treat [23,70,96].

#### 7.4.1. Clinically Relevant Examples

A classic example is *N. glabratus*, in which prior exposure to echinocandins has been associated with isolates harboring mutations in *FKS* genes, linked to reduced antifungal susceptibility and a greater probability of clinical failure during echinocandin treatment [24,36,132,139]. This example illustrates how a strategy initially motivated by diagnostic uncertainty may later become a complex therapeutic problem.

Another relevant example is *C. auris*, whose clinical impact combines antifungal resistance, difficulty of identification by conventional methods, and capacity for nosocomial transmission. In this case, diagnostic failure not only delays selection of active therapy, but also outbreak prevention and control measures, thereby amplifying the institutional impact of delayed or incorrect identification [70,76,125].

In mold infections, azole resistance in *A. fumigatus* constitutes an example of incomplete diagnosis with direct therapeutic implications. In patients with prior azole exposure, clinical progression during treatment, or compatible local epidemiology, assuming antifungal susceptibility may delay transition to an active antifungal regimen and prolong ineffective therapy [19,78,96,108].

In addition, cryptic species within *Candida*, *Aspergillus*, *Fusarium*, *Scedosporium*, or other opportunistic fungal complexes may represent a less evident form of incomplete diagnosis. Although the isolate may be recognized as a yeast or mold, identification limited to the genus or complex level may conceal distinct susceptibility profiles, intrinsic resistance, or reduced clinical response to the selected antifungal agent. Therefore, in scenarios of therapeutic failure, prior antifungal exposure, breakthrough infection, or isolation of unusual pathogens, more precise identification and antifungal susceptibility testing acquire direct clinical relevance [60,75,105,108,111,131].

Species with intrinsic antifungal resistance or unpredictable susceptibility should also be considered, such as *P. kudriavzevii*, *L. prolificans*, *Fusarium* spp., *Scedosporium* spp., *Trichosporon* spp., and *Saprochaete* spp. In these pathogens, delayed identification at the species or species complex level may translate into initially ineffective treatment and higher mortality, especially in hematologic patients or those with profound immunosuppression [48,49,50,60,75,167].

#### 7.4.2. Breakthrough Fungemias and Epidemiologic Shift

Prior exposure to antifungal agents also favors breakthrough fungemias caused by intrinsically less susceptible species, cryptic species with unrecognized susceptibility profiles, or fungi not covered by the antifungal class used. This includes non-*albicans* yeasts, rare yeasts, emerging molds, and pathogens selected during prolonged antifungal prophylaxis or treatment [52,53,54,66,67,161].

These breakthrough infections are particularly relevant in patients receiving antifungal prophylaxis or prolonged empiric treatment, since they may present with low microbiologic burden, atypical clinical manifestations, and initially negative diagnostic tests. In these scenarios, incomplete diagnosis favors continuation of inactive therapy, delays changes in antifungal class, and increases selective pressure within the institution [53,66].

In practical terms, a breakthrough fungemia should not be interpreted solely as “antifungal failure,” but rather as a diagnostic warning signal. It may reflect a pathogen not covered by prophylaxis, a species with reduced susceptibility, incomplete identification, acquired resistance, suboptimal pharmacologic exposure, or a persistent uncontrolled focus. Therefore, it should trigger a structured reassessment including identification at the species or species complex level, antifungal susceptibility testing, review of prior exposure, TDM when appropriate, source investigation, and analysis of possible epidemiologic implications [10,43,96,100,111,125,127,165].

#### 7.4.3. Conceptual Implication

From an AFSP-Dx perspective, diagnostic failure should not be understood as an isolated event ending in an incorrect therapeutic decision, but rather as a mechanism capable of reshaping subsequent risk for both the patient and the hospital ecosystem. Sustained diagnostic uncertainty promotes broad antifungal exposure; this, in turn, increases selective pressure and may facilitate antifungal resistance or breakthrough infections. The resulting episode is often more difficult to recognize, identify, and treat, thereby perpetuating escalating diagnostic and therapeutic complexity.

This phenomenon may be understood as a clinical and microbiological feedback loop in which diagnostic uncertainty leads to broad antifungal exposure, selective pressure, antifungal resistance or persistent infection, and ultimately to new scenarios of greater diagnostic difficulty.

Interrupting this cycle requires earlier diagnosis, reliable species-level or species complex-level identification, antifungal susceptibility testing when clinically relevant, frequent therapeutic reassessment, and robust AFSP-Dx [31,32,61].

### 7.5. Length of Hospital Stay and Attributable Costs: Institutional Indicators for AFSP

In candidemia and other IFIs, observational studies with matched controls have shown significant increases in length of hospital stay, ICU days, use of critical care resources, and episode-related costs, together with excess attributable mortality [159,168,169,170]. These clinical outcomes are important because they translate diagnostic failure into objective institutional metrics. Each delay in recognizing an IFI may prolong the need for MV, vasopressor support, invasive procedures, multiple antimicrobial therapies, and hospitalization [59,103,171].

From an institutional perspective, these indicators show that diagnostic failure is not only an individual clinical problem, but also a source of healthcare inefficiency. Delays in suspecting infection, obtaining diagnostic samples, identifying the etiologic agent, documenting antifungal susceptibility when clinically relevant, initiating appropriate treatment, or achieving source control may accumulate avoidable hospital days, unnecessary pharmacologic exposure, and sustained use of high-cost resources [17,31,32,61,94,102].

*Useful institutional indicators for AFSP.* From the AFSP perspective, these outcomes may be translated into a limited set of tracer indicators capable of linking the diagnostic process with therapeutic decisions and institutional outcomes. The objective is not to accumulate metrics, but to select variables that allow recognition of where diagnostic opportunities are lost, where unnecessary antifungal exposure is prolonged, and which interventions modify the clinical course. In this context, the most useful indicators include:

• Mean hospital and ICU length of stay in patients with IFIs;
• Direct episode-related costs (drugs, ICU care, procedures, laboratory testing);
• Time from clinical suspicion to appropriate therapy;
• Time to etiologic identification and antifungal susceptibility profile when appropriate;
• Antifungal therapy days per episode;
• Need for TDM and proportion of dose adjustments derived from suboptimal or potentially toxic levels, especially with triazoles [78,100,165];
• 14- and 30-day mortality;
• Proportion of empiric antifungal therapy discontinued after diagnostic reassessment [31,37,103].

#### Strategic Utility

These metrics are useful for justifying institutional investment in biomarkers, molecular assays, rapid panels from positive blood cultures, strengthening microbiology capacity, reliable identification, antifungal susceptibility testing, and AFS teams, as they allow estimation of the clinical and economic return of early diagnostic interventions.

In other words, reducing diagnostic failure not only improves survival, but may also free critical care beds, reduce avoidable costs, and optimize the use of hospital resources. For this argument to be robust, AFSPs must link their interventions to measurable clinical outcomes that are comparable over time, rather than limiting reporting to antifungal consumption or the number of recommendations issued [17,31,37].

### 7.6. Proposed Indicators for Measuring and Auditing Diagnostic Failure, Adjusted by Population

Measuring diagnostic failure requires consideration of diagnostic timeliness, therapeutic appropriateness, source control, the completeness of microbiological characterization, antifungal exposure, and clinical outcomes. Because the relevance of these domains varies among ICU, hematology/HSCT, and neonatal settings, we propose combining a core set of institutional indicators with additional population-specific measures. This proposal also draws on quality-assessment initiatives that translate recommendations for the management of fungal diseases into measurable standards of care [17,21,30,31,60,61,65,75,77,78,83,100,103,147,165,171,172,173]. The proposed core indicators are summarized in Table 3, while Figure 4 illustrates how process indicators, clinical outcomes, antifungal exposure, and institutional improvement cycles can be integrated within the AFSP-Dx framework.

#### 7.6.1. Cross-Cutting Indicators: All Populations

These indicators provide a common basis for evaluating diagnostic performance across hospital settings and clinical services. Their purpose is not to measure every possible step in the diagnostic process, but to identify critical points at which diagnostic opportunities are lost, appropriate treatment is delayed, relevant microbiological information is not obtained, or antifungal exposure is unnecessarily prolonged.

A cross-cutting set of indicators may include:

• time to documented suspicion: the interval from the onset of sepsis, persistent fever, or otherwise unexplained clinical deterioration to the first clinical note that includes IFI in the differential diagnosis;
• time to the first useful diagnostic action: the interval until blood cultures and at least one relevant complementary test—such as a biomarker, imaging study, or specimen from the suspected focus—are requested according to the clinical syndrome and local protocol;
• time to appropriate antifungal therapy: the interval until initiation of an antifungal agent expected to be active against the pathogen ultimately identified or considered most likely, at an appropriate dose;
• time to etiologic identification: the interval from the first positive specimen or relevant microbiological finding to identification at the species or species complex level when clinically relevant;
• time to antifungal susceptibility results: the interval until susceptibility data become available when testing is indicated because of the species involved, prior antifungal exposure, breakthrough infection, therapeutic failure, or suspected resistance;
• time to source control: the interval until CVC removal, drainage, debridement, or surgery when clinically indicated;
• proportion of empiric antifungal therapy without documented IFI: the number of treated patients without subsequent evidence of IFI per 100 exposed patients, used as a measure of persistent diagnostic uncertainty and potential opportunity for de-escalation [31,61];
• days of antifungal therapy per 1000 admissions or patient-days, stratified by empiric, preemptive, or targeted use;
• proportion of patients in whom TDM was indicated and performed, particularly among those receiving triazoles with high pharmacokinetic variability, relevant drug interactions, suspected toxicity, or therapeutic failure [78,100].

#### 7.6.2. Intensive Care Unit: Critical Window and Source Control

In critically ill patients, indicators should focus on therapeutic timeliness, early acquisition of useful diagnostic samples, and source control. In this context, even brief delays may have prognostic impact, particularly in candidemia with septic shock or aspergillosis associated with critical illness. Therefore, institutional metrics should address three operational questions: whether a useful diagnostic sample was obtained, whether appropriate treatment was initiated on time, and whether control of the infectious focus was achieved [21,41,46,56,59,83,147].

The most relevant indicators include:

• percentage of patients with septic shock and candidemia achieving active antifungal therapy plus source control within ≤24 h;
• time from clinical deterioration to blood culture collection and useful complementary diagnostic testing;
• time from blood culture positivity to rapid pathogen identification when molecular panels from positive blood cultures are used;
• DOT per 1000 ICU-days, stratified by empiric, targeted, or preemptive use;
• rate of antifungal de-escalation at 72–96 h when comprehensive evaluation does not support IFI;
• time from suspicion of ICU-associated aspergillosis to acquisition of a diagnostic respiratory sample [21,41,46,56].

#### 7.6.3. Hematology/HSCT: Incomplete Diagnosis and Antifungal Resistance

In hematologic and HSCT patients, indicators should evaluate the etiologic depth of diagnosis, the timeliness of therapeutic escalation, and detection of pathogens not covered by the initial strategy. In this population, the objective is not only to initiate treatment early, but also to avoid persistence of apparently appropriate therapy against the wrong pathogen. Therefore, metrics should capture institutional capacity to identify species or species complexes with therapeutic implications, document antifungal susceptibility when indicated, and recognize antifungal resistance, breakthrough infection, or early lack of response [52,53,60,65,75,77,78,84,100,107,166].

The most useful indicators include:

• Time to species-level or species complex-level identification and antifungal susceptibility testing when applicable;
• Proportion of *N. glabratus* candidemias with prior echinocandin exposure and documented antifungal resistance or molecular suspicion of *FKS* mutation [36];
• Proportion of candidemias caused by emerging or difficult-to-identify species, including *C. auris*, with confirmed identification and documented antifungal susceptibility [70,125];
• Proportion of antifungal class changes due to lack of clinical or microbiological response—for example, from an azole to amphotericin B—as an indirect indicator of incomplete diagnosis, breakthrough infection, or insufficient initial coverage;
• Time from suggestive radiologic lesion to invasive diagnostic procedure or microbiological confirmation;
• Proportion of patients receiving triazole treatment or prophylaxis in whom TDM is performed when indicated, and proportion of adjustments derived from suboptimal or potentially toxic levels [19,78,100];
• 30-day mortality stratified according to time to etiologic diagnosis.

#### 7.6.4. Neonatology: Limited Blood Culture Sensitivity, Catheter Management, and CNS Involvement

In neonatology, indicators must recognize sampling limitations, the low sensitivity of blood cultures, and the risk of disseminated involvement, including the CNS. In this setting, CVC management and active investigation of infectious foci are central components of the diagnostic-therapeutic process. Therefore, metrics should focus on treatment timeliness, the quality of available sampling, evaluation of deep-seated foci, and microbiological documentation when an isolate is recovered [14,26,30,44,64,85,98].

The proposed indicators include:

• Blood volume per blood culture—when documented—and number of sets per episode;
• Time to CVC removal or replacement in candidemia—≤24 h or ≤72 h according to local protocol;
• Proportion of neonates with candidemia evaluated for CNS involvement, given the possibility of *Candida* meningitis with negative blood cultures;
• Time from initial clinical suspicion to initiation of appropriate antifungal therapy;
• Time to species identification and antifungal susceptibility testing in neonatal candidemia when the isolate is available;
• In-hospital mortality and neurologic follow-up when available [30,85,99].

#### 7.6.5. Institutional Utility

These indicators provide a minimum institutional AFSP-Dx framework that can be adapted to local epidemiology, available resources, and the populations served. Their purpose is to identify diagnostic delays, gaps in microbiological characterization, and unnecessary antifungal exposure, while supporting measurable improvement over time [17,31,61].

## 8. Strategies to Reduce Diagnostic Failure in IFIs: “Bundles” and Population-Specific Diagnostic Algorithms

Reducing diagnostic failure requires coordinated processes that integrate early clinical suspicion, timely sampling, multidisciplinary interpretation, and therapeutic reassessment. Because the determinants differ among ICU, hematology/HSCT, and neonatal settings, the proposed strategies combine common operational principles with population-specific pathways [31,37,61,94,102,103,173,174].

In this review, bundles are proposed as patient-safety and quality-improvement tools rather than rigid lists of orders. Their purpose is to activate timely diagnostic and therapeutic interventions, define responsibilities, and generate auditable data for AFSP-Dx [31,32,102,134].

### 8.1. Operational Principles Applicable to All Populations

The operational principles outlined below provide a common foundation for reducing diagnostic failure across high-risk populations. They support timely decision-making, coordinated diagnostic processes, and structured reassessment, while allowing adaptation to the specific needs of ICU, hematology/HSCT, and neonatal settings [31,32,94].

1. Do not wait for microbiological confirmation when pretest probability is high and the patient is unstable. In patients with sepsis, rapid deterioration, or profound immunosuppression, the primary objective is to shorten the time to appropriate antifungal therapy. Waiting for absolute microbiological certainty may translate into worse outcomes when clinical suspicion is strong [90].
2. Implement integrated diagnosis within predefined pathways. Decision-making should be supported by the combination of clinical findings, imaging studies, conventional microbiology, biomarkers, molecular assays, rapid tests, and histopathology according to local availability. This approach avoids reliance on a “single test” as a binary decision criterion and allows interpretation to be adjusted according to the host, the probable pathogen, and clinical evolution [20,59,78].
3. Understand source control as both a diagnostic and therapeutic intervention. Catheter removal, drainage of collections, debridement, or surgery not only contribute to infection control, but may also provide critical samples for etiologic confirmation. This is particularly relevant in candidemia, catheter-associated infections, mucormycosis, and other IFIs with tissue-invasive or necrotizing involvement [20,59].
4. Differentiate colonization from infection according to the clinical setting. Interpretation of microbiological isolates must be contextualized. A finding compatible with colonization in the ICU may have different implications in hematology or HSCT settings. In addition, definitions developed for research purposes are not always directly transferable to critically ill patients, neonates, or other non-classical settings [21,33,41,83,147].
5. Reduce “antifungal therapy driven by uncertainty”. Prolonged empiric use without reassessment promotes drug toxicity, avoidable costs, selective pressure, breakthrough events, and selection of less susceptible fungal pathogens. The objective is not to avoid empiric antifungal therapy when indicated, but rather to limit unnecessary duration through systematic review, integrated diagnostics, timely de-escalation, and optimization of antifungal exposure, including TDM when appropriate, especially with triazoles [31,37,52,65,100].
6. Incorporate etiologic identification and antifungal susceptibility testing when they may influence clinical management. In infections caused by non-*albicans* species, suspected *C. auris*, breakthrough infection, emerging molds, *A. fumigatus* with possible azole resistance, or rare pathogens, species-level or species complex-level identification and antifungal susceptibility testing should not be considered ancillary steps. In these scenarios, incomplete diagnosis may perpetuate inactive therapy and delay clinical or epidemiologic control measures [42,52,53,60,75,96,97,125,166].

Together, these principles constitute the foundation upon which diagnostic bundles and population-specific algorithms for ICU, hematology/HSCT, and neonatal settings can be built. Their practical value lies in transforming clinical suspicion into early, measurable, and reviewable actions, while avoiding both therapeutic delay and sustained antifungal overtreatment.

### 8.2. Cross-Cutting Diagnostic-Therapeutic Bundle for Institutional/AFSP Implementation

Reduction in diagnostic failure improves when critical decisions are grouped into simple, sequential, and measurable operational packages. An institutional *bundle* may standardize early responses, reduce clinical variability, and facilitate AFSP auditing [17,31,37,102,174].

Unlike a population-specific diagnostic algorithm, the cross-cutting *bundle* functions as a minimum common institutional response to suspected IFI. Its objective is not to replace clinical judgment or differentiated pathways for ICU, hematology/HSCT, or neonatal settings, but rather to ensure that the initial diagnostic and therapeutic decisions do not depend exclusively on the individual experience of the treating team. In this sense, the bundle acts as a patient-safety tool: it guides the acquisition of useful samples, supports the assessment of pretest probability, facilitates documentation of critical decisions, and establishes an early reassessment point.

Activation of this *bundle* is recommended in any of the following scenarios: sepsis without a clear source, persistent or recurrent fever, respiratory deterioration with imaging findings compatible with IFI, or presence of high baseline risk according to the population being treated.

*Initial bundle: first 0–6 h*

1. Obtain appropriate cultures before initiating antifungal therapy whenever this does not delay critical decisions. These should include blood cultures according to the local protocol and samples from the suspected focus whenever feasible [6,131].
2. Request imaging studies guided by the clinical syndrome. This includes, for example, chest CT in suspected invasive mold disease, as well as echocardiography and ophthalmologic evaluation in candidemia, according to current guidelines and institutional availability [38,40,59,147].
3. Use biomarkers, rapid tests, or molecular methods as complements, not as substitutes for the diagnostic approach. GM, BDG, CrAg, antigens for endemic mycoses, lateral flow assays, PCR, and rapid molecular panels from positive blood cultures may improve the diagnostic approach according to the clinical syndrome, the population being evaluated, and local resources. Nevertheless, they should be interpreted together with clinical judgment, conventional microbiologic investigation, and tissue acquisition when indicated [6,12,45,58].
4. Establish a formal reassessment at 48–72 h from the outset. This step should include explicit criteria for continuing, adjusting, escalating, de-escalating, or discontinuing antifungal therapy by integrating clinical evolution, microbiologic results, biomarkers, imaging studies, and accumulated diagnostic probability [31,37].

*Early bundle: 6–24 h*

1. Initiate appropriate antifungal therapy together with source control when clinical suspicion is high. This measure is particularly relevant in presumed candidemia with septic shock, catheter-associated infection, progressive deterioration compatible with IFI, or high clinical probability despite initially negative cultures [9,41,46,59].
2. Activate an urgent pathway in suspected mucormycosis. This pathway should include tissue acquisition for diagnosis, assessment of anatomic extent, early surgical evaluation, and initiation of an antifungal agent active against Mucorales, without relying on negative or inconclusive serum biomarkers [20].
3. Incorporate early etiologic identification and antifungal susceptibility testing in the setting of suspected resistance, emerging pathogens, or breakthrough infection. This applies particularly to non-*albicans* species, suspected *C. auris*, prior exposure to echinocandins or azoles, progression during treatment, non-*Aspergillus* molds, or suspected azole-resistant *A. fumigatus*. In these scenarios, species-level or species complex-level identification and antifungal susceptibility testing should be integrated early into the diagnostic process whenever available [7,38,65,69,71,73,83,125,127,128,129,147,166].

*Deferred bundle: 48–96 h*

1. Conduct a structured AFSP review. This review should integrate microbiologic results, clinical evolution, imaging studies, biomarkers, prior antifungal exposure, and updated diagnostic probability in order to support a documented therapeutic decision: continue, adjust, escalate, de-escalate, or discontinue antifungal therapy [17,31,174].
2. Avoid delays derived from exclusive reliance on culture. When clinical evolution or baseline risk supports a high suspicion of IFI, waiting only for culture confirmation may prolong clinically relevant delays, particularly in candidemia, mucormycosis, aspergillosis in critically ill patients, and deep mycoses with low circulating fungal burden [12,129].
3. Avoid indefinite empiric antifungal exposure. When comprehensive reassessment at 48–96 h does not support the diagnosis of IFI, antifungal de-escalation or discontinuation should be documented whenever clinically safe. This intervention is key to reducing drug toxicity, pharmacologic interactions, selective pressure, costs, and the risk of breakthrough infections [24,39,169].
4. Optimize antifungal exposure when treatment is continued. In patients receiving triazoles, particularly in settings of prolonged prophylaxis, relevant pharmacologic interactions, suspected drug toxicity, therapeutic failure, or documented IFI, TDM should be considered when indicated. This strategy helps distinguish suboptimal exposure, preventable drug toxicity, and microbiologic or diagnostic failure, while avoiding both unnecessary empiric dose adjustments and unjustified prolongation of therapeutic uncertainty [100,145,164,165].

#### Implications for Diagnostic Bundle Implementation

This *bundle* does not replace population-specific diagnostic algorithms, but rather provides a common institutional foundation upon which pathways for ICU, hematology/HSCT, and neonatal settings may be adapted. Its operational value lies in reducing variability, accelerating critical decisions, and creating an early reassessment point that helps avoid both therapeutic delay and unnecessary antifungal exposure. When integrated into an AFSP-Dx, it also transforms each suspected IFI episode into an opportunity for audit, institutional learning, and continuous improvement [17,31].

### 8.3. Population-Specific Diagnostic Algorithms

The value of clinical algorithms lies in translating general principles into simple, rapid, and auditable action sequences. Because the determinants of diagnostic failure differ among ICU, hematology/HSCT, and neonatal settings, diagnostic pathways must be adapted to each population [17,31,32,37,61,94,102,134].

These algorithms should not be understood as rigid pathways or substitutes for clinical judgment. Their value lies in standardizing initial steps, reducing variability among teams, anticipating critical reassessment points, and avoiding two common errors: delaying treatment in patients with high pretest probability or, conversely, prolonging empiric antifungal therapy when accumulated evidence does not support IFI. In this sense, they function as operational AFSP-Dx tools that integrate diagnosis, therapeutic decision-making, and clinical auditing.

1. Intensive care unit: focus on critical timing and prevention of overtreatment

In the ICU, the algorithm should prioritize diagnostic speed, timely initiation of therapy when suspicion is high, and early reassessment to limit unnecessary exposure. The main challenge is balancing two simultaneous risks: avoiding delays in antifungal therapy in unstable patients with probable IFI while avoiding prolonged treatment in patients whose clinical evolution or diagnostic studies do not support IFI [19,21,24,39,41,45,46,56,59,74,78,83,90,147].

*Step 1. Triggers for suspicion*. Maintain suspicion for IFI in patients with sepsis or shock without a clear source, persistent fever, heavy *Candida* colonization, recent abdominal surgery, PN, CVC, prolonged exposure to broad-spectrum antibiotics, or respiratory deterioration unexplained by other causes.

*Step 2. Immediate diagnostic pathway*. Obtain blood cultures and cultures from the suspected focus, complemented by targeted imaging studies—particularly chest CT in the setting of pulmonary involvement—and additional diagnostic tests according to clinical probability, local availability, and the predominant syndrome.

In the setting of respiratory isolation of *Aspergillus*, a structured clinical algorithm should be applied to differentiate colonization from probable or putative pulmonary aspergillosis in critically ill patients, using ICU-adapted approaches such as AspICU, BM-AspICU, or CAPA-specific criteria when appropriate.

*Step 3. Therapeutic decision-making.* In the presence of septic shock with high suspicion of candidemia, initiate appropriate antifungal therapy together with early source control, including CVC removal or replacement when appropriate.

In suspected invasive mold disease—severe influenza, severe COVID-19, suggestive imaging findings, or respiratory isolation of *Aspergillus* in a compatible clinical context—the diagnostic approach should be intensified and probability-guided treatment considered, while avoiding exclusive reliance on negative serum biomarkers.

*Step 4. Reassessment at 48–72 h.* If no evidence supporting IFI emerges and the patient demonstrates clinical improvement, antifungal de-escalation or discontinuation should be considered in order to reduce unnecessary exposure, a frequent situation in the ICU.

If deterioration persists, the differential diagnosis should be reconsidered and the quality of diagnostic samples, prior antifungal exposure, the possibility of mold disease, resistant species, or an alternative nonfungal infectious etiology should be reassessed.

2. Hematology/HSCT: focus on incomplete diagnosis and uncovered pathogens

In hematology and HSCT settings, the algorithm should aim to avoid partial diagnoses. Antifungal prophylaxis, prolonged neutropenia, and prior azole exposure may mask clinical presentation, modify biomarkers, and favor breakthrough infections caused by uncovered pathogens. Therefore, the pathway should prioritize early imaging, high-quality sampling, reassessment in the absence of response, and active investigation of fungal etiology [6,7,10,20,22,45,48,52,58,60,65,75,77,78,84,96,115,127,135,157,166].

*Step 1. Triggers for suspicion*. Suspect IFI in the presence of prolonged neutropenia, refractory fever, azole prophylaxis, nodular pulmonary lesions or halo sign, facial or orbital pain suggestive of mucormycosis, skin lesions compatible with fusariosis, persistent fungemia, or clinical deterioration despite prior antifungal coverage.

*Step 2. High-yield diagnostic pathway*. Request early chest CT and obtain diagnostic samples—BAL or tissue whenever possible. Biomarkers, including GM, BDG, or others according to clinical suspicion, may support the diagnostic approach, but should be interpreted cautiously in the setting of active mold prophylaxis or prior antifungal exposure.

When suggested by the clinical syndrome, disease evolution, or epidemiology, the differential diagnosis should be expanded to include Mucorales, *Fusarium* spp., *Scedosporium* spp., *L. prolificans*, *Trichosporon* spp., *Saprochaete* spp., *Cryptococcus* spp., or endemic mycoses, with requests for specific testing whenever available.

*Step 3. Therapeutic decision-making that covers the true differential diagnosis*. In suspected mucormycosis—based on compatible clinical or radiologic findings, tissue necrosis, risk factors, or lack of response to azoles—therapy should be promptly switched to an agent active against Mucorales, while prioritizing tissue acquisition and early surgical evaluation when appropriate.

If *Aspergillus* spp. is documented with progression during azole therapy, prior exposure, or compatible epidemiology, azole resistance in *A. fumigatus* should be considered and precise etiologic identification together with antifungal susceptibility testing should be requested whenever feasible.

*Step 4. Etiologic confirmation and antifungal susceptibility testing.* Prioritize species-level or species complex-level identification and antifungal susceptibility testing when they have clinical implications. This approach is particularly relevant in non-*albicans* species, *C. auris*, *N. glabratus*, *P. kudriavzevii*, emerging molds, breakthrough infections, and refractory clinical presentations.

By guiding more precise therapy, this strategy may reduce microbiologic persistence, therapeutic failure, and unnecessary exposure to broad-spectrum antifungal agents.

3. Neonatology: focus on limited blood cultures, CNS, and catheters

In neonatology, the algorithm must recognize that invasive candidiasis may mimic late-onset bacterial sepsis and that blood culture yield is limited by low sample volume. Therefore, suspicion should rely on cumulative risk, clinical evolution, and active investigation of deep-seated foci, with particular attention to the CVC and CNS [14,26,27,28,30,44,64,85,98].

*Step 1. Triggers for suspicion.* Maintain suspicion for invasive candidiasis in premature neonates, particularly ELBW infants, with CVCs, parenteral nutrition, prolonged antibiotic exposure, thrombocytopenia, or persistent LOS without a clear etiology.

*Step 2. Diagnosis*. Obtain blood cultures using the optimal volume permitted by neonatal weight and the local protocol, and investigate secondary foci—urinary tract, CNS, or others according to the clinical context. Because *Candida* meningitis may occur even with negative blood cultures, a negative blood culture should not exclude invasive disease in high-risk neonates with persistent clinical suspicion.

*Step 3. Source control.* Evaluate CVC removal or replacement when candidemia is probable or confirmed, following institutional protocols aligned with international recommendations.

*Step 4. Reassessment*. Document clinical and microbiologic response, and avoid prolonged empiric treatment without defined diagnostic objectives or discontinuation criteria. In the absence of response or persistence of high clinical suspicion, the quality of sampling, the possibility of deep-seated foci, CNS involvement, catheter-associated infection, or an etiology not covered by the initial strategy should be reassessed.

These algorithms show that reducing diagnostic failure requires a common framework—early suspicion, timely sampling, therapeutic decision-making, and reassessment—but applied with different priorities according to the population. This logic supports the translation of these pathways into differentiated clinical figures for ICU, hematology/HSCT, and neonatal settings.

### 8.4. AFSP-Specific Interventions to Reduce Diagnostic Failure and Not Merely Control Antifungal Consumption

AFSPs should not be limited to monitoring antifungal consumption or restricting prescribing. Their greatest potential impact lies in reducing diagnostic failure through processes that improve timeliness, diagnostic precision, and the quality of therapeutic decision-making [31,37,102,134]. The priority institutional interventions to operationalize this approach are summarized in Table 4.

In this context, an AFSP should function as a bridge among clinical suspicion, the laboratory, imaging studies, clinical pharmacy, and treating teams. Its objective is not simply to authorize or deny antifungal agents, but rather to reduce the diagnostic uncertainty that leads both to therapeutic delay and to prolonged empiric use without evidence of IFI. To achieve this, interventions must be practical, auditable, and adapted to populations at highest risk.

These interventions may be organized into institutional modules—candidemia, ICU-associated aspergillosis, mucormycosis, cryptococcosis/endemic mycoses, molecular diagnostics, fungal identification and antifungal susceptibility testing, and TDM—supported by clinical guidelines, population-adapted criteria, and recent AFSP-Dx literature [6,20,31,37,46,57,58,59,60,61,75,78,83,84,100,128,147,157,166].

1. Population-specific order sets with integrated diagnostic bundles. Institutions should develop standardized and differentiated clinical order sets for ICU, hematology/HSCT, and neonatal settings, each incorporating an initial diagnostic bundle. Their purpose is to transform clinical suspicion into timely and traceable diagnostic actions: appropriate cultures, targeted imaging studies, relevant biomarkers, rapid or molecular tests when appropriate, etiologic identification, antifungal susceptibility testing when clinically relevant, and a formal reassessment point [31,37,174].
2. Laboratory alerts with high clinical impact. Critical microbiologic results should trigger immediate notification and predefined response pathways. A priority example is a blood culture positive for yeast, which should be linked to an institutional candidemia checklist: investigation and control of the infectious focus, CVC review, indication for echocardiography or ophthalmologic evaluation according to guidelines, rapid pathogen identification when available, and early antifungal optimization [103]. The same principle should apply to findings with relevant therapeutic or epidemiologic implications, such as suspected *C. auris*, non-*albicans* species with reduced antifungal susceptibility, respiratory isolation of *Aspergillus* in critically ill patients with a compatible clinical context, emerging molds, or identification of rare pathogens requiring confirmation and antifungal susceptibility testing [60,75,83,125,130,131,137,147]. If rapid molecular panels from positive blood cultures are available, their results should be incorporated into the clinical alert, but should not replace antifungal susceptibility testing, source control, or diagnostic reassessment.
3. Mandatory 48–72 h review of all empiric antifungal therapy. Every empirically initiated antifungal agent should undergo a structured review between 48 and 72 h, with a documented decision to continue, discontinue, de-escalate, or escalate treatment according to clinical evolution and available diagnostic results [31,32,61,94,102,134].

This review should integrate updated pretest probability, the quality of obtained samples, microbiologic results, biomarkers, imaging studies, prior antifungal exposure, the possibility of uncovered pathogens, and alternative diagnoses. If treatment is continued, particularly with triazoles, TDM should be considered when indicated in order to optimize exposure, reduce drug toxicity, and avoid misinterpreting pharmacokinetic failure as microbiologic or diagnostic failure [100,145,164,165]. The objective is to avoid both therapeutic delay and indefinite continuation of antifungal therapy driven by diagnostic uncertainty.

4. Audit of antifungal use without documented IFI. The proportion of patients exposed to antifungal agents without subsequent evidence of IFI constitutes a key indicator of diagnostic uncertainty and an opportunity for improvement, particularly in the ICU, where this situation is common [37,81,82].

This indicator should not be understood as a punitive measure, but rather as a signal of diagnostic system performance. Its analysis allows recognition of recurrent failures in timely test activation, the quality and timeliness of sampling, interpretation of results, clinical-microbiologic communication, use of rapid tests, requests for susceptibility profiles when indicated, and antifungal de-escalation decisions.

5. Clinical module for mucormycosis versus aspergillosis. AFSPs may incorporate specific pathways for high-risk scenarios. One particularly relevant example is a clinical alert triggered by lack of response to voriconazole or another azole in the presence of compatible findings, a situation that should activate an urgent reevaluation pathway for mucormycosis, tissue acquisition, early surgical evaluation, and prompt therapeutic adjustment [20,78,84,93,171,173].

This module should emphasize that negative GM and BDG do not exclude mucormycosis, and that clinical or radiologic progression under an *Aspergillus*-directed strategy should be interpreted as a sign of incomplete diagnosis until proven otherwise.

6. Identification, antifungal susceptibility, and alert pathogen module. In addition to syndrome-based modules, AFSPs should define pathogens and clinical scenarios that trigger expanded identification and antifungal susceptibility testing. This includes candidemia caused by non-*albicans* species, suspected or confirmed *C. auris*, *N. glabratus* with prior echinocandin exposure, *P. kudriavzevii*, emerging molds, *A. fumigatus* with possible azole resistance, breakthrough infection, and rare pathogens with unpredictable susceptibility profiles [52,53,60,75,77,125,166].

In these scenarios, species-level or species complex-level identification does not constitute a taxonomic detail, but rather a diagnostic intervention with therapeutic and, in some cases, epidemiologic impact. Integrating this module with laboratory alerts, available rapid methods, and explicit criteria for requesting antifungal susceptibility testing may reduce incomplete diagnoses and prevent initiation of inactive therapy.

#### Implications for AFSP-Dx Implementation

These interventions demonstrate that the modern AFSP not only controls antifungal consumption, but also improves diagnostic precision, shortens critical time intervals, and prevents both undertreatment and overtreatment. Its institutional value depends on transforming repeated clinical decisions into standardized processes: activating suspicion, obtaining useful samples, interpreting results in context, identifying the etiologic agent, documenting the susceptibility profile when relevant, optimizing antifungal exposure when appropriate, and reassessing early. Consequently, AFSP-Dx should be understood as a healthcare quality strategy, not merely as a pharmacologic restriction policy [31,37,61].

## 9. Implications for AFSPs and Proposal for a Diagnostic-Centered Operational Model in ICU, Hematology/HSCT, and Neonatology

AFSPs cannot be reduced to monitoring antifungal consumption, authorizing prescriptions, or containing costs. In the context of IFIs, where diagnostic uncertainty drives both therapeutic delays and unnecessary overtreatment, the operational core of the AFSP should be improving the quality of the diagnostic process [31,37,61].

This approach implies two simultaneous objectives: shortening the time to appropriate antifungal therapy in patients with true infection while, at the same time, limiting prolonged empiric exposure in those who ultimately do not have IFI. Both components are inseparable and represent the essence of a clinically mature AFS.

This approach is consistent with the central pillars of AFSPs: institutional structure, systematic measurement, prospective interventions, auditing, and continuous feedback [17,31,32,37,61,94,102].

In addition, this approach addresses a relevant limitation of daily practice: many classical IFI definitions were developed primarily in populations with hematologic malignancies and HSCT recipients, and are not always directly transferable to critically ill ICU patients or neonatal settings without contextual adjustments [21,41,46,56,135,147]. This lack of transferability may promote diagnostic uncertainty, incomplete classification, or delayed therapeutic decisions when universal algorithms are applied to populations with different epidemiology, biomarkers, and clinical courses.

Therefore, an AFSP-Dx should move beyond rigid models and adopt differentiated strategies according to population, baseline risk, rate of progression, probable fungal pathogen, and available diagnostic tools. In practice, this means developing specific pathways for ICU, hematology/HSCT, and neonatal settings within the same institutional governance framework, with shared indicators, formal reassessment points, and documented decisions regarding initiation, adjustment, de-escalation, or discontinuation of antifungal therapy.

In this model, AFSP-Dx does not act only after antifungal prescribing, but rather from the moment clinical suspicion arises. Its function is to connect patient risk with appropriate sampling, rational selection of diagnostic tests, multidisciplinary interpretation of results, etiologic identification, antifungal susceptibility testing when clinically relevant, and the safest therapeutic decision. When treatment is continued, particularly with triazoles, it should also promote optimization of antifungal exposure through TDM when indicated, avoiding both preventable drug toxicity and unnecessary empiric dose adjustments [100,145,164,165]. Thus, AFSP-Dx ceases to be a reactive intervention focused on antifungal consumption and becomes a proactive strategy for diagnostic quality, therapeutic precision, and continuous improvement.

### 9.1. Proposed Operational Model: Diagnostic-Centered Antifungal Stewardship Program (AFSP-Dx)

A modern AFSP model should be organized around the diagnostic process, not solely around prescribing. The objective of the AFSP-Dx model is to reduce diagnostic failure, shorten the time to appropriate antifungal therapy, and limit unnecessary antifungal exposure through a reproducible institutional structure [17,31,37,61,102,174].

This model is structured around five operational pillars: governance, AFSP-Dx, therapeutic stewardship, surveillance/auditing, and targeted education. Its value lies in connecting clinical suspicion with early diagnostic actions, documented therapeutic decisions, and systematic outcome measurement.

#### 9.1.1. Pillar 1. Governance and Minimum Roles

Every functional program requires a core team with clear and complementary responsibilities. A minimum model should include:

• Infectious diseases specialists, as the clinical leadership of the program;
• Microbiology/mycology, providing diagnostic leadership;
• Clinical pharmacy, with emphasis on PK/PD, pharmacologic interactions, drug toxicity, TDM when appropriate, and cost-effectiveness;
• ICU, hematology/HSCT, and neonatology representatives;
• Nursing, because of its role in sample acquisition, catheter management, and adherence to care *bundles*;
• Hospital epidemiology or quality teams, for surveillance, indicator monitoring, and feedback.

This design aligns with the central elements of AFSPs, which are based on institutional leadership, coordinated interventions, outcome measurement, and continuous feedback [31,61].

#### 9.1.2. Pillar 2. AFSP-Dx: From Clinical Suspicion to Early Reassessment

This pillar seeks to reduce delayed, erroneous, or incomplete diagnosis through population-specific pathways. Its function is to ensure that clinical suspicion is translated early into useful diagnostic actions without delaying critical therapeutic decisions in unstable patients.

Operational interventions include:

• “Suspected IFI” order sets integrating blood cultures, samples from the suspected focus, targeted imaging studies, biomarkers, rapid tests, or molecular methods according to local availability;
• An explicit diagnostic timing rule: obtain samples before antifungal initiation whenever possible; if the patient is unstable, initiate treatment without delay and ensure immediate sampling;
• Clinical–laboratory discussion at 24–48 h to interpret results in context, including colonization versus infection, false-positive or false-negative results, sample quality, the need for additional samples, and consistency with the clinical course;
• Activation of specific pathways in warning scenarios, such as suspected mucormycosis, respiratory isolation of *Aspergillus* in the ICU, candidemia caused by non-*albicans* species, suspected *C. auris*, breakthrough infection, or deterioration during antifungal prophylaxis.

The need for this component is evident in settings such as the ICU, where antifungal use without documented IFI remains frequent and reflects persistent diagnostic uncertainty [17,37,61].

#### 9.1.3. Pillar 3. Therapeutic AFSP: Timely Treatment, Antifungal Optimization, and De-Escalation

The therapeutic objective of AFSP-Dx is twofold: to initiate appropriate antifungal therapy promptly when clinical probability justifies it and to reduce unnecessary exposure when evolving evidence does not support IFI. This pillar connects diagnosis with concrete decisions regarding antifungal selection, dosing, source control, treatment duration, and structured reassessment.

Key interventions include:

• A start smart, then focus strategy: initiate treatment when clinically indicated and reassess at 48–72 h using integrated clinical, microbiologic, radiologic data and pharmacologic information;
• Optimization of antifungal exposure according to renal and hepatic function, pharmacologic interactions, body weight, site of infection, clinical severity, and PK/PD principles;
• TDM when indicated, especially with triazoles, to optimize exposure, reduce drug toxicity, and distinguish pharmacokinetic failure from microbiologic or diagnostic failure;
• Source control as an essential component of treatment;
• Antifungal de-escalation or discontinuation when clinical evolution, diagnostic results, and updated pretest probability do not support IFI;
• Species-level or species complex-level identification and antifungal susceptibility testing when they may modify clinical management.

In candidemia, this includes CVC removal or replacement when indicated and treatment duration guided by microbiologic clearance and complications [103]. It also involves avoiding “wrong pathogen” errors: in aspergillosis, through rational use of biomarkers, imaging studies, respiratory sampling, and fungal identification and antifungal susceptibility testing when azole resistance is suspected [65,81,82,96,166]; and in mucormycosis, through a different strategy based on tissue diagnosis, early surgery, and antifungal agents active against Mucorales [20,112].

#### 9.1.4. Pillar 4. Surveillance, Audit, and Feedback

An AFSP-Dx should be able to demonstrate its clinical and operational impact. Therefore, surveillance should not be limited to intervention volume or the number of recommendations issued, but should also include indicators reflecting diagnostic timeliness, appropriateness of therapy, safe antifungal de-escalation, antifungal resistance, breakthrough events, and clinical outcomes.

Suggested components include:

• A monthly dashboard stratified by population—ICU, hematology/HSCT, and neonatology—with 6–10 priority indicators;
• Audit of empiric antifungal therapy lasting >72 h without subsequent evidence of IFI;
• Audit of time to appropriate therapy in candidemia;
• Audit of time to source control when appropriate;
• Monitoring of etiologic identification, antifungal susceptibility, breakthrough infections, and antifungal resistance;
• Monitoring of indicated, performed, and clinically actionable TDM in patients receiving triazoles, especially in the setting of pharmacologic interactions, suspected drug toxicity, therapeutic failure, or prolonged antifungal prophylaxis;
• Monitoring of antifungal consumption by class and days of therapy per 1000 patient-days;
• Periodic feedback of results to clinical services.

Therapeutic delay in candidemia is consistently associated with higher mortality; therefore, measuring time to appropriate therapy has high clinical relevance [59,81,90,103,169]. However, the AFSP-Dx model should also measure diagnostic precision, safe antifungal de-escalation, reduction of overtreatment, and institutional capacity to detect unsuspected or resistant pathogens [12,31,37,81,147].

#### 9.1.5. Pillar 5. Targeted Education Based on Real-World Problems

Education should address the diagnostic errors and gaps observed in each population rather than being limited to general sessions on antifungal agents. Its value increases when directed toward recurrent clinical scenarios, frequent diagnostic failures, and local indicators.

Priority areas include:

• ICU: differentiation between colonization and infection; sepsis without a clear source; candidemia; ICU-associated aspergillosis; use and interpretation of biomarkers in non-neutropenic patients;
• Hematology/HSCT: IFI during prophylaxis; breakthrough infection; mucormycosis versus aspergillosis; rare molds; azole resistance in *A. fumigatus*;
• Neonatology: candidemia as late-onset neonatal sepsis; limitations of blood cultures; CNS involvement; CVC management;
• Microbiology/mycology: rapid identification, communication of critical results, antifungal susceptibility testing, and pathways for alert pathogens;
• Pharmacy/nursing: pharmacologic interactions, compatibility, TDM when appropriate, drug toxicity, adherence to care *bundles*, and timely sampling.

Targeted education is often more effective than nonspecific general campaigns, particularly when integrated with feedback from real cases, institutional indicators, and concrete opportunities for improvement.

#### 9.1.6. Operational Implications of the AFSP-Dx Model

The AFSP-Dx model shifts AFS from reactive antifungal oversight toward a proactive strategy centered on early diagnosis, therapeutic precision, and continuous improvement. Its purpose is not simply to reduce antifungal consumption, but to improve decision-making: initiating therapy earlier in patients who truly have IFI, discontinuing therapy earlier when evidence does not support IFI, timely identifying resistant or unsuspected pathogens, optimizing antifungal exposure when appropriate, and better documenting where failures occur within the diagnostic process [31,32,37,61,94,102,103,173].

### 9.2. Population-Specific Pathways: How to Operationalize AFSP-Dx Without “a One-Size-Fits-All Approach”

An AFSP-Dx model loses effectiveness when it attempts to operate through a single universal pathway. Pretest probability, rate of progression, test performance, and critical decisions differ substantially among ICU, hematology/HSCT, and neonatal settings. Therefore, AFSP-Dx should be translated into population-specific pathways while maintaining common objectives: earlier recognition of probable IFI, acquisition of useful samples, initiation of appropriate treatment when indicated, and structured reassessment to avoid unnecessary antifungal exposure [17,31,32,37,61,102,174].

1. Intensive care unit (ICU): AFSP-Dx for sepsis/shock without a clear source

In the ICU, the pathway should be activated in the presence of septic shock, persistent sepsis without a clear source, or unexplained clinical deterioration in patients with risk factors for invasive candidiasis or ICU-associated IFI. In this scenario, the main risk is delaying therapy while awaiting microbiologic confirmation in a patient with high pretest probability and rapid progression.

The AFSP-Dx action consists of early activation of the diagnostic *bundle*: blood cultures, active source investigation, targeted imaging when appropriate, CVC evaluation, complementary diagnostic tests according to the clinical syndrome, and timely treatment decisions. If suspicion is high, initiation of appropriate antifungal therapy should be accompanied by early source control, especially in candidemia or catheter-associated infection [9,18,59,83,130,174].

When there is respiratory involvement, respiratory isolation of *Aspergillus*, or severe influenza/COVID-19, the pathway should incorporate criteria adapted to critically ill patients and should not rely exclusively on definitions developed for hematologic patients. In these cases, integrated interpretation of imaging, respiratory microbiology, biomarkers, and clinical evolution is essential to differentiate colonization from probable or putative IA [21,41,46,83,147].

As a process metric, a composite indicator may be used: initiation of appropriate antifungal therapy and source control within 24 h in patients with candidemia and septic shock, when appropriate. This indicator synthesizes the central operational component of ICU management: timely antifungal therapy must be accompanied by effective source control.

The clinical rationale is consistent: therapeutic delay in candidemia is associated with higher mortality, and a strategy based exclusively on waiting for microbiologic confirmation may be harmful in unstable patients [9,18,83].

2. Hematology/HSCT: AFSP-Dx to prevent incomplete diagnosis

In hematologic patients and HSCT recipients, the pathway should be activated in the presence of refractory fever during antifungal prophylaxis, new suggestive pulmonary lesions, clinical signs of angioinvasion, cutaneous lesions compatible with molds, or deterioration without a clear etiology. In this population, the central problem is not only arriving late, but arriving with an incomplete diagnosis or insufficient coverage against the actual pathogen.

AFSP-Dx action should focus on intensifying diagnostic sampling through BAL, tissue acquisition, or other diagnostic procedures when feasible; using biomarkers within the appropriate clinical context; and early reassessment of antifungal coverage when there is no clinical response [6,21,31,45,52,78,81,82,128,135,166].

A critical point is to suspect mucormycosis in the setting of lack of response to azoles, suggestive radiologic progression, or compatible clinical progression. In this context, it is advisable to activate an urgent pathway for tissue acquisition, surgical evaluation, and antifungal therapy active against Mucorales, thereby avoiding prolonged inactive treatment driven by an assumption of aspergillosis [20,112,115].

The pathway should also account for breakthrough infection, rare molds, unsuspected species, azole resistance in *A. fumigatus*, *C. auris*, and other yeasts or molds with unpredictable antifungal susceptibility. In these scenarios, species-level or species complex-level identification and antifungal susceptibility testing are part of the operational diagnosis, not an ancillary step [52,53,60,75,96,125,128,166].

Suggested metrics include the time from a suggestive imaging finding to performance of the diagnostic procedure, the time to an appropriate therapeutic change, etiologic identification at the species or species complex level, and the proportion of episodes with documented antifungal susceptibility when clinically relevant. These indicators more accurately reflect the central challenge in hematology/HSCT: achieving timely and sufficiently detailed etiologic diagnosis.

3. Neonatology: risk-, catheter-, and CNS-based AFSP-Dx

In neonatology, the pathway should be activated in cases of persistent LOS in preterm infants or ELBW neonates with CVCs, PN, prolonged antibiotic exposure, or unexplained thrombocytopenia. In this population, diagnostic failure is associated with low clinical specificity, limited blood culture volume, and the possibility of deep involvement, including CNS involvement.

AFSP-Dx actions include optimized blood culture collection using the volume recommended by the local protocol, active investigation of secondary foci, and early decisions regarding CVC removal or replacement when candidemia is probable or confirmed [17,31,61].

Suggested metrics include time to CVC intervention in probable or confirmed candidemia, the proportion of neonates evaluated for secondary foci—including CNS involvement according to the clinical presentation and local protocol—and time to initiation of appropriate antifungal therapy. The clinical rationale is straightforward: in ELBW neonates, invasive candidiasis is associated with severe outcomes, including mortality and long-term neurologic impairment; therefore, reducing diagnostic and therapeutic delays is particularly critical [26,30,85,98].

#### Operational Implications of Population-Specific Pathways

These pathways show that a single institutional program can operate with shared objectives while requiring distinct clinical decisions depending on the population served. In the ICU, the focus is on timely therapy, source control, and early antifungal de-escalation; in hematology/HSCT, etiologic depth, detection of pathogens outside routine coverage, and recognition of antifungal resistance; and in neonatology, early suspicion, optimized blood cultures, catheter management, and investigation of deep-seated involvement. This differentiation prevents AFSP-Dx from becoming a generic policy and instead transforms it into a practical, auditable clinical tool aligned with real-world risk.

### 9.3. Minimum Dataset and AFSP Indicators for Reporting, Adjusted by Population

Measurement of diagnostic failure should capture diagnostic timeliness, therapeutic appropriateness, source control, completeness of microbiological characterization, antifungal exposure, and clinical outcomes. Because these priorities differ across ICU, hematology/HSCT, and neonatal settings, the proposed framework combines a core institutional set with population-specific indicators [17,21,30,31,37,60,61,65,75,78,81,83,100,147,165]. The core institutional indicators are summarized in Table 3 and further specified below.

#### 9.3.1. Core Indicators: All Services

The basic indicators recommended across all care settings should function as a common language between services. Their purpose is not to create an additional administrative burden, but rather to identify points in the process where diagnostic timeliness, microbiologic precision, or the ability to perform antifungal de-escalation is lost.

Within this framework, the minimum core may include:

• Time to documented suspicion: hours from onset of the clinical syndrome to the first documentation of IFI within the differential diagnosis;
• Time to specimen collection before antifungal therapy*:* proportion of episodes with blood cultures or other useful diagnostic samples obtained before treatment initiation, provided this does not delay critical decisions;
• Time to appropriate antifungal therapy*:* hours until initiation of an active antifungal agent at an appropriate dose against the pathogen ultimately identified;
• Time to etiologic identification*:* interval until identification at the species or species complex level when clinically relevant;
• Time to antifungal susceptibility results: interval until antifungal susceptibility testing becomes available when indicated by species, prior antifungal exposure, breakthrough infection, therapeutic failure, or suspected resistance;
• Time to source control: interval until CVC removal, drainage, or surgery when indicated;
• Empiric antifungal therapy >72 h without documented IFI: proportion of patients who continue antifungal therapy beyond 72 h without subsequent evidence of IFI, as an indicator of persistent diagnostic uncertainty and opportunity for reassessment;
• DOT per 1000 patient-days, stratified by empiric, targeted, and prophylactic use;
• Indicated TDM performed: proportion of patients treated with triazoles in whom TDM was indicated and effectively performed, particularly in the setting of high pharmacokinetic variability, relevant drug interactions, suspected drug toxicity, prolonged prophylaxis, or therapeutic failure;
• Breakthrough events*:* number of IFIs occurring during antifungal prophylaxis or therapy relative to the total number of exposed patients;
• Clinically relevant antifungal resistance*:* rate of echinocandin resistance in *N. glabratus*, azole resistance in *A. fumigatus*, or identification of *C. auris* with documented susceptibility profile, when local measurement is available [36,125,139,166];
• 30-day mortality in confirmed IFI, ideally severity-adjusted whenever possible.

#### 9.3.2. Population-Specific Indicators

In addition to the common core indicators, some metrics should be adapted to the population served. This differentiation avoids comparing non-equivalent clinical processes and allows each service to evaluate the factors that determine diagnostic failure within its own care setting.

• ICU: proportion of patients with candidemia and shock who receive appropriate antifungal therapy plus source control within 24 h; time from clinical deterioration to specimen collection; time from blood culture positivity to rapid identification when molecular panels from positive blood cultures are used; and rate of antifungal de-escalation at 72–96 h when comprehensive evaluation does not support IFI [9,18,21,39,90].
• Hematology/HSCT: proportion of suspected episodes with high-quality sampling—BAL, tissue, or equivalent; proportion of cases receiving antifungal prophylaxis with AFSP review within the first 48–72 h; time to identification at the species or species complex level; documented antifungal susceptibility testing when clinically relevant; and proportion of patients receiving triazole treatment or prophylaxis in whom TDM is performed when indicated [11,19,45,52,78,84,93,127,135,165,166].
• Neonatology: time to CVC intervention in probable or confirmed candidemia; proportion of neonates undergoing evaluation for secondary foci according to the institutional protocol, including CNS assessment when indicated; blood volume per blood culture when recorded; time to initiation of appropriate antifungal therapy; and time to species identification and antifungal susceptibility testing when an isolate is available [30,85].

#### 9.3.3. Operational Utility

This minimum set allows the development of comparable measurements across services, identification of diagnostic bottlenecks, and linkage of AFSP actions to concrete clinical outcomes. Its value lies not only in describing antifungal consumption or mortality, but also in showing where the diagnostic process fails and at which point an intervention may modify the outcome.

In practice, these indicators may be incorporated progressively. An initial phase may be limited to critical time points—suspicion, sampling, therapy, and source control—and subsequently add metrics related to etiologic identification, antifungal susceptibility, TDM, antifungal de-escalation, antifungal resistance, breakthrough events, and adjusted clinical outcomes. This stepwise implementation facilitates sustainability, reduces operational burden, and allows AFSP-Dx to demonstrate clinical impact before expanding its scope.

### 9.4. How to Implement Without Friction: A 90-Day Pathway

Implementation of an AFSP-Dx model may fail when approached as a complex reform, excessively bureaucratic initiative, or process dependent on extraordinary resources. In practice, the best results are usually achieved through short cycles, simple goals, and progressive expansion. An initial 90-day pathway allows demonstration of early value, correction of operational barriers, and generation of institutional engagement [17,31,32,37,61,102].

The purpose of this pathway is not to build a complete and perfect program from the outset, but rather to establish a minimum viable version that is clinically useful and measurable. To achieve this, it is advisable to begin with a limited number of indicators, a clear communication workflow, and a pilot population in which the impact can be rapidly observed.

#### 9.4.1. Weeks 1–2: Minimum Viable Design

During the initial phase, the essential components of the program should be defined. This stage should focus on specific decisions applicable to real clinical workflows, avoiding the design of an overly broad model before it has been tested.

The initial steps include:

• Establishing population-specific clinical activation triggers—ICU, hematology/HSCT, and neonatology;
• Approving institutional *order sets* with an incorporated diagnostic bundle;
• Defining responsibility for AFSP review at 48–72 h;
• Agreeing on initial indicators and data sources;
• Formalizing communication channels between infectious diseases, microbiology/mycology, clinical pharmacy, and clinical services;
• Defining alert criteria for high-impact pathogens or scenarios, such as candidemia, suspected *C. auris*, mucormycosis, ICU-associated aspergillosis, breakthrough infection, or clinical deterioration during antifungal prophylaxis;
• Establishing criteria to trigger expanded identification, antifungal susceptibility testing, rapid panels from positive blood cultures, or TDM, according to institutional availability and clinical relevance.

The goal is not initial perfection, but rather the launch of a functional, understandable, and applicable version integrated into routine clinical practice.

#### 9.4.2. Weeks 3–6: Controlled Pilot in the ICU

The ICU is often an appropriate starting point because of patient volume, frequent antifungal use, and the high clinical impact of early decisions. In addition, it allows rapid evaluation of two central problems in diagnostic failure: therapeutic delay in unstable patients and empiric antifungal overtreatment when the accumulated evidence does not support IFI.

Suggested actions include:

• Conducting a prospective audit of 20–30 episodes of antifungal therapy initiation;
• Measuring critical time points: clinical suspicion, specimen collection, therapy initiation, and source control;
• Reviewing cases with overtreatment, diagnostic delay, or absence of reassessment at 48–72 h;
• Evaluating the time from positive blood culture to rapid identification when molecular panels are used;
• Adjusting the *bundle* according to the actual barriers identified;
• Producing a monthly summary with core indicators;
• Providing brief feedback to the ICU team focused on modifiable decisions: specimen collection, source control, continuation, or discontinuation of antifungal therapy.

This phase allows demonstration of early results and helps establish institutional legitimacy [21,24,39,41,59,174].

#### 9.4.3. Weeks 7–12: Progressive Expansion

Once the pilot has stabilized, the model can be scaled to hematology/HSCT and neonatology with contextual adaptation. Expansion should not consist of copying the ICU pathway, but rather preserving the same operational framework—suspicion, sampling, therapeutic decision-making, and reassessment—while adapting it to the predominant problems of each population.

Suggested actions include:

• Incorporating population-specific pathways;
• Developing local leaders within each service;
• Implementing brief monthly feedback by unit—2–3 slides including indicators, trends, and index cases;
• Comparing performance before and after implementation;
• Prioritizing one or two improvement objectives per service;
• Adapting indicators to each population: etiologic depth, antifungal susceptibility, and TDM in hematology/HSCT; optimized blood cultures, CVC management, and evaluation of deep-seated foci in neonatology.

#### 9.4.4. Principles to Reduce Operational Friction

Implementation tends to be more effective when AFSP-Dx is incorporated into the routine clinical care workflow rather than operating as a parallel administrative layer.

To achieve this, several practical principles should be maintained:

• Integrating into routine clinical workflows without creating unnecessary parallel pathways;
• Leveraging already available data before requiring new platforms or databases;
• Providing brief, frequent, and actionable feedback;
• Demonstrating early and visible clinical benefits;
• Recognizing operational differences between services rather than imposing a single model;
• Preventing AFSP-Dx from being perceived as an administrative barrier by positioning it as a clinical support tool for complex decisions.

#### 9.4.5. Operational Feasibility and Local Adaptation

Within a 90-day period, it is possible to move from a theoretical AFS concept to a functional program with basic indicators, auditable processes, and the first demonstrable improvements. This approach promotes sustainability by reducing institutional resistance, facilitating early adjustments, and positioning AFSP-Dx as a practical healthcare quality tool rather than an additional administrative policy.

### 9.5. Expected Outcome: What Should Change if the Model Works

The value of an AFSP-Dx model should not be measured by the number of meetings held or restrictions issued, but rather by verifiable clinical changes in diagnostic timeliness, therapeutic precision, and healthcare outcomes. If the model works, progressive improvements in key indicators should be observed during the first months of implementation [31,37,102].

Interpretation of these results should be gradual. Some improvements—such as increased documentation of clinical suspicion, earlier specimen collection, activation of specific pathways, or systematic reassessment at 48–72 h—may become evident rapidly. Others, such as reductions in mortality, antifungal resistance, breakthrough events, or hospital length of stay, require longer observation periods and adjustment for clinical severity.

1. Reduction in time to appropriate therapy in candidemia. One of the expected early outcomes is shortening the interval between clinical suspicion and initiation of appropriate antifungal therapy, particularly in candidemia and invasive candidiasis associated with septic shock. This indicator is clinically relevant because therapeutic delay has been associated with increased mortality [18,24,39].
2. Reduction in prolonged empiric antifungal exposure without documented IFI in the ICU. Systematic reassessment at 48–72 h and integration of clinical, microbiologic, imaging, and pharmacologic data should reduce the proportion of critically ill patients who continue empiric antifungal therapy without subsequent evidence of IFI [12,17,37,81,82].
3. Greater etiologic confirmation in hematology/HSCT. In this population, a more proactive diagnostic system should increase acquisition of high-quality samples, improve identification at the species or species complex level, promote antifungal susceptibility testing when clinically relevant, and reduce both incomplete diagnoses and prolonged treatments without etiologic confirmation [6,12,38,43,77,128].
4. Reduced delay and lower variability in neonatal candidemia. In neonatology, standardization of clinical pathways should reduce variability between treating teams and improve the timeliness of CVC intervention, investigation of metastatic foci, and therapeutic appropriateness in cases of probable or confirmed candidemia [6,30,85].
5. Improved detection of alert pathogens and breakthrough events. A functional AFSP-Dx should promote early detection of pathogens with therapeutic or epidemiologic implications, such as non-*albicans* species, *C. auris*, emerging molds, Mucorales, and *A. fumigatus* with possible azole resistance. It should also facilitate timely recognition of breakthrough IFIs during antifungal prophylaxis or therapy, preventing continuation of regimens inactive against the causative pathogen [65,70,125,129,166].
6. Optimization of antifungal exposure when appropriate. In patients treated with triazoles, particularly in hematology/HSCT or during prolonged treatment courses, the model should increase the proportion of indicated and effectively performed TDM, as well as documentation of adjustments derived from suboptimal or potentially toxic concentrations. This outcome should be understood not only as a pharmacologic metric, but also as an indicator of therapeutic precision and patient safety [100,145,165].
7. Additional institutional benefits. In addition to direct clinical outcomes, a functional AFSP-Dx may generate important institutional benefits, including:
  • Reduced variability across services;
  • Improved coordination between clinical services, laboratory, imaging, pathology, and pharmacy;
  • More rational use of biomarkers, rapid tests, molecular panels from positive blood cultures, and molecular methods;
  • Progressive reduction in unnecessary antifungal exposure;
  • Greater institutional capacity to identify failures within the diagnostic system;
  • Improved documentation of decisions regarding initiation, adjustment, de-escalation, or discontinuation of antifungal therapy.

#### Implications for Outcome Measurement and Continuous Improvement

If improvements are not observed in diagnostic timelines, etiologic precision, or the quality of therapeutic decisions, the program is likely capturing administrative activity rather than real clinical impact. Therefore, AFSP-Dx monitoring should focus on process indicators and clinical outcomes, not solely on the volume of interventions or reduction in antifungal use.

**Figure 5:**
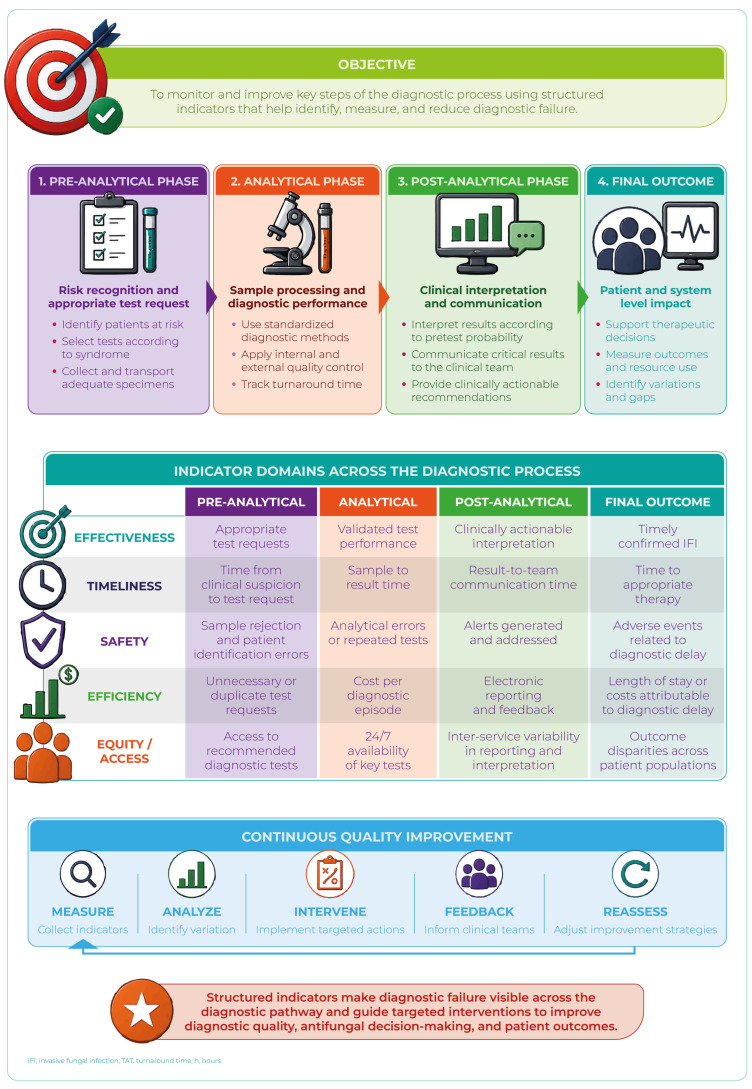
Indicator domains across the diagnostic process to reduce diagnostic failure in invasive fungal infections. This figure organizes measurable indicators across the main phases of the diagnostic pathway, from pre-analytical recognition and test requesting to analytical performance, post-analytical interpretation, and final patient- or system-level outcomes. The proposed domains—effectiveness, timeliness, safety, efficiency, and equity/access—are intended to make diagnostic failure visible as a process problem rather than as an isolated test limitation. Used within AFSP-Dx or institutional quality-improvement programs, these indicators can support local audit, identify delays or variability across services, guide targeted interventions, and monitor whether diagnostic stewardship improves antifungal decisions and patient outcomes.

The proposed operational structure for implementing this institutional model and its main strategic components is summarized in Figure 5.

## 10. Evidence Gaps, Research Priorities, and the AFSP-Dx Agenda to Reduce Diagnostic Failure in IFIs

Despite advances in biomarkers, advanced imaging, rapid testing, and molecular methods, diagnostic failure in IFIs remains a clinically relevant problem. This gap depends not only on technological availability, but also on three persistent limitations: poorly standardized diagnostic definitions across populations, uneven evidence according to the clinical setting, and limited integration of tests into reproducible diagnostic care algorithms [17,31,37,102].

Classical IFI definitions have been fundamental for research, but they were developed primarily in hematology and HSCT patients; therefore, their direct extrapolation to the ICU, neonatology, or other non-classical settings may generate diagnostic uncertainty and methodological heterogeneity [21,41,83,147]. In addition, although the evidence is relatively robust for IA in hematology, it remains limited in non-neutropenic critically ill patients, neonates, breakthrough IFIs, rare/emerging fungi, cryptococcosis outside classical contexts, and endemic mycoses in epidemiologically unrecognized settings [9,53,57,58,75,85]. In practice, even when tests are available, they are frequently not ordered in a timely manner, not interpreted within context, or do not modify therapeutic decisions in a structured way [7,11,65,81,82,129].

Therefore, the future agenda should not focus solely on developing new diagnostic tests, but also on demonstrating how to better use currently available tools, in which patients, with which diagnostic combinations, at which reassessment points, and with what real clinical impact.

### 10.1. Cross-Cutting Gaps

There are three common gaps that limit comparisons between studies and implementation of AFSP-Dx programs.

First, a standardized operational definition of “diagnostic failure” is needed to differentiate delayed diagnosis, incorrect diagnosis, and incomplete diagnosis. This taxonomy should be harmonized with frameworks such as EORTC/MSGERC, while being adapted to real-world practice settings, especially the ICU, neonatology, and non-classical populations [21,26,83,98,147].

Second, studies should incorporate comparable process and outcome endpoints: time to documented suspicion, time to useful sampling, time to appropriate antifungal therapy, time to source control, proportion of empiric therapy >72 h without documented IFI, 30-day mortality, DOT per 1000 patient-days, breakthrough events, identification at the species or species complex level, and clinically relevant antifungal resistance [31,37,61]. Without common metrics, even promising interventions become difficult to interpret or replicate.

Third, clinical evidence regarding the impact of integrated diagnostic bundles remains limited. The literature has extensively evaluated the performance of individual diagnostic tests—such as GM, BDG, CrAg, antigens for endemic mycoses, lateral flow rapid tests, or PCR—but studies demonstrating whether combined algorithms reduce mortality, shorten critical time points, decrease unnecessary antifungal use, improve antifungal de-escalation, or reduce costs under real-world practice conditions are less common [13,81,82,109,121,123,136,174].

The next advance in the field will probably not be an isolated test, but rather validation of reproducible diagnostic pathways that combine existing tools in a rational, measurable manner adapted to the host, the probable pathogen, and the care setting.

### 10.2. Population-Specific Priorities

Research priorities must be adapted to each population because the ICU, hematology/HSCT, and neonatology do not share the same pretest probability, progression rate, or diagnostic performance.

#### 10.2.1. Research Priorities in the Intensive Care Unit

In critically ill patients, research priorities include validating diagnostic strategies for invasive mold disease in non-neutropenic patients, better defining the performance and interpretation of GM, BDG, lateral flow rapid tests, and PCR in the context of colonization or critical inflammation, and evaluating interventions capable of reducing empiric antifungal use without documented IFI while preserving clinical safety [21,41,46,56,135,147].

The central questions are which *bundle* most effectively reduces “antifungal use driven by uncertainty”; which syndromic trigger—sepsis without a source, respiratory deterioration, or shock—best activates intensive diagnostic evaluation; which combination of tests provides the greatest incremental value in real-world ICU practice; and how to integrate adapted criteria, such as AspICU, BM-AspICU, CAPA, or FUNDICU, into auditable clinical decisions.

#### 10.2.2. Research Priorities in Hematology and HSCT

In hematology and HSCT patients, the agenda should focus on diagnostic algorithms applicable during anti-mold prophylaxis, early differentiation between aspergillosis and mucormycosis, detection of breakthrough infection, identification of rare or emerging fungi, and systematic incorporation of identification at the species- or species complex-level and antifungal susceptibility testing when clinically relevant [20,52,60,65,75,78,111].

In this population, combined approaches integrating biomarkers, PCR, imaging studies, culture, histopathology, and antifungal resistance detection are particularly relevant, since no single tool provides sufficient performance across all scenarios of profound immunosuppression or active antifungal prophylaxis [6,12,81,82].

Priority questions include which strategy reduces incomplete diagnosis of Mucorales, azole-resistant *A. fumigatus*, *C. auris*, and rare molds without increasing drug toxicity or costs; how to integrate PCR, NGS, rapid tests, culture, antifungal susceptibility testing, and pathology when biopsy entails high risk; which patients truly benefit from early invasive diagnostic procedures; and how to incorporate TDM into treatment or prophylaxis algorithms with triazoles to distinguish suboptimal exposure, preventable drug toxicity, and true microbiologic failure [100,128,145,157,165,166].

#### 10.2.3. Research Priorities in Neonatology

In neonates, particularly preterm and ELBW infants, studies are needed to validate BDG, PCR, and other non-culture-based tests; optimize blood culture volume and number; and define standards for investigation of secondary foci, CNS evaluation, and CVC management within care *bundles*. These challenges have also been highlighted in recent reviews on neonatal invasive candidiasis [98].

Priority questions include which combination of tests reduces diagnostic delay without increasing overtreatment; which AFSP-Dx indicators best predict death or neurodevelopmental sequelae; and which CVC intervention strategy provides the greatest net benefit according to the clinical timing.

### 10.3. AFSP-Dx Agenda: Recommended Study Designs

Future studies should evaluate complete diagnostic-therapeutic strategies rather than the isolated performance of individual tests. In this regard, three study designs are particularly relevant.

Recent reviews on AFSP in IFIs indicate that these interventions may improve care processes and optimize antifungal use; however, heterogeneity in reported study designs, indicators, and clinical outcomes limits comparisons between studies and underscores the need for standardized metrics [134].

Pragmatic cluster studies or *stepped-wedge* designs would be particularly useful for evaluating integrated diagnostic bundles with AFSP review at 48–72 h, audit, and feedback. Outcomes should include time to appropriate therapy, DOT, proportion of empiric treatment without documented IFI, safe antifungal de-escalation, 30-day mortality, and hospital costs [17,31,37].

### 10.4. Minimum Reporting Set for AFSP-Dx Studies

To improve comparability across studies, hospitals, and populations, future publications should incorporate a minimum set of standardized variables organized into four main domains.

• Process variables*:* time to documented suspicion, specimen collection, initiation of appropriate therapy, and source control; adherence to the diagnostic-therapeutic bundle; and documented reassessment at 48–72 h.
• Antifungal use variables*:* DOT per 1000 patient-days, proportion of empiric therapy >72 h without documented IFI, rate of antifungal de-escalation at 72–96 h, safe discontinuation after diagnostic reassessment, and proportion of treatments with indicated, performed, and clinically actionable TDM, particularly with triazoles.
• Clinical variables: 30-day mortality, ICU and hospital length of stay, recurrence or persistence of infection, antifungal-attributable drug toxicity, and need for invasive procedures or rescue therapies.
• Microbiologic variables: etiologic identification at the species or species complex level, antifungal susceptibility testing when clinically impactful, relevant antifungal resistance, breakthrough events during antifungal prophylaxis or therapy, and detection of alert pathogens, including *C. auris*, echinocandin-resistant *N. glabratus*, azole-resistant *A. fumigatus*, Mucorales, and rare or emerging fungi.

Adoption of a standardized minimum set of variables would facilitate future evidence syntheses, improve comparability across centers and populations, and allow more rigorous evaluation of the real impact of AFSP-Dx interventions [31,37].

## 11. Conclusions

Diagnostic failure in IFIs continues to be an important—and frequently underestimated—cause of mortality, morbidity, and inefficient use of healthcare resources. Its impact depends not only on the absence of diagnostic tests, but also on cumulative failures throughout the care process: delayed suspicion, inadequate sampling, decontextualized interpretation of results, incomplete diagnosis, and delayed therapeutic decisions.

Throughout this review, it has been shown that diagnostic failure does not adopt a single form. In the ICU, syndromic uncertainty, empiric overtreatment, and the need for decisions within short time windows predominate. In hematology and HSCT patients, prior antifungal prophylaxis, breakthrough infections, pathogens outside routine coverage, antifungal resistance, and difficulty obtaining complete etiologic confirmation are particularly prominent. In neonates, similarity to late-onset bacterial sepsis, limitations of blood cultures, the role of the catheter, and the risk of disseminated involvement require specific and early strategies.

The central conclusion is that no isolated test will solve this problem on its own. Reducing diagnostic failure requires integrated models that combine pretest probability, timely sampling, conventional microbiology, biomarkers, rapid tests, molecular panels from positive blood cultures, imaging studies, histopathology, molecular methods when available, identification at the species or species complex level, antifungal susceptibility testing when clinically relevant, and serial clinical reassessment. When treatment is continued, particularly with triazoles, optimization of exposure through TDM may also contribute to improving safety and therapeutic precision.

In this context, AFSPs should evolve from models focused primarily on antifungal consumption toward diagnostic-centered strategies. An AFSP-Dx model provides an operational framework to integrate clinical suspicion, diagnostic testing, therapeutic decisions, source control, audit, and continuous feedback. Its value lies not only in restricting antifungal use, but also in improving the quality of clinical decisions and making measurable the points at which the diagnostic process fails.

In summary, reducing diagnostic failure likely represents one of the interventions with the greatest potential to improve clinical outcomes in IFIs over the next decade. Achieving this will require progress toward population-adapted algorithms, auditable indicators, pragmatic implementation studies, and closer collaboration among clinicians, microbiologists, mycologists, pharmacists, radiologists, pathologists, and healthcare quality teams.

## Figures and Tables

**Table 1:** Operational framework of diagnostic failure in IFIs: categories, consequences, and auditable indicators [20,21,31,37,55,56,57,58].

Category	Practical Definition	Common Clinical Example	Main Consequence	Suggested AFSP-Dx Metric	Suggested Corrective Intervention
Delayed diagnosis	The IFI is recognized after the optimal therapeutic window, when clinical progression, organ dysfunction, or established tissue damage is already present.	ICU patient with septic shock and candidemia in whom antifungal therapy is initiated only after blood culture positivity.	Increased mortality, progression of organ dysfunction, prolonged hospitalization, and lower probability of response.	Time from onset of the clinical syndrome to documented suspicion, specimen collection, and appropriate antifungal therapy.	Activate an early bundle in cases of sepsis/shock without a clear source; obtain useful specimens without delaying therapy in unstable patients with high pretest probability.
Incorrect diagnosis	The IFI is interpreted as another entity, or a finding compatible with colonization or contamination is treated as invasive disease.	Respiratory isolation of *Candida* incorrectly interpreted as pneumonia; respiratory isolation of *Aspergillus* in the ICU classified as colonization without structured evaluation.	Undertreatment when true IFI is not recognized, or overtreatment when colonization is treated as invasive disease.	Proportion of empiric antifungal therapies without documented IFI; proportion of critical isolates undergoing clinical-microbiologic review.	Implement diagnostic algorithms for colonization versus infection; establish clinical–laboratory discussion in cases of critical or discordant results.
Incomplete diagnosis	The IFI is recognized, but the causative agent, anatomic extent, antifungal susceptibility profile, coinfection, or antifungal resistance are not sufficiently characterized.	Mold infection treated empirically without identification at the species or species complex level or evaluation of extent; candidemia caused by a non-*albicans* species without antifungal susceptibility testing when clinically relevant.	Inactive or suboptimal therapy, delay in switching antifungal class, missed coinfection, or incomplete source control.	Time to identification at the species or species complex level; proportion of cases with indicated and performed antifungal susceptibility testing; time to evaluation of anatomic extent.	Prioritize advanced identification, antifungal susceptibility testing when the result may modify clinical management, focus/tissue sampling, and reassessment in the absence of response.
Diagnostic limitation	Objective absence of tests, infrastructure, timely access, or expertise required to confirm or characterize the IFI.	Unavailability of GM, BDG, CrAg, endemic mycosis antigens, fungal PCR, CT, specialized histopathology, antifungal susceptibility testing, or mycology support.	Structural delay, reliance on presumptive diagnosis, and greater therapeutic uncertainty.	Diagnostic turnaround time; proportion of unavailable tests or tests referred externally; time from test request to result.	Define referral pathways, prioritize critical tests, establish agreements with external laboratories, and apply pragmatic algorithms according to available resources.
Process failure	Diagnostic tools exist, but they are requested late, specimens are inadequate, or results are interpreted without clinical context.	BDG requested after several days of antifungal therapy; biopsy sent only for histopathology without a sterile sample for culture/PCR; CrAg not requested in compatible subacute meningitis.	Avoidable delay, loss of diagnostic yield, and prolonged therapeutic uncertainty.	Time to first useful diagnostic action; proportion of adequate specimens; adherence to the bundle; documented AFSP-Dx review at 48–72 h.	Incorporate order sets, sampling checklists, clinical–laboratory communication, and structured AFSP-Dx review at 48–72 h.

Note: This table proposes an operational framework to identify actionable points within the diagnostic process; it does not replace established definitions of proven, probable, or possible IFI. AFSP-Dx, diagnostic-centered antifungal stewardship program; BDG, 1,3-β-D-glucan; CrAg, cryptococcal antigen; CT, computed tomography; GM, galactomannan; ICU, intensive care unit; IFI, invasive fungal infection; PCR, polymerase chain reaction.

**Table 2:** Integrated diagnostic approach to reduce diagnostic failure according to clinical setting [15,19,20,30,31,37,41,46,57,58,59,74,78,83,84,85,86,87,88,89].

Population/Clinical Setting	Diagnostic Objective	Initial Tests	Complementary Tools	Frequent Error	Recommended AFSP-Dx Action
ICU with sepsis or shock without a clear source	Recognize early candidemia/IC and avoid waiting for delayed confirmation.	Blood cultures; cultures from the suspected source; CVC evaluation and active search for a source.	BDG, when available, may contribute to rule-out strategies in selected ICU patients when interpreted together with pretest probability, clinical evolution, and other diagnostic findings; it should not be used as a standalone test to confirm or exclude invasive candidiasis. Chest or abdominal CT according to the clinical syndrome; ultrasound, drainage, or targeted imaging when a deep focus is suspected; T2*Candida* if available and locally validated; and rapid molecular panels from positive blood cultures when applicable.	Waiting exclusively for culture positivity or attributing the clinical picture solely to bacterial sepsis.	Activate the initial bundle; initiate antifungal therapy if pretest probability is high and the patient is unstable; reassess at 48–72 h to continue, adjust, or discontinue treatment.
ICU with respiratory deterioration	Differentiate colonization from invasive infection and recognize ICU-associated aspergillosis.	Respiratory sample; culture; direct examination when available; BAL if feasible.	BAL GM; chest CT; serum GM with caution; targeted PCR or rapid LFA/LFD tests when available.	Assuming only bacterial VAP or interpreting respiratory *Aspergillus* as colonization without structured evaluation.	Apply criteria adapted to critically ill patients—AspICU, BM-AspICU, or CAPA according to the context—and integrate imaging studies, respiratory microbiology, and clinical evolution.
Hematology/HSCT with persistent fever or pulmonary lesion	Avoid incomplete diagnosis during antifungal prophylaxis and detect breakthrough IFIs or pathogens outside routine coverage.	Blood cultures; early chest CT; clinical evaluation of skin, paranasal sinuses, and deep-seated foci.	Serial serum GM; BAL GM/PCR; BDG according to context; targeted PCR, panfungal PCR/sequencing, or biopsy if feasible.	False reassurance due to negative biomarkers during active anti-mold prophylaxis or delay in obtaining deep specimens.	Intensify the diagnostic workup despite negative screening if high suspicion persists; prioritize BAL/tissue sampling, identification at the species or species complex level, and antifungal susceptibility testing when relevant.
Suspected mucormycosis	Confirm tissue invasion and avoid inactive treatment against Mucorales.	Urgent imaging of the affected site; surgical evaluation; tissue biopsy when possible.	Histopathology; tissue culture; PCR or sequencing if available; evaluation of anatomic extent.	Requesting only serum biomarkers or treating as aspergillosis without reassessment in the setting of progression.	Activate an urgent tissue-based pathway; do not exclude mucormycosis because of negative GM/BDG; initiate therapy active against Mucorales if suspicion is high.
Subacute neurologic or pulmonary syndrome, or disseminated disease in an immunocompromised patient	Do not overlook cryptococcosis or endemic mycoses when suggested by the syndrome, immunosuppression, or epidemiology.	Blood cultures/targeted cultures; CSF if neurologic involvement is suspected; imaging according to the syndrome; epidemiologic history.	Serum/CSF CrAg; *Histoplasma* antigen in urine/serum; serology or PCR according to suspicion and availability.	Applying a diagnostic algorithm focused only on *Candida/Aspergillus* or assuming tuberculosis/neoplasia without specific mycologic testing.	Incorporate clinical-epidemiologic triggers for CrAg and endemic mycoses; integrate epidemiology, clinical syndrome, deep specimens, and specific testing.
Late-onset neonatal sepsis (LOS)	Recognize early invasive candidiasis despite nonspecific clinical presentation and limited blood culture yield.	Blood culture with optimized volume according to weight and institutional protocol; urine culture; CVC evaluation.	CNS, urinary, ocular, or cardiac evaluation according to the clinical presentation/protocol; ultrasound or targeted imaging if a deep focus is suspected.	Assuming only late-onset bacterial sepsis or considering a negative blood culture reassuring in a high-risk neonate.	Activate a neonatal candidemia-oriented bundle; initiate antifungal therapy if clinical probability is high; intervene on the CVC and reassess clinical/microbiologic response.

Note: This table summarizes guiding diagnostic approaches according to the clinical setting; proposed actions should be adapted to individual risk, local epidemiology, availability of diagnostic resources, and institutional protocols. AFSP-Dx, diagnostic-centered antifungal stewardship program; BAL, bronchoalveolar lavage; BDG, 1,3-β-D-glucan; CAPA, COVID-19-associated pulmonary aspergillosis; CNS, central nervous system; CrAg, cryptococcal antigen; CSF, cerebrospinal fluid; CT, computed tomography; CVC, central venous catheter; GM, galactomannan; HSCT, hematopoietic stem cell transplant recipient; IC, invasive candidiasis; ICU, intensive care unit; IFI, invasive fungal infection; LFA/LFD, lateral flow assay/device; LOS, late-onset sepsis; PCR, polymerase chain reaction; VAP, ventilator-associated pneumonia.

**Table 3:** Core AFSP-Dx indicators to measure and audit diagnostic failure in IFIs [6,19,20,31,37,45,57,58,59,60,72,74,75,77,78,86,87,88,89,100,101,102].

Domain	Indicator	Suggested Metric	Operational Interpretation
Clinical recognition	Time to documented suspicion of IFI	Median number of hours from onset of the clinical syndrome to the first note including IFI in the differential diagnosis.	Evaluates whether the team recognizes the possibility of IFI early in at-risk patients.
Diagnostic process	Time to first adequate specimen	Median number of hours until blood cultures, respiratory specimen, tissue, sterile fluid, or another useful specimen according to the clinical setting.	Identifies delays in sampling and failures in diagnostic activation.
Sampling quality	Proportion of adequate specimens obtained before antifungal therapy	Percentage of episodes with a useful specimen obtained before treatment initiation, when clinically feasible.	Measures diagnostic timeliness without delaying therapy in unstable patients.
Specific tools	Use of a specific test when justified by the syndrome	Percentage of eligible episodes with GM, BDG, CrAg, endemic mycosis antigen testing, PCR, LFA/LFD, *T2Candida*, or rapid molecular panels from positive blood cultures, according to protocol.	Evaluates whether the diagnostic algorithm selects the appropriate test for the probable pathogen and the clinical setting.
Treatment	Time to appropriate antifungal therapy	Median number of hours until initiation of an active antifungal agent at an appropriate dose against the pathogen ultimately identified or considered most likely.	Evaluates loss of the therapeutic window and initial appropriateness of treatment.
Therapeutic optimization	Indicated, performed, and actionable TDM	Percentage of episodes with indicated and performed TDM; proportion of adjustments derived from suboptimal or potentially toxic levels, especially with triazoles.	Evaluates whether antifungal exposure is optimized to avoid underdosing, preventable drug toxicity, or misinterpretation of therapeutic failure.
Source control	Time to CVC removal, drainage, debridement, or surgery	Median number of hours from suspicion/confirmation to the indicated intervention.	Measures integration between diagnosis and definitive management.
Antifungal use	Empiric therapy >72 h without documented IFI	Percentage of patients treated empirically without subsequent evidence of IFI.	Indicator of persistent diagnostic uncertainty and opportunity for antifungal de-escalation.
Stewardship	Antifungal de-escalation or discontinuation at 72–96 h when applicable	Percentage of eligible patients with a documented decision.	Evaluates whether diagnostic reassessment modifies therapeutic management.
Microbiology	Time to identification at the species or species complex level	Median number of hours from positive specimen to final identification.	Measures the etiologic depth of diagnosis, especially in non-*albicans* species, molds, and emerging pathogens.
Susceptibility/resistance	Antifungal susceptibility testing or molecular resistance detection performed when clinically relevant	Percentage of eligible episodes with documented antifungal susceptibility testing or resistance marker.	Evaluates the ability to detect resistance or potentially inactive therapy.
Alert pathogens	Identification and notification of pathogens with clinical or epidemiologic implications	Time to notification and percentage of cases with documented action.	Evaluates the institutional response to *C. auris*, resistant *N. glabratus*, azole-resistant *A. fumigatus*, Mucorales, or emerging molds.
Breakthrough events	IFI during antifungal prophylaxis or therapy	Number or rate per exposed patient.	Identifies preventive failure, pathogen outside routine coverage, or emerging resistance.
Clinical outcomes	30-day mortality in proven/probable IFI	Percentage of cases, ideally adjusted for severity.	Measures the final clinical outcome associated with the diagnostic-therapeutic process.
Efficiency	Antifungal DOT per 1000 patient-days	Overall rate and rate by antifungal class.	Allows monitoring of antifungal consumption, interpreted together with indication, diagnosis, TDM when appropriate, and antifungal de-escalation.

Note: This table proposes core indicators for institutional AFSP-Dx monitoring. Their application should be adapted to local epidemiology, diagnostic availability, and the population served; results should be interpreted together with the clinical indication, sampling quality, etiologic identification, antifungal susceptibility, antifungal exposure, and clinical outcomes.

**Table 4:** Institutional AFSP-Dx interventions and auditable indicators to reduce diagnostic failure in IFIs [6,19,20,31,37,45,57,58,59,60,72,74,75,77,78,83,86,87,88,89,100,101,102,147,174].

AFSP-Dx Intervention	Responsible Team	Diagnostic Objective	Expected Benefit	Auditable Indicator	Priority
Population-specific order sets—ICU, hematology/HSCT, and neonatology	Infectious diseases, clinical pharmacy, microbiology/mycology, and clinical services	Standardize initial diagnostic activation according to risk and clinical setting.	Earlier specimen collection, lower variability, and improved test selection.	Time to first adequate specimen; adherence to the initial bundle.	High
Structured review at 48–72 h of all empiric antifungal therapy	Infectious diseases, the AFSP team, and clinical pharmacy	Integrate clinical evolution, microbiology, biomarkers, imaging studies, molecular/rapid tests, and updated pretest probability.	Reduced unnecessary exposure and greater opportunity for adjustment, de-escalation, or discontinuation of antifungal therapy.	Proportion of empiric antifungal therapies with documented review; rate of antifungal de-escalation at 72–96 h.	High
Immediate alert for blood culture with yeasts	Microbiology laboratory, infectious diseases, and treating team	Accelerate recognition and comprehensive management of candidemia.	More rapid initiation of appropriate therapy, source control, and evaluation of complications.	Time from blood culture positivity to notification, therapeutic optimization, and CVC evaluation.	High
Urgent mucormycosis pathway	Infectious diseases, surgery, radiology, pathology, and microbiology/mycology	Prioritize tissue diagnosis, evaluation of anatomic extent, and therapy active against Mucorales.	Reduced delay in surgery, source control, and appropriate therapeutic modification.	Time from suspicion to biopsy/tissue sampling; time to antifungal therapy active against Mucorales.	High
ICU-associated aspergillosis module	Infectious diseases, ICU, microbiology, and radiology	Differentiate respiratory colonization from invasive disease in critically ill patients.	Reduced underdiagnosis and reduced overtreatment due to isolated respiratory findings.	Proportion of respiratory *Aspergillus* isolates with structured evaluation; time to useful respiratory specimen.	High
CrAg and endemic mycoses module	Infectious diseases, microbiology/mycology, and clinical services	Activate specific testing when the clinical syndrome or epidemiology suggests cryptococcosis or endemic mycoses.	Reduced diagnostic omission in subacute meningitis, pulmonary/disseminated disease, or patients from endemic areas.	Proportion of eligible patients with CrAg, *Histoplasma* antigen, serology, or PCR ordered according to protocol.	Moderate-high
Molecular diagnostics and rapid testing module	Microbiology/mycology, infectious diseases, and clinical pharmacy	Define the rational use of targeted PCR, panfungal PCR/sequencing, LFA/LFD, T2*Candida*, and rapid molecular panels from positive blood cultures.	Reduced time to identification and improved selection of therapy or antifungal de-escalation.	Time to result; proportion of ordered tests with documented indication; impact on therapeutic decision-making.	Moderate-high
Identification, antifungal susceptibility, and alert pathogen module	Microbiology/mycology, infectious diseases, clinical pharmacy, and hospital epidemiology	Detect species with therapeutic implications, resistance, membership in cryptic complexes, or epidemiologic impact.	More precise therapy, detection of breakthrough IFIs, and improved institutional control.	Time to identification at the species or species complex level; proportion of cases with indicated and performed antifungal susceptibility testing.	High
Antifungal exposure optimization and TDM module	Clinical pharmacy, infectious diseases, microbiology/mycology, and clinical services	Optimize antifungal exposure when treatment is continued, especially with triazoles, relevant drug interactions, suspected drug toxicity, or therapeutic failure.	Reduced preventable drug toxicity, lower suboptimal exposure, and improved differentiation between pharmacokinetic failure, microbiologic failure, and incomplete diagnosis.	Proportion of patients with indicated and performed TDM; time to result; proportion of adjustments derived from suboptimal or potentially toxic levels.	Moderate-high
Monthly summary of AFSP-Dx metrics	Hospital epidemiology, quality, AFSP, and clinical services	Monitor diagnostic timeliness, appropriate treatment, source control, and clinical outcomes.	Continuous improvement and detection of diagnostic bottlenecks.	Time to suspicion, specimen collection, therapy, source control, DOT, breakthrough events, actionable TDM, and 30-day mortality.	Moderate
Population-focused clinical education	Infectious diseases, microbiology, pharmacy, nursing, and service leaders	Correct frequent diagnostic errors according to the population.	Improved interpretation of tests, greater adherence to care bundles, and lower clinical variability.	Educational coverage; changes in bundle adherence and indicators after the intervention.	Moderate

Note: This table summarizes institutional AFSP-Dx interventions adaptable to local epidemiology, diagnostic resources, and the populations served. The proposed priorities should be interpreted as a practical guide to orient auditable processes, not as universal requirements. AFSP, antifungal stewardship program; AFSP-Dx, diagnostic-centered antifungal stewardship program; CrAg, cryptococcal antigen; CVC, central venous catheter; DOT, days of therapy; HSCT, hematopoietic stem cell transplant recipient; ICU, intensive care unit; IFI, invasive fungal infection; LFA/LFD, lateral flow assay/device; PCR, polymerase chain reaction; TDM, therapeutic drug monitoring.

## Data Availability

No new data were created or analyzed in this study. Data sharing is not applicable to this article.
